# Synthetic Phosphodiester‐Linked 4‐Amino‐4‐deoxy‐l‐arabinose Derivatives Demonstrate that ArnT is an Inverting Aminoarabinosyl Transferase

**DOI:** 10.1002/cbic.201900349

**Published:** 2019-10-22

**Authors:** Charlotte Olagnon, Julia Monjaras Feria, Clemens Grünwald‐Gruber, Markus Blaukopf, Miguel A. Valvano, Paul Kosma

**Affiliations:** ^1^ Department of Chemistry University of Natural Resources and Life Sciences–Vienna Muthgasse 18 1190 Vienna Austria; ^2^ Wellcome-Wolfson Institute of Experimental Medicine Queen's University Belfast 97 Lisburn Road BT9 7BL Belfast UK

**Keywords:** *Burkholderia*, carbohydrates, glycolipids, glycosyl transferases, lipopolysaccharide

## Abstract

4‐Amino‐4‐deoxy‐l‐arabinopyranose (Ara4N) residues have been linked to antibiotic resistance due to reduction of the negative charge in the lipid A and core regions of the bacterial lipopolysaccharide (LPS). To study the enzymatic transfer of Ara4N onto lipid A, which is catalysed by the ArnT transferase, we chemically synthesised a series of anomeric phosphodiester‐linked lipid Ara4N derivatives containing linear aliphatic chains as well as *E*‐ and *Z*‐configured monoterpene units. Coupling reactions were based on sugar‐derived *H*‐phosphonates, followed by oxidation and global deprotection. The enzymatic Ara4N transfer was performed in vitro with crude membranes from a deep‐rough mutant from *Escherichia coli* as acceptor. Product formation was detected by TLC and LC‐ESI‐QTOF mass spectrometry. Out of seven analogues tested, only the α‐neryl derivative was accepted by the *Burkholderia cenocepacia* ArnT protein, leading to substitution of the Kdo_2_‐lipid A acceptor and thus affording evidence that ArnT is an inverting glycosyl transferase that requires the *Z*‐configured double bond next to the anomeric phosphate moiety. This approach provides an easily accessible donor substrate for biochemical studies relating to modifications of bacterial LPS that modulate antibiotic resistance and immune recognition.

## Graphical abstract

**Stereochemistry matters**: Out of seven synthesized phospholipid‐connected 4‐amino‐4‐deoxy‐l‐arabinose derivatives, only the equatorial neryl derivative was accepted by the Ara4N transferase from *B. cenocepacia*, leading to incorporation of one and two Ara4N residues into a deep rough mutant lipopolysaccharide from *E. coli*.

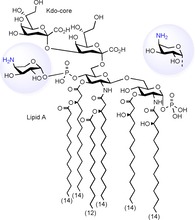

## Introduction

Rising rates of infection by drug‐resistant Gram‐negative bacteria are becoming a global challenge for public health systems and the economy.^1^ The outer leaflet of the Gram‐negative bacterial cell envelope acts as an efficient permeability barrier and harbours major surface components, such as lipopolysaccharide (LPS), that interact with both innate and adaptive immune systems.^2^ In general, the LPS architecture includes the O‐antigenic polysaccharide, the oligosaccharide core region and the lipid A domain.^3^ Lipid A is composed of an acylated bisphosphorylated β‐(1→6)‐linked glucosamine disaccharide and is a major signalling molecule eliciting innate immune responses.^4^ The lipid A is usually extended by 3‐deoxy‐d‐*manno*‐oct‐2‐ulosonic acid (keto‐deoxyoctosonate, Kdo) residues, which in several bacterial strains are partially replaced by the Kdo‐isosteric and acid‐stable 3‐hydroxy derivative d‐*glycero*‐d‐*talo*‐oct‐2‐ulosonic acid (keto‐octosonic acid, Ko).^5^ The pronounced negative charge imparted by the phosphate and carboxylate groups in LPS serves as target for potent cationic antimicrobial peptides (CAMPs) and lipopeptides such as polymyxin B (PmB) or colistin.

**Scheme 1:**
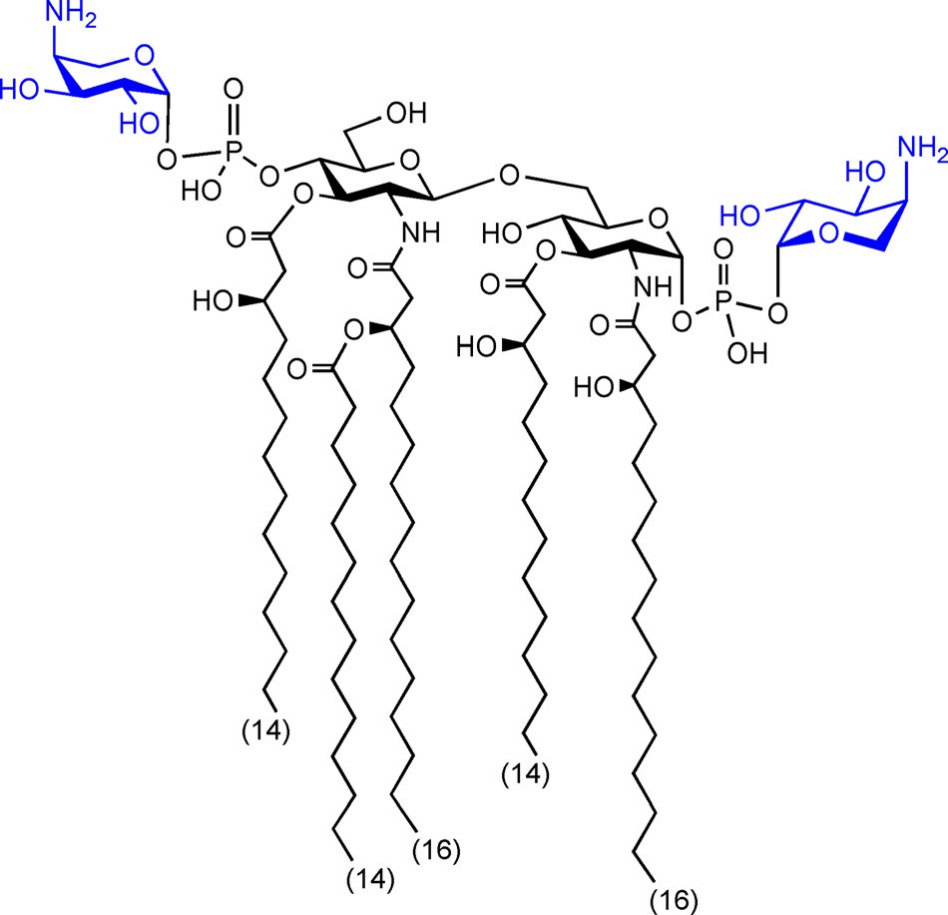
Ara4N‐modified lipid A structure of *B. multivorans*.

These antibiotics are used as last‐resort treatments against multidrug‐resistant bacteria.^6^, ^7^ Many bacteria, however, develop resistance mechanisms that counteract the action of CAMPs and polymyxins through covalent modification of the Kdo/Ko and lipid A with positively charged appendices such as phosphoethanolamine, 2‐amino‐2‐deoxy‐sugars or 4‐amino‐4‐deoxy‐l‐arabinose (Ara4N).^8^ For example, both the ester‐linked and the glycosidically linked phosphate groups of the *Burkholderia multivorans* lipid A may be substituted by Ara4N residues (Scheme 1).^9^ Ara4N substitution is implicated as a major cause of antibiotic resistance in many bacteria, such as *Burkholderia cenocepacia*, *Klebsiella pneumoniae*, *Pseudomonas aeruginosa*, *Yersinia pestis* and *Yersinia enterocolitica*, *Proteus mirabilis* and *Salmonella enterica* serovar Typhimurium.6a, 10 Inhibition of the biosynthetic pathway involved in the formation of the activated sugar and blocking of the Ara4N transfer reaction, which is catalysed by the ArnT transferase, should thus restore the antimicrobial efficacy of PmB and CAMPs.

As shown by Raetz, α‐configured undecaprenyl‐phosphate Ara4N (UndP‐Ara4N) serves as the donor substrate for ArnT, a membrane‐embedded lipid‐to‐lipid glycosyl transferase.^11^ ArnT from *S. enterica* serovar Typhimurium and from *B. cenocepacia* have been partly characterized^12^, ^13^, ^14^, ^15^ and a 2.8 Å crystal structure of ArnT from *Cupriavidus metallidurans*, including a cocrystal structure obtained with UndP as the ligand, has been reported.^16^ Unfortunately, the binding site interactions with the ArnT portion were modelled on the basis of docking studies that used the incorrect anomeric configuration of the Ara4N residue, displaying the sugar part in the inverted ^1^ *C*_4_ chair conformation.^16^ Thus, the underlying ArnT reaction mechanism and detailed knowledge relating to binding of donor and acceptor substrates are still lacking. It is also unknown whether a single ArnT enzyme acts as a multifunctional transferase (substituting both lipid A and the inner core sugars) or whether separate Ara4N transferases are needed for glycosylation at O‐8 of Kdo and of Ko.

Only minor amounts of the native Ara4N‐P‐undecaprenyl donor (0.2 mg) had previously been isolated from bacteria.^11^ Thus, chemical syntheses of Ara4N donor structures and potential acceptor substrates are needed for study of the underlying enzymatic mechanisms and transport/translocation processes, in particular in order to obtain X‐ray structures with the liganded sugar and binding data in the presence of substrates and substrate analogues, thereby enabling the rational design of efficient enzyme inhibitors. Inhibitors of the enzymatic formylation of Ara4N‐UDP (catalysed by ArnB) with activity in the high micromolar range had previously been prepared, but were not sufficiently active to confer PmB sensitivity.^17^ We report here the chemical synthesis of seven phosphodiesteractivated 4‐amino‐4‐deoxy‐l‐arabinose derivatives, the development of an assay to monitor the in vitro transfer reaction catalysed by ArnT from *B. cenocepacia*, and the product characterization by LC‐ESI‐QTOF mass spectrometry.

## Results and Discussion

### Synthesis of Ara4N enzyme donors

**Scheme 2:**
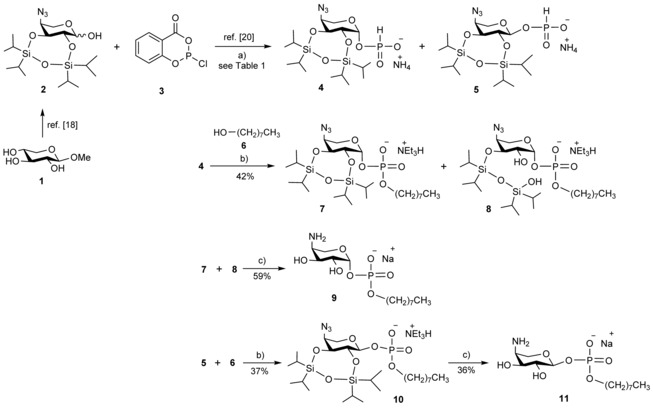
Synthesis of anomeric *H*‐phosphonates and octyl phosphodiester derivatives **9** and **11**. a) Pyridine, THF, RT, then 1 m aq. NH_4_HCO_3_; b) pivaloyl chloride, 2,6‐lutidine, RT, then aq. I_2_, 0 °C; c) triethylamine trihydrofluoride (TREAT), THF, RT, then CaCO_3_, MeOH, RT, then Me_3_P, THF, aq. NaOH.

For the synthesis of the Ara4N donor, the precursor material was readily prepared in multigram quantities from methyl β‐d‐xylopyranoside (**1**, Scheme 2).^18^ A 4‐azido group was chosen as the amino group precursor, and the 2‐*O*‐ and 3‐*O*‐positions were protected with a tetraisopropyl‐disiloxane‐1,3‐diyl (TIPDS) group instead of benzyl groups, to avoid any need for hydrogenolytic deblocking steps that would not be compatible with the prenyl double bonds. For the coupling steps we relied on the preparation of the anomeric *H*‐phosphonates that are frequently used for the assembly of phosphodiester‐linked glycans because these are more stable than intermediate phosphoramidites.^19^ We had previously optimized the formation of the axial β‐anomeric product needed for the connection of Ara4N *H*‐phosphonate to the reducing end of *Burkholderia* lipid A.^20^ Structural evidence for the α‐l configuration of the Und‐P‐Ara4N donor substrate leading to the β configuration of Ara4N residues when linked to lipid A as well as to Kdo and Ko suggested that the α‐configured derivatives should serve as the primary targets for the enzymatic transfer.^21^ To achieve preferred formation of the equatorial *H*‐phosphonate in the reaction with salicyl chlorophosphite, slow addition of reagent over a long time period was essential to allow for a kinetically controlled reaction at the more nucleophilic equatorial hydroxy group (Scheme 2, Table 1).^22^, ^23^

**Table 1:** Stereoselective synthesis of 2,3‐*O*‐TIPDS‐protected β‐ and α‐Ara4N *H*‐phosphonates **4** and **5**.

	[**2**] [mol L^−1^]	[**3**] equiv h^−1[a]^	Conversion^[b]^ [%]	α/β ratio
1	0.04	0.4	97	3.3
2^[c]^	0.04	0.4	92	0.6
3	0.07	0.4	96	2.8
4	0.02	0.4	96	2.3
5	0.04	0.2	96	4.6
6	0.04	0.1	93	6.1

[a] Four equivalents of **3** were added at RT. [b] Calculated from integration values of ^1^H NMR signals. [c] Reaction was performed at 0 °C.

To achieve significant enrichment of the α‐isomer **5**, the optimum addition rates should be in a range between 0.1 and 0.2 equivalents of the chlorophosphite reagent per hour (entries 5 and 6).

Next, the formation of the phosphodiester derivative was elaborated through the use of octan‐1‐ol (**6**) and the β‐anomeric *H*‐phosphonate **4** in model reactions. Activation of the *H*‐phosphonate **4** was first attempted with pivaloyl chloride in pyridine followed by oxidation with aqueous iodine.^24^ In addition to product **7**, a major by‐product corresponding to an *N*‐pyridinium glycoside was isolated. To prevent this side reaction, sterically hindered 2,6‐dimethylpyridine as a base with enhanced nucleophilicity was then used throughout the coupling reactions. In this way, product mixtures of **7** and ring‐opened derivative **8** were isolated in combined yields of 40–42 %. Extending the reaction time for the coupling step, however, led to increased formation of the ring‐opened form. To increase the stability of the anomeric phosphodiester, the products were converted into their triethylammonium salts. The formation of the phosphodiester was confirmed through its ^31^P NMR chemical shift at −2.13 ppm. The site of the silyl ether cleavage in compound **8** could be unambiguously established by performing a ^1^H/^29^Si HMBC experiment, in which only H‐3 at 4.52 ppm was observed to correlate with a ^29^Si signal at −15.9 ppm, whereas the H‐3 and H‐2 protons of compound **7** at 4.32 and 4.03 ppm, respectively, showed connectivity to ^29^Si NMR signals at −8.5 and −9.0 ppm. Because global deprotection would include removal of the silyl group anyway, the mixture of **7** and **8** was directly employed for the ensuing deprotection steps. Removal of the TIPDS group was achieved by treatment with triethylamine trihydrofluoride (TREAT) followed by removal of the fluoride ions with calcium carbonate. Staudinger reduction of the 4‐azido group with trimethylphosphine in the presence of aqueous THF and NaOH gave phosphodiester **9** as its sodium salt after final purification on Bio‐Gel P2 in 59 % yield over two steps.

**Scheme 3:**
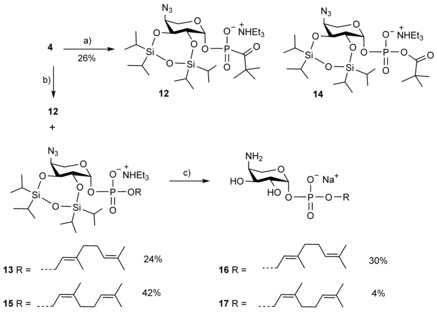
Synthesis of β‐geranyl and β‐neryl derivatives **16** and **17**, respectively. a) PivCl, 2,6‐lutidine, RT, then aq. I_2_, 0 °C; b) ROH, PivCl, 2,6‐lutidine, RT; aq. I_2_, 0 °C; c) TREAT, THF, RT; CaCO_3_, MeOH, RT, then Me_3_P, THF, aq. NaOH.

Coupling between *H*‐phosphonate **4** and geraniol (Scheme 3), however, gave additional by‐products, leading to mixtures that required multiple chromatographic purification steps in order to provide the target derivative. Several side reactions, most probably involving mixed anhydrides and *H*‐pyrophosphonates as reactive intermediates, have previously been reported in pivaloyl‐chloride‐mediated *H*‐phosphonate diester synthesis.^25^ We explored the activation pathway of *H*‐phosphonate **4** in the absence of the alcoholic component by use of ^31^P NMR monitoring and isolation of the main product after iodine‐mediated oxidation. *H*‐Phosphonate **4** reacted with pivaloyl chloride in CDCl_3_ in the presence of 2,6‐lutidine within 15 min, accompanied by the appearance of two ^31^P NMR signals (at −6.50 and −8.04 ppm) that did not change over a period of 2 h at room temperature. Upon oxidation, a main signal at −6.05 ppm was seen, and gradually additional minor upfield‐shifted signals appeared. As the main product, the acyl phosphonate **12** was then isolated by chromatography. The structure assignment of **12** was based on the significantly downfield‐shifted C=O signal at 218.7 ppm, with *J*_C,P_=154.6 Hz; this is in close agreement with published NMR data for acyl *H*‐phosphonates.^26^ The structure was also confirmed by LC‐ESI‐MS data that revealed a molecular ion of *m*/*z* 566.2488, thus suggesting a structure related to ester **14** but with loss of one oxygen atom (Scheme 3).

**Scheme 4:**
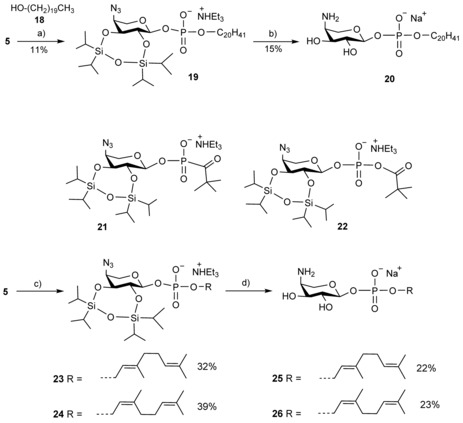
Synthesis of α‐geranyl and α‐neryl derivatives. a) PyTP, BSA, then CSO; b) TREAT, THF, RT; then CaCO_3_, MeOH, RT; then DTT; c) ROH, PivCl, 2,6‐lutidine, RT; aq. I_2_, 0 °C; d) TREAT, THF, RT; CaCO_3_, MeOH, RT, then Me_3_P, THF, aq. NaOH.

Formation of this by‐product might be explained in terms of phosphite formation induced by 2,6‐lutidine followed by an Arbusov reaction with the acyl chloride. Since the isolated yields of the phosphodiester derivatives were modest, a different activation and oxidation protocol was then tested with *H*‐phosphonate **5** and eicosan‐1‐ol (**18**, Scheme 4).

Coupling of *H*‐phosphonate **5** was performed with chlorotripyrrolidinophosphonium hexafluorophosphate (PyTP) followed by conversion into the silylated phosphite triester with bis(trimethylsilyl)acetamide (BSA) and subsequent oxidation with camphorsulfonic acid (CSO).^27^, ^28^ This approach, however, produced the diester derivative **19** only in a poor yield. Compound **19** was finally converted into the deprotected derivative **20**. In view of these results, we again opted for the pivaloyl chloride activation for the synthesis of the α‐geranyl and α‐neryl derivatives **23** and **24**, respectively. Similarly to the outcome for the β‐analogues **13** and **15**, the condensation step also led to formation of minor amounts of O‐ and C‐linked pivaloyl derivatives **21** and **22**. Removal of these by‐products was effectively accomplished upon deprotection of the mixture and azide reduction, which eventually afforded the diester derivatives **25** and **26** in their pure states after chromatography on BioGel P‐2.

### Enzymatic assay for the ArnT‐catalysed reaction

**Figure 1:**
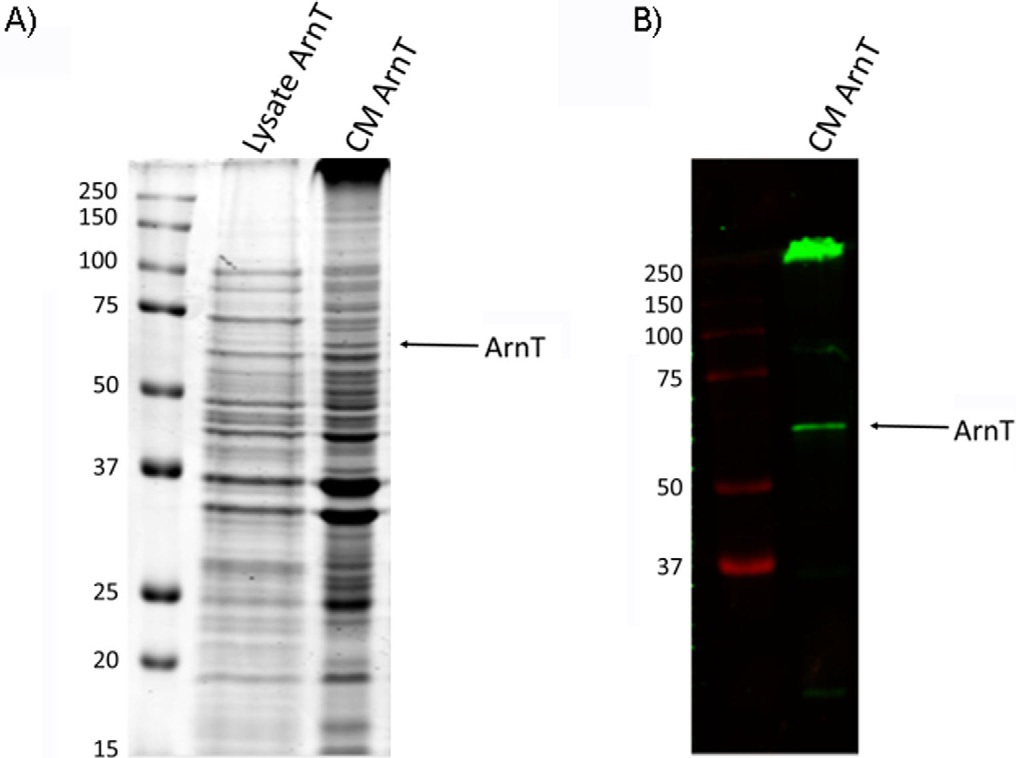
ArnT from *B. cenocepacia* expressed in *E. coli* membranes. A) ArnT‐FLAG‐His_10_ (71.8 kDa) in total cell lysate and after total membrane preparation was analysed by SDS‐PAGE followed by CBB staining. B) Immunodetection of ArnT‐FLAG‐His_10_ in total membrane with anti‐FLAG antibodies. CM: crude membrane.

ArnT from *B. cenocepacia* was expressed in *Escherichia coli* as a FLAG‐His_10_‐tagged protein as previously described, and crude membranes were prepared.14a The presence of ArnT in the membranes was confirmed by SDS‐PAGE followed by Coomassie Brilliant Blue (CBB) staining and western blot with use of anti FLAG antibodies (Figure 1).

**Figure 2:**
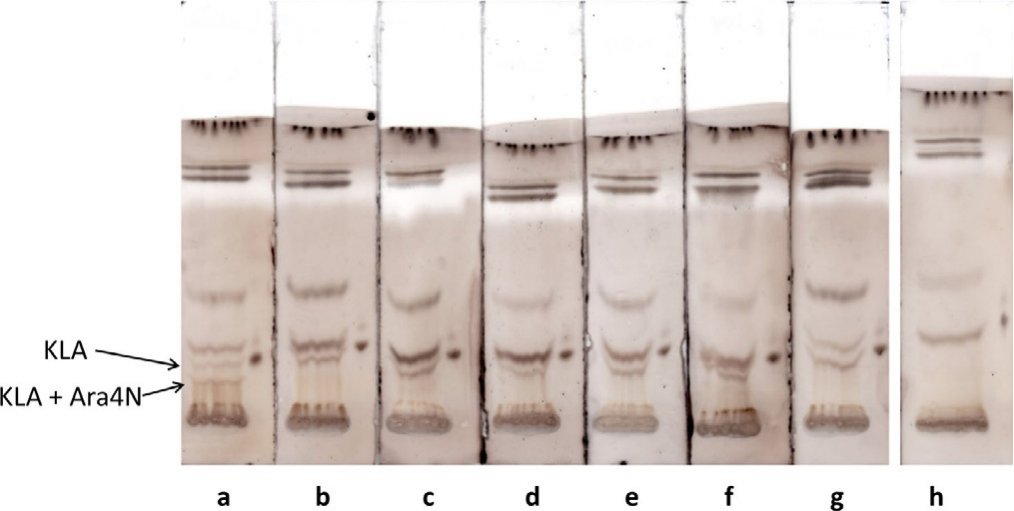
In vitro detection of *B. cenocepacia* ArnT activity with α‐Ara4N‐neryl phosphate **26** by TLC. ArnT from *B. cenocepacia* was assayed with use of KLA (30 μm) as acceptor, crude membranes from ArnT expression (1 mg mL^−1^) as source of l‐Ara4N transferase and 150 μm synthetic donor substrates (lane a: **26**, lane b: **17**, lane c: **25**, lane d: **16**, lane e: **11**, lane f: **9**, lane g: **20**. Acceptor KLA is visible as spot on the right‐hand lane on each TLC plate.). Lane h shows a control assay containing **26** but lacking acceptor KLA. Reaction mixtures were incubated at 30 °C for 17 h. Spots were stained with anisaldehyde/H_2_SO_4_.

Prior to testing of the substrates for the enzymatic reaction, a modified assay based on the commercial deep rough type LPS containing lipid A and the α‐(2→4)‐linked Kdo disaccharide, termed KLA, was developed. The assay should be compatible with the phenol/water extraction conditions used for LPS isolation and the hydrolytic lability of phosphodiester‐linked 4‐aminoarabinosyl substituents.^29^ After extensive exploration of conditions and isolation protocols we focused on monitoring of the glycosyl transfer reaction by preparative TLC,^30^ which allowed for visual inspection of product formation, and subsequent structural analysis of products by LC‐ESI‐QTOF mass spectrometry. Further, this approach also allows for removal of residual surfactants and extraction media arising from the cell‐lysing procedure and LPS isolation. The donor substrates were treated with KLA in the presence of ArnT for 17 h at 30 °C. The samples were concentrated, dissolved in chloroform/methanol 4:1, spotted in 5 μL portions onto prewashed silica gel 60 TLC plates and developed with CHCl_3_/pyridine/88 % HCOOH/water 50:50:16:5 (Figure 2). The lipid fractions were recovered from the plates through a single acidic Bligh–Dyer extraction followed by centrifugation and treatment with a two‐phase Bligh–Dyer extraction. They were then subjected to LC‐ESI‐QTOF analysis.

The glycosyl acceptor KLA could be detected in an *R*_f_ range of ≈0.24–0.28 in all experiments. Formation of a new compound at *R*_f_ ≈0.15 and reduction of the intensity of acceptor substrate KLA was observed in the assay with the α‐neryl derivative **26** (Figure 2, lane a), whereas none of the other substrates showed product formation.

**Figure 3:**
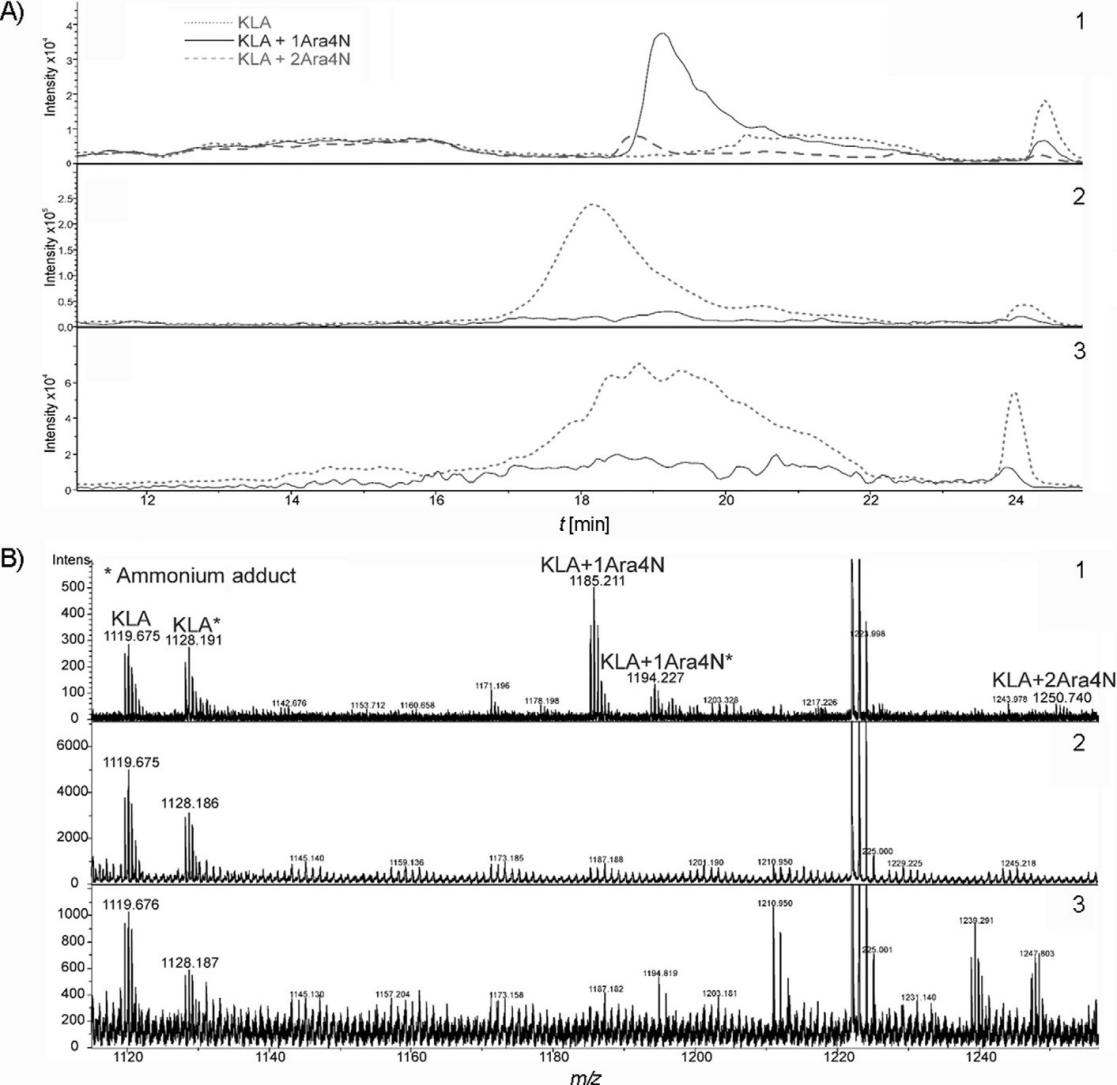
In vitro detection of *B. cenocepacia* ArnT activity with α‐Ara4N‐neryl phosphate **26** by LC‐ESI‐MS. A) LC‐MS chromatograms of purified KLA derivatives from assays containing 1) α‐Ara4N‐neryl phosphate **26**, 2) β‐Ara4N‐neryl phosphate **17** and 3) α‐Ara4N‐geranyl phosphate **25** as donors. KLA (EIC= 1119.674, 1120.175, 1120.677, 1128.187, 1128.688, 1129.190), KLA+1 Ara4N (EIC=1185.203, 1185.704, 1186.206, 1193.716, 1194.217, 1194.719) and KLA+2 Ara4N (EIC=1250.733, 1251.235, 1251.737,1252.238). B) MS spectra of purified KLA derivatives from assays containing 1) α‐Ara4N‐neryl phosphate **26**, 2) β‐Ara4N‐neryl phosphate **17** and 3) α‐Ara4N‐geranyl phosphate **25** as donors. KLA: calcd for C_110_H_202_N_2_O_39_P_2_+2 H^2+^ [*M*+2 H]^2+^: 1119.675. KLA+1 Ara4N: calcd for C_115_H_211_N_3_O_42_P_2_+2 H^2+^ [*M*+2 H]^2+^: 1185.20. KLA+2 Ara4N: calcd for C_120_H_220_N_4_O_45_P_2_+2 H^2+^ [*M*+2 H]^2+^: 1250.733.

These results were confirmed by LC‐ESI‐MS analysis of lipid fractions extracted from the TLC plates after using the anomeric neryl derivatives **26** and **17** and the α‐geranyl derivative **25** as substrates. These selected donors should clarify the dependence of the ArnT reaction with respect to the anomeric configuration of the arabinosyl unit, as well as to the stereochemistry of the monoterpene unit. To achieve sufficient concentration of products, assays were run in duplicate in the cases of compounds **17** and **25** and were repeated seven times in that of compound **26**. TLC‐extracted samples were subjected to C5 reversed‐phase HPLC separation and eventually measured in the positive mode with the ESI‐QTOF instrument. HPLC profiles and spectra are presented in Figure 3. Lipid extracts from reactions with **17** and **25** gave only mass data corresponding to the unreacted acceptor KLA substrate, with monoisotopic *m*/*z* values of 1119.674 for the doubly charged molecular ion [*M*+2 H^+^]^2+^ and 1128.187 for the ammonium adduct [*M*+NH_4_^+^+H^+^]^2+^ (Figure 3.)

The formation of the Ara4N‐KLA in the reaction with **26** was evident from the appearance of peaks at higher molecular mass (1185.705) corresponding to the transfer of one Ara4N residue. In three assays a very minor peak (3–5.5 %) was detected at *m*/*z* 1250.740, indicative of the transfer of a second Ara4N residue. For quantification of the Ara4N‐KLA derivative formed in the reaction with **26**, the areas of the ion chromatograms from the first four isotopic peaks of KLA and product were summed and compared, leading to an approximate range of 40 to 65 % conversion of **26**. The attachment site of the Ara4N residue could not be determined. Literature data for lipid A modification in the presence of the Kdo core, however, suggest preferential formation of the phosphodiester‐linked Ara4N at the 4′ position of lipid A.^11^, ^31^

The ArnT reaction was thus only productive with the α‐neryl derivative **26**, as shown by TLC and LC‐ESI‐MS analysis. This was evidence that the enzyme is specific for the α‐anomeric configuration of the l‐arabinosyl unit and the *Z* configuration of the lipid part. Dependence of glycosyl transferases on lipid chain length and double bond configuration has previously been reported for related pyrophosphate substrate derivatives. The lipid carrier specificity of the *N*‐glycosylation enzyme PglB from *Campylobacter jejuni* has been studied, for example, revealing that the enzyme is not active with C10 and C15 lipid appendages but readily transfers substrates with four prenyl units, provided that the two proximal units are *cis*‐configured.^32^ In contrast, the membrane‐associated UDP GlcNAc transferase MurG involved in peptidoglycan synthesis has a more relaxed acceptor substrate specificity, with a neryl derivative having previously been observed to be accepted by the glycosyltransferase.^33^ A similar preference was reported for a galactofuranosyl transferase from *Mycobacterium tuberculosis* that accepted a *C*‐linked neryl derivative as substrate.^34^

The synthetic α‐neryl donor substrate for ArnT reactions should be useful for study not only of the modification of the phosphate groups in the lipid A domain, but also of Ara4N glycoside formation in the core region of several Enterobacteriaceae. It remains to be determined whether chain elongation including three or four prenyl units will be needed for the donor substrates for optimum enzymatic transfer.

## Conclusion

A 4‐amino‐4‐deoxy‐l‐arabinosyl transferase from *B. cenocepacia* was successfully expressed and used in a crude membrane preparation to test the substrate specificities of a small library of lipid/phosphate‐linked 4‐amino‐4‐deoxy‐l‐arabinosyl derivatives. The phosphodiester derivatives were obtained in modest yields from 2,3‐*O*‐TIPDS‐protected 4‐azido‐substituted *H*‐phosphonate derivatives by pivaloyl‐chloride‐promoted coupling steps followed by oxidation. Formation of by‐products in the activation step could be analysed, and azide reduction and final purification steps were optimised to give the deprotected donor substrates. Glycosyl transfer reactions were monitored by TLC, followed by structural characterization of the TLC extracts. The ArnT reaction was only productive with the α‐neryl derivative, as shown by LC‐ESI‐MS analysis; this was therefore evidence that the enzyme is specific for the α‐anomeric configuration of the arabinosyl unit and the *Z* configuration of the lipid part. A simple synthetic donor substrate for testing enzymatic pathways involved in antibiotic resistance mechanisms and crystallographic studies is therefore now available without the necessity to incorporate oligoprenyl lipid chains.

## Experimental Section

**General methods**: All reactions were carried out in oven‐dried glassware. Solvents and reagents were purchased from commercial suppliers and used as provided without further purification unless stated otherwise. Solvents (THF, toluene, CH_2_Cl_2_) were dried over activated molecular sieves (4 Å). 2,6‐Lutidine was distilled over CaH_2_ and stored over CaH_2_. Cation‐exchange resin DOWEX 50 H^+^ was regenerated by consecutive washing with HCl (3 m), water and dry MeOH. Concentration of organic solutions was performed under reduced pressure at <40 °C. Optical rotation was measured with an Anton Paar MCP100 Polarimeter at 20 °C. Reactions were followed by TLC with Merck plates: generally on 5×10 cm, layer thickness 0.25 mm, silica gel 60F_254_, or alternatively on HPTLC plates with 2.5 cm concentration zone (Merck). Spots were visualized with UV (254 nm) and/or by anisaldehyde/H_2_SO_4_ staining. Preparative chromatography was performed either with silica gel (0.040–0.063 mm) or with a flash‐purification system (Interchim, PuriFlash 4125). NMR spectra were recorded with a Bruker Avance III 600 instrument (^1^H at 600 MHz, ^13^C at 151 MHz, ^31^P at 243 MHz, ^29^Si at 119.2 MHz) and use of standard Bruker NMR software. Chemical shifts are given in ppm downfield from SiMe_4_ using the residual peak of CDCl_3_ (7.26 for ^1^H and 77.00 for ^13^C), CD_3_OD (3.31 for ^1^H and 49.86 for ^13^C) or D_2_O (0.00 for ^1^H, external calibration to 2,2dimethyl‐2‐silapentane‐5‐sulfonic acid), 67.40 for ^13^C (external calibration to 1,4‐dioxane in D_2_O), and orthophosphoric acid (*δ*=0) for ^31^P. HRMS ESI‐TOF data were obtained with a Waters Micromass Q‐TOF Ultima Global instrument.

**Optimization of anomeric** ***H*** **‐phosphonate formation (compounds 4 and 5)**: Compounds **4** and **5** [4‐azido‐4‐deoxy‐2,3‐*O*‐(tetraisopropyldisiloxane‐1,3‐diyl)‐β‐ and ‐α‐l‐arabinopyranose hydrogen phosphonate, triethylammonium salts] were essentially prepared according to the literature and gave matching NMR data and optical rotation values.^20^ To enrich compound **5**, however, the following conditions were used: dry pyridine (13 equiv) was added to salicyl chlorophosphite (**3**, 4 equiv) in dry THF (2.52 mL mmol^−1^ **3**) at 0 °C and the mixture was stirred for 1 h at RT. The solution was transferred into a syringe and added at a rate of 0.1 equiv h^−1^ to a solution of **2** (54 mg, 0.129 mmol) in dry THF (3.5 mL). After complete addition of the activated salicyl phosphite pyridinium salt, aqueous ammonium bicarbonate solution (1 m, 1.1 mL) was added and the mixture was stirred for an additional 30 min and then diluted with EtOAc (25 mL) and washed with aqueous ammonium bicarbonate buffer (0.3 m, 15 mL). The aqueous layer was reextracted with dichloromethane (2×20 mL) and the combined organic layers were filtered over cotton. Toluene (5 mL) was added and volatiles were evaporated under reduced pressure prior to chromatographic separation according to the literature.^20^ NMR analysis of the crude product showed a **5**/**4** mixture (6.1:1).

**General Procedure A for phosphodiester synthesis**: *H*‐Phosphonate **4** or **5** was co‐evaporated with dry toluene (4×10 mL) and dried under high vacuum overnight. The acceptor alcohol (4 or 5 equiv) was pre‐dried over molecular sieves (4 Å) and added at RT under Ar to a solution of the *H*‐phosphonate in dry 2,6‐lutidine (1.0–2.4 mL/0.1 mmol). The solution was stirred for 10 min, pivaloyl chloride (2–4.5 equiv) was then added dropwise at RT, and the reaction was monitored by ^31^P NMR, by transferring aliquots (50 μL) of the reaction mixture into an NMR tube and adding CDCl_3_ (0.4 mL). The reaction mixture was cooled to 0 °C, a solution of iodine (2 %, 1.5 equiv) in 2,6‐lutidine/water (50:1, 1.0 mL/0.1 mmol) was added dropwise, and the mixture was stirred at 0 °C for 1 h. The solution was then diluted with chloroform followed by addition of 1:1 7 % aq. Na_2_S_2_O_3_/2 m triethylammonium bicarbonate (TEAB) buffer. The aqueous phase was extracted with chloroform and the combined organic phases were filtered over cotton, concentrated and co‐evaporated with toluene (10 mL) under reduced pressure. The product was purified by two to four consecutive chromatographic purification steps on silica gel [chloroform containing Et_3_N (1 %)→chloroform/MeOH 9:1+Et_3_N (1 %)]. To stabilize the phosphate bridge, purified compounds and pooled fractions were dissolved in chloroform (14 mL) and washed with TEAB buffer (0.25 m, 8 mL). The organic phase was dried over cotton and concentrated to give the stable phosphodiester derivative.

**General Procedure B for TIPDS removal**: Triethylamine trihydrofluoride (3 equiv) was added at 0 °C to a solution of phosphodiester in dry THF (2.5 mL mmol^−1^) in a screw‐top Teflon flask and the mixture was stirred at RT under Ar until complete conversion. CaCO_3_ (6 equiv) and dry THF (2.5 mL mmol^−1^) were then added. The suspension was stirred at RT for 1 h and centrifuged at 1972 *g* for 15 min. The liquid phase was recovered and lyophilised overnight to give the crude intermediate product that was directly used for the next reaction.

**General Procedure C for azide reduction**: PMe_3_ (4 equiv) was added at RT under Ar to a solution of the crude intermediate product in THF/0.1 m NaOH (1:2) and the mixture was stirred until full conversion. The solution was diluted with water and washed with chloroform. The combined organic phases were twice re‐extracted with water and stripped with Ar until the solution became clear. The aqueous phase was lyophilised and the residue was purified by gel chromatography (BioGel P‐2, eluent aq. EtOH, 5 %) and lyophilised overnight to afford the pure target phosphodiester derivative.

**Octyl 1‐*O*‐[4‐azido‐4‐deoxy‐2,3‐*O*‐(tetraisopropyldisiloxane‐1,3‐diyl)‐β‐l‐arabinopyranosyl] phosphate, triethylammonium salt** (**7**): *H*‐Phosphonate **4** (46 mg, 0.079 mmol) was co‐evaporated with dry toluene (4×10 mL) and dried under high vacuum overnight. Octan‐1‐ol **6** (25 μL, 0.159 mmol) was pre‐dried over molecular sieves (4 Å) and added to a solution of **4** in dry 2,6‐lutidine (1.9 mL). This was stirred for 10 min at RT under Ar. Pivaloyl chloride (44 μL, 0.357 mmol) was then added dropwise at RT and the reaction mixture was stirred for 46 h. The solution was cooled to 0 °C and a solution (2 %) of iodine (30.2 mg, 0.119 mmol) in 2,6‐lutidine/water (50:1, 0.8 mL) was added dropwise and stirred at 0 °C for 1 h. The solution was diluted with chloroform (50 mL) followed by addition of a mixture of aq. Na_2_S_2_O_3_ (7 %)/TEAB buffer (2 m; 1:1, 20 mL). The aqueous phase was extracted with chloroform (2×50 mL) and the combined organic phases were filtered over cotton, concentrated and co‐evaporated with toluene (7 mL) under reduced pressure. The product was purified by flash chromatography on silica gel (chloroform containing 1 % Et_3_N→9:1 chloroform/MeOH+1 % Et_3_N). Product‐containing fractions were pooled and concentrated. The residue was dissolved in chloroform (15 mL) and washed with TEAB buffer (0.25 m, 8 mL). The organic phase was dried over cotton and concentrated to give a product fraction (23.8 mg, 42 %) containing **8** (6 %). NMR data for **8**: ^1^H NMR (600 MHz, CDCl_3_): *δ*=5.59–5.56 (m, 1 H; H‐1), 4.52 (dd, *J*_4,3_=3.6, *J*_2,3_=9.8 Hz, 1 H; H‐3), 3.97 (d, *J*_5b,5a_=12.1 Hz, 1 H; H‐5a), 3.94–3.82 (m, 4 H; OC*H_2_*, H‐4, H‐2), 3.61 (d, 1 H; H‐5b), 3.07–3.03 (m, 6 H; 3×C*H_2_*N)_,_ 1.63–1.57 (m, 2 H; OCH_2_ *C*H_2_), 1.33–1.26 (m, 19 H; 5×CH_2_, 3×C*H_3_*CH_2_N), 1.10–1.01 (m, 28 H; TIPDS), 0.89–0.86 ppm (m, 3 H; CH_3_); ^13^C NMR (151 MHz, CDCl_3_): *δ*=97.70 (d, *J*_P,1_=7.1 Hz, C‐1), 70.95 (C‐3), 70.15 (d, *J*_P,2_=5.5 Hz, C‐2), 66.27 (d, *J*_P,6_=6.5 Hz, OCH_2_), 62.80 (C‐4), 62.46 (C‐5), 45.47 (*C*H_2_N), 31.87 (*C*H_2_), 30.66 (d, *J*_P,C_=7.6 Hz, OCH_2_ *C*H_2_), 29.36, 29.32, 25.76, 22.68 (4×CH_2_,), 17.62, 17.54, 17.43, 17.36, 17.28, 17.24, 17.21 (8×CH_3_, TIPDS), 14.10 (CH_3_), 13.90, 13.87, 13.67, 13.25 (4×CH, TIPDS), 8.49 ppm (3×*C*H_3_CH_2_N); ^31^P NMR (243 MHz, CDCl_3_): *δ*=−1.82 ppm. Further purification was achieved by HPLC separation (chloroform/MeOH 9:1+1 % Et_3_N), which afforded a mixture of **8** and **7** (1.2:1, 1.5 mg), as well as compound **7** (16.9 mg, containing 3 % of **8**) as a yellow oil. *R*_f_=0.62 (CHCl_3_/MeOH/25 % aq. NH_3_ 16:4:0.5); [*α*]20D =+44.2 (*c*=1.1 in CHCl_3_); ^1^H NMR for **7** (600 MHz, CDCl_3_): *δ*=5.56 (dd, *J*_2,1_=3.5, *J*_P,1_=6.5 Hz, 1 H; H‐1), 4.32 (dd, *J*_4,3_=4.0, *J*_2,3_=9.2 Hz, 1 H; H‐3), 4.22 (dd, *J*_4,5a_=1.6, *J*_5b,5a_=12.5 Hz, 1 H; H‐5a), 4.03 (ddd, *J*_P,2_=3.2 Hz, 1 H; H‐2), 3.93–3.88 (m, 2 H; OCH_2_), 3.88 (dt, *J*_5a,4_=*J*_5b,4_=1.4 Hz, 1 H; H‐4), 3.56 (dd, 1 H; H‐5b), 3.05 (qd, *J=*3.9, *J=*7.2 Hz, 6 H; 3×C*H_2_*N), 1.59 (quin, 2 H; OCH_2_C*H_2_*), 1.33–1.20 (m, 19 H; 5×CH_2_, 3×CH_3_C*H_2_*N), 1.13–0.90 (m, 28 H; TIPDS), 0.87 ppm (t, *J* ${}_{\mathrm{CH}{}_{2},\mathrm{CH}{}_{3}}$ =7.1 Hz, 3 H; CH_3_); ^13^C NMR (151 MHz, CDCl_3_): *δ*=95.84 (d, *J*_P,1_=6.3 Hz, C‐1), 72.88 (C‐3), 71.91 (d, *J*_P,2_=8.7 Hz, C‐2), 66.07 (d, *J* ${}_{P,\mathrm{OCH}{}_{2}}$ =6.6 Hz, OCH_2_), 62.87 (C‐4), 61.51 (C‐5), 45.36 (CH_2_N), 31.82 (*C*H_2_), 30.67 (d, *J* ${}_{P,\mathrm{OCH}{}_{2}\mathrm{CH}{}_{2}}$ =8.8 Hz, OCH_2_ *C*H_2_), 29.40, 29.29, 25.74, 22.63 (4×*C*H_2_), 17.60, 17.53, 17.33, 17.25, 17.19, 17.11, 17.06 (8×CH_3_, TIPDS), 14.05 (CH_3_), 12.72, 12.34, 12.21 (4×CH, TIPDS), 8.44 ppm (CH_3_ *C*H_2_N); ^31^P NMR (243 MHz, CDCl_3_): *δ*=−2.13 ppm; HRMS (ESI‐TOF): *m*/*z* calcd for C_25_H_52_N_3_O_8_PSi_2_: 608.2958 [*M*−H]^−^; found: 608.2973.

A second experiment with **4** (71.1 mg, 0.122 mmol), octan‐1‐ol (76.8 μL, 0.488 mg), pivaloyl chloride (67.1 μmol) and iodine (46.4 mg) as reagents afforded a mixture of **7** and **8** (9:1, 34.7 mg, 40 %) after two chromatographic purification steps.

**Octyl 1‐*O*‐(4‐amino‐4‐deoxy‐β‐l‐arabinopyranosyl)phosphate, sodium salt** (**9**): Triethylamine trihydrofluoride (22.0 μL, 0.135 mmol) was added at 0 °C to a solution of a mixture of **7** and **8** (9:1, 32.0 mg, 0.045 mmol) in dry THF (112.5 μL) in a screw‐top Teflon flask and the mixture was stirred at RT under Ar for 22 h, after which CaCO_3_ (27 mg) and dry THF (112.5 μL) were added. The suspension was processed as described in General Procedure B to give the crude intermediate product as a colourless oil that was directly used for the next reaction. PMe_3_ (18.5 μL, 0.179 mmol) was added at RT under Ar to a solution of the crude intermediate product in THF/0.1 m NaOH (1:2, 9.9 mL) and the mixture was stirred for 21.5 h. Workup and purification as described for General Procedure C afforded **9** (9.6 mg, 59 %) as a colourless amorphous solid. *R*_f_=0.18 (CHCl_3_/MeOH/25 % aq. NH_3_ 2:2:0.1); [*α*]20D =+37.0 (*c*=0.24 in H_2_O); ^1^H NMR (600 MHz, D_2_O, pD 7.3): *δ*=5.48 (dd, *J*_2,1_=3.6, *J*_P,1_=7.1 Hz, 1 H; H‐1), 4.22 (dd, *J*_4,5a_=2.0, *J*_5b,5a_=13.7 Hz, 1 H; H‐5a), 4.17 (dd, *J*_4,3_=4.6, *J*_2,3_=10.0 Hz, 1 H; H‐3), 3.92–3.87 (m, 2 H; OCH_2_), 3.82 (dd, *J*_4,5b_=2.1 Hz, 1 H; H‐5b), 3.75 (ddd, *J*_P,2_=2.9 Hz, 1 H; H‐2), 3.72 (br dt, 1 H; H‐4), 1.62 (quin, *J=*7.0 Hz, 2 H; OCH_2_C*H_2_*), 1.37–1.23 (m, 10 H; 5×CH_2_), 0.84 ppm (t, *J=*7.0 Hz, 3 H; CH_3_); ^13^C NMR (151 MHz, D_2_O): *δ*=95.74 (d, *J*_P,1_=6.2 Hz, C‐1), 68.69 (d, *J*_P,2_=7.9 Hz, C‐2), 67.58 (d, *J=*5.9 Hz, O*C*H_2_), 66.07 (C‐3), 59.75 (C‐5), 52.49 (C‐4), 31.89 (*C*H_2_), 30.64 (d, *J=*6.8 Hz, OCH_2_ *C*H_2_), 29.18 (2×CH_2_), 25.70, 22.81 (2×CH_2_), 14.20 ppm (*C*H_3_); ^31^P NMR (243 MHz, D_2_O): *δ*=−0.96 (d, *J*_1,P_=5.9 Hz); HRMS (ESI‐TOF): *m*/*z* calcd for C_13_H_28_NO_7_P: 340.1531 [*M*−H]^−^; found: 340.1535.

**Octyl 1‐*O*‐[4‐azido‐4‐deoxy‐2,3‐*O*‐(tetraisopropyldisiloxane‐1,3‐diyl)‐α‐l‐arabinopyranosyl]phosphate, triethylammonium salt** (**10**): *H*‐Phosphonate **5** (60.4 mg, 0.104 mmol) was co‐evaporated with dry toluene (4×10 mL) and dried under high vacuum. Octan‐1‐ol (65.3 μL, 0.415 mmol) was pre‐dried over molecular sieves (4 Å) and added at RT under Ar to a solution of **5** in dry 2,6‐lutidine (2.5 mL). The mixture was stirred for 10 min and treated with pivaloyl chloride (57.4 μL, 0.466 mmol) for 22.5 h at RT. The reaction mixture was cooled to 0 °C, a solution (2 %) of iodine (39.5 mg, 0.155 mmol) in 2,6‐lutidine/water (50:1, 1.10 mL) was added dropwise, and stirring was continued at 0 °C for 1 h. Workup and purification as described in General Procedure A afforded **10** (27.1 mg, 37 %) as a yellow oil. *R*_f_=0.48 (CHCl_3_/MeOH/25 % aq. NH_3_ 16:4:0.5); [*α*]20D =−24.3 (*c*=1.3 in CHCl_3_); ^1^H NMR (600 MHz, CDCl_3_): *δ*=4.90 (dd, *J*_2,1_=7.3, *J*_P,1_=7.3 Hz, 1 H; H‐1), 3.93 (dd, *J*_4,3_=4.2, *J*_2,3_=8.9 Hz, 1 H; H‐3), 3.93–3.84 (m, 2 H; OCH_2_), 3.81 (dd, *J*_4,5a_=1.8, *J*_5b,5a_=13.0 Hz, 1 H; H‐5a), 3.79 (dd, 1 H; H‐2), 3.74 (dt, *J*_5a,4_=1.8 Hz, 1 H; H‐4), 3.60 (dd, 1 H; H‐5b), 3.04 (q, *J=*6.7 Hz, 6 H; 3×CH_2_N), 1.58 (quin, *J=*7.2 Hz, 2 H; OCH_2_C*H_2_*), 1.32–1.23 (m, 19 H; 5×CH_2_, 3×CH_3_CH_2_N), 1.12–0.90 (m, 28 H; TIPDS), 0.86 ppm (t, *J* ${}_{\mathrm{CH}{}_{2},\mathrm{CH}{}_{3}}$ =7.2 Hz, 3 H; CH_3_); ^13^C NMR (151 MHz, CDCl_3_): *δ*=98.85 (d, *J*_P,1_=5.8 Hz, C‐1), 77.25 (C‐3), 74.75 (d, *J*_P,2_=9.6 Hz, C‐2), 65.95 (d, *J* ${}_{P,\mathrm{OCH}{}_{2}}$ =5.9 Hz, O*C*H_2_), 64.44 (C‐5), 61.96 (C‐4), 45.34 (*C*H_2_N), 31.81 (*C*H_2_), 30.67 (d, *J* ${}_{P,\mathrm{OCH}{}_{2}\mathrm{CH}{}_{2}}$ =7.7 Hz, OCH_2_ *C*H_2_), 29.40, 29.28, 25.70, 22.62 (4×CH_2_), 17.56, 17.33, 17.23, 17.18, 17.17, 17.16, 17.00, 16.99 (8×CH_3_, TIPDS), 14.04 (CH_3_), 12.78, 12.69, 12.12, 11.96 (4×CH, TIPDS), 8.42 ppm (3×*C*H_3_CH_2_N); ^31^P NMR (243 MHz, CDCl_3_): *δ*=−2.43 ppm (d, *J*_1,P_=5.9 Hz); HRMS (ESI‐TOF): *m*/*z* calcd for C_25_H_52_N_3_O_8_PSi_2_: 608.2958 [*M*−H]^−^; found: 608.2956.

**Octyl 1‐*O*‐(4‐amino‐4‐deoxy‐α‐l‐arabinopyranosyl) phosphate, sodium salt** (**11**): Triethylamine trihydrofluoride (17.6 μL, 0.108 mmol) was added at 0 °C to a solution of **10** (25.6 mg, 0.036 mmol) in dry THF (90 μL) in a screw‐top Teflon flask and the mixture was stirred at RT under Ar for 20 h. CaCO_3_ (22 mg) and dry THF (90 μL) were then added. The suspension was stirred at RT for 1 h and centrifuged at 1972 *g* for 15 min. The liquid phase was recovered and lyophilised overnight, to give the crude intermediate product as a colourless oil that was directly used for the next reaction. P(Me)_3_ (15.0 μL, 0.145 mmol) was added at RT under Ar to a solution of the crude intermediate product in THF/0.1 m NaOH (1:2, 8 mL). The solution was stirred for 4 h, diluted with water (2 mL) and washed with chloroform (5×2 mL). The combined organic phases were re‐extracted with water (2×20 mL) and processed as described in General Procedure C to afford **11** (4.7 mg, 36 %) as a colourless solid. *R*_f_=0.14 (CHCl_3_/MeOH/25 % aq. NH_3_ 2:2:0.1); [α20D =+2.2 (*c*=0.31 in D_2_O); ^1^H NMR (600 MHz, D_2_O, pD 7.0): *δ*=4.85 (dd, *J*_2,1_=*J*_P,1_=7.1 Hz, 1 H; H‐1), 3.96 (dd, *J*_4,5a_=3.5, *J*_5b,5a_=13.0 Hz, 1 H; H‐5a), 3.91–3.88 (m, 3 H; H‐3, OCH_2_), 3.75 (dd, *J*_4,5b_=2.3, Hz, 1 H; H‐5b), 3.59 (dd, *J*_2,3_=8.6 Hz, 1 H; H‐2), 3.38 (br, 1 H; H‐4), 1.61 (quin, *J=*7.0 Hz, 2 H; OCH_2_C*H_2_*), 1.36–1.24 (m, 10 H; 5×CH_2_), 0.85 ppm (t, *J* ${}_{\mathrm{CH}{}_{2},\mathrm{CH}{}_{3}}$ =7.0 Hz, 3 H; CH_3_); ^13^C NMR (151 MHz, D_2_O): *δ*=98.78 (d, *J*_P,1_=5.9 Hz, C‐1), 71.40 (d, *J*_P,2_=8.3 Hz, C‐2), 70.84 (C‐3), 67.63 (d, *J* ${}_{P,\mathrm{OCH}{}_{2}}$ =5.8 Hz, O*C*H_2_), 64.24 (C‐5), 50.75 (C‐4), 31.90 (*C*H_2_), 30.59 (d, *J* ${}_{P,\mathrm{OCH}{}_{2}\mathrm{CH}{}_{2}}$ =6.9 Hz, OCH_2_ *C*H_2_), 29.19 (2×CH_2_), 25.70, 22.82 (2×CH_2_), 14.21 ppm (*C*H_3_); ^31^P NMR (243 MHz, D_2_O): *δ*=−1.07 (d, *J*_1,P_=5.9 Hz); HRMS (ESI‐TOF): *m*/*z* calcd for C_13_H_28_NO_7_P: 340.1531 [*M*−H]^−^; found: 340.1539.

**Pivaloyl 1‐*O*‐[4‐azido‐4‐deoxy‐2,3‐*O*‐(tetraisopropyldisiloxane‐1,3‐diyl)‐β‐l‐arabinopyranosyl]phosphonate, triethylammonium salt (12)**: *H*‐Phosphonate **4** (36.6 mg, 0.063 mmol) was co‐evaporated with dry toluene (4×10 mL) and dried under high vacuum overnight. Dry 2,6‐lutidine (1.5 mL) was added, the reaction mixture was stirred for 10 min at RT under Ar, and 50 μL of the reaction mixture was transferred into an NMR tube with CDCl_3_ (0.4 mL) to allow monitoring of the reaction. Pivaloyl chloride (34.8 μL, 0.283 mmol) was then added dropwise at RT and the reaction mixture was stirred. After 2.25 h, a second aliquot (50 μL) of the reaction mixture was transferred into an NMR tube and diluted with CDCl_3_ (0.4 mL). The NMR spectrum showed total conversion of the starting material into an intermediate product. After 2.25 h, the solution was cooled to 0 °C, a solution (2 %) of iodine (23.9 mg, 0.094 mmol) in 2,6‐lutidine/water (50:1, 0.64 mL) was added dropwise, and the mixture was stirred at 0 °C for 1 h. At this point, an NMR sample was withdrawn from the reaction mixture; it showed completion of the reaction. The solution was diluted with chloroform (40 mL) followed by addition of a mixture of 7 % aq. Na_2_S_2_O_3_/2 m TEAB buffer (1:1, 16 mL). The aqueous phase was extracted with chloroform (2×40 mL) and the combined organic phases were filtered over cotton, concentrated and co‐evaporated with toluene (3 mL) under reduced pressure. The product was purified by flash chromatography on silica gel (chloroform containing 1 % Et_3_N→chloroform/MeOH 9:1+1 % Et_3_N). Product‐containing fractions were pooled and concentrated. The residue was dissolved in chloroform (7 mL) and washed with TEAB buffer (0.25 m, 4 mL). The organic phase was dried over cotton and concentrated to give a fraction of pure **12** (10.7 mg). ^1^H NMR (600 MHz, CDCl_3_): *δ*=5.68 (dd, *J*_2,1_=3.7, *J*_P,1_=5.8 Hz, 1 H; H‐1), 4.29 (dd, *J*_4,3_=4.1, *J*_2,3_=9.2 Hz, 1 H; H‐3), 4.23 (dd, *J*_4,5a_=1.8, *J*_5b,5a_=12.5 Hz, 1 H; H‐5a), 4.04 (ddd, *J*_P,2_=2.2 Hz, 1 H; H‐2), 3.87 (dt, *J*_5a,4_=1.8 Hz, 1 H; H‐4), 3.56 (dd, 1 H; H‐5b), 3.08 (qd, *J=*4.7, *J=*7.3 Hz, 6 H; 3×C*H_2_*N), 1.34 (t, 9 H; 3×CH_3_C*H_2_*N), 1.25 (s, 9 H; 3×CH_3_), 1.10–0.88 ppm (m, 28 H; TIPDS); ^13^C NMR (151 MHz, CDCl_3_): *δ*=218.73 (C=O), 96.19 (C‐1), 72.89 (C‐3), 71.45 (d, *J*_P,2_=7.7 Hz, C‐2), 62.61 (C‐4), 62.07 (C‐5), 45.73 (3×CH_2_N), 45.72 [*C*(CH_3_)_3_], 25.42 [C(*C*H_3_)_3_], 17.54, 17.53, 17.30, 17.26, 17.23, 17.20, 17.07, 17.03 (8×CH_3_, TIPDS), 12.74, 12.69, 12.32, 12.19 (4×CH, TIPDS), 8.55 ppm ( 3×CH_3_ *C*H_2_N); ^31^P NMR (243 MHz, CDCl_3_): *δ*=−4.91 ppm; HRMS (ESI‐TOF): *m*/*z* calcd for C_22_H_44_N_3_O_8_PSi_2_: 566.2477 [*M*+H^+^]^+^; found: 566.2489.

**(2*E*)‐3,7‐Dimethylocta‐2,6‐dien‐1‐yl 1‐*O*‐[4‐azido‐4‐deoxy‐2,3‐*O*‐(tetraisopropyldisiloxane‐1,3‐diyl)‐β‐l‐arabinopyranosyl]phosphate, triethylammonium salt (13)**: A solution of *H*‐phosphonate **4** (50.1 mg, 0.086 mmol) in dry 2,6‐lutidine (2.1 mL) was treated with (2*E*)‐3,7‐dimethylocta‐2,6‐dien‐1‐ol (60.3 μL, 0.344 mmol) followed by pivaloyl chloride (47.6 μL, 0.387 mmol) for 46 h at RT. The solution was then oxidized with a solution (2 %) of iodine (32.7 mg, 0.129 mmol) in 2,6‐lutidine/water (50:1, 0.88 mL) for 1 h at 0 °C. Workup and purification as described in General Procedure A furnished **13** (25 mg, containing 5 % of **12**) as a yellow oil (37 %). Final purification by HPLC (chloroform/MeOH 9:1+1 % Et_3_N) gave pure **13** (14.9 mg, 24 %) as colourless amorphous solid. *R*_f_=0.65 (CHCl_3_/MeOH/25 % aq. NH_3_ 16:4:0.5); [*α*]20D =+16.7 (*c*=0.98 in CHCl_3_); ^1^H NMR (600 MHz, CDCl_3_): *δ*=5.60 (dd, *J*_2′,1′_=3.5, *J*_P,1′_=6.2 Hz, 1 H; H‐1′), 5.36 (t, *J*_9,2_=1.3, *J*_1,2_=6.8 Hz, 1 H; H‐2), 5.08 (m, *J*_10,6_ ≈*J*_8,6_=1.4, *J*_5,6_=6.8 Hz, 1 H; H‐6), 4.50 (m, 2 H; H‐1a, H‐1b), 4.29 (dd, *J*_4′,3′_=4.0, *J*_2′,3′_=9.2 Hz, 1 H; H‐3′), 4.16 (dd, *J*_4′,5′a_=1.5, *J*_5′b,5′a_=12.5 Hz, 1 H; H‐5′a), 4.06 (ddd, *J*_P,2′_=3.2 Hz, 1 H; H‐2′), 3.86 (dt, *J*_5′b,4′_ ≈*J*_5′a,4′_=1.5 Hz, 1 H; H‐4′), 3.61 (dd, 1 H; H‐5′b), 3.04–2.96 (m, *J=*7.3 Hz, 6 H; 3×CH_2_N), 2.07 (dt, *J*_4,5_=7.1 Hz, 2 H; H‐5), 2.01–1.99 (m, 2 H; H‐4), 1.68 (s, 3 H; H‐8), 1.64 (s, 3 H; H‐9), 1.59 (s, 3 H; H‐10), 1.30 (t, 9 H; 3×C*H_3_*CH_2_N), 1.12–0.90 ppm (m, 28 H; TIPDS); ^13^C NMR (151 MHz, CDCl_3_): *δ*=140.35 (HMBC‐signal, C‐3), 131.65 (C‐7), 123.91 (C‐6), 120.30 (C‐2), 96.52 (d, *J*_P,1′_=7.3 Hz, C‐1′), 72.80 (C‐3′), 71.68 (d, *J*_P,2′_=8.8 Hz, C‐2′), 63.32 (d, *J*_P,1_=5.1 Hz, C‐1), 62.63 (C‐4′), 61.84 (C‐5′), 45.50 (*C*H_2_N), 39.54 (C‐4), 26.40 (C‐5), 25.65 (C‐8), 17.65 (C‐10), 17.64, 17.54, 17.38, 17.26, 17.24, 17.20, 17.11, 17.06 (8×CH_3_, TIPDS),16.35 (C‐9), 12.75 (2×CH, TIPDS), 12.29, 12.21 (2×CH, TIPDS), 8.48 ppm (3×*C*H_3_CH_2_N); ^31^P NMR (243 MHz, CDCl_3_): *δ*=−1.91 ppm; HRMS (ESI‐TOF): *m*/*z* calcd for C_27_H_52_N_3_O_8_PSi_2_: 632.2958 [*M*−H]^−^; found: 632.2958.

In a second experiment a solution of **4** (66.2 mg, 0.114 mmol), (2*E*)‐3,7‐dimethylocta‐2,6‐dien‐1‐ol (79.7 μL, 0.454 mg) and pivaloyl chloride (62.9 μL) was stirred for 20.5 h at RT followed by oxidation with iodine (43.2 mg) in 2,6‐lutidine/water (50:1, 1.16 mL) for 1 h at 0 °C; this afforded a mixture (1:0.33:0.41) of **13**, **12** and **14** (41.6 mg). After two additional chromatographic purification steps a fraction of pure **14** (2.6 mg) was obtained as a syrup. ^1^H NMR (600 MHz, CDCl_3_): *δ*=5.67 (dd, *J*_2,1_=3.5, *J*_P,1_=6.9 Hz, 1 H; H‐1), 4.31 (dd, *J*_4,3_=4.0, *J*_2,3_=9.2 Hz, 1 H; H‐3), 4.18 (dd, *J*_4,5a_=1.6, *J*_5b,5a_=12.5 Hz, 1 H; H‐5a), 4.07 (ddd, *J*_P,2_=3.2 Hz, 1 H; H‐2), 3.86 (dt, *J*_5a,4_=*J*_5b,4_=1.8 Hz, 1 H; H‐4), 3.60 (dd, 1 H; H‐5b), 3.10 (dq, *J=*4.6, *J=*7.3 Hz, 6 H; 3×C*H_2_*N), 1.34 (t, 9 H; 3×C*H_3_*CH_2_N), 1.20 (s, 9 H; 3×CH_3_), 1.10–1.01 ppm (m, 28 H; TIPDS); ^13^C NMR (151 MHz, CDCl_3_): *δ*=174.80 (HMBC signal, C=O), 96.4 (HMBC signal, C‐1), 72.80 (C‐3), 71.67 (d, *J*_P,2_=8.6 Hz, C‐2), 62.73 (C‐4), 61.70 (C‐5), 45.72 (CH_2_N), 39.34 [*C*(CH_3_)_3_], 26.89 [C(*C*H_3_)_3_], 17.59, 17.52, 17.35, 17.27, 17.16, 17.11 (8×CH_3_, TIPDS), 12.84, 12.68, 12.24, 12.15 (4×CH, TIPDS), 8.50 ppm (CH_3_ *C*H_2_N); ^31^P NMR (243 MHz, CDCl_3_): *δ*=−8.73 ppm; HRMS (ESI‐TOF): *m*/*z* calcd for C_22_H_44_N_3_O_9_PSi_2_: 582.2426 [*M*+H^+^]^+^; found: 582.2436.

**(2*Z*)‐3,7‐Dimethylocta‐2,6‐dien‐1‐yl 1‐*O*‐[4‐azido‐4‐deoxy‐2,3‐*O*‐(tetraisopropyldisiloxane‐1,3‐diyl)‐β‐l‐arabinopyranosyl] phosphate, triethylammonium salt (15)**: A solution of *H*‐phosphonate **4** (166.6 mg, 0.286 mmol) in dry 2,6‐lutidine (2.1 mL) was treated with (2*Z*)‐3,7‐dimethylocta‐2,6‐dien‐1‐ol (251.7 μL, 1.429 mmol) followed by pivaloyl chloride (87.9 μL, 0.715 mmol) for 23 h at RT. The solution was then oxidized with a solution (2 %) of iodine (108.8 mg, 0.429 mmol) in 2,6‐lutidine/water (50:1, 2.9 mL) for 1 h at 0 °C. Workup and purification as described in General Procedure A furnished a fraction of **15** (79 mg, containing 4 % of **12** and **14**) and a second fraction of **15** (12.6 mg, containing 5 % of **12**) as a yellow oil. [*α*]20D =+32.8 (*c*=0.7 in CHCl_3_); *R*_f_=0.65 (16:4:0.5 CHCl_3_/MeOH/25 % aq. NH_3_): ^1^H NMR (600 MHz, CDCl_3_): *δ*=5.59 (dd, *J*_1′,2′_=3.6, *J*_P,1′_=6.3 Hz, 1 H; H‐1′), 5.36 (qt, *J*_2,9_=1.1, *J*_2,9_=6.9 Hz, 1 H; H‐2), 5.08–5.05 (m, 1 H; H‐6), 4.47 (dd, *J*_P,1_=6.9 Hz, 2 H; H‐1a, H‐1b), 4.28 (dd, *J*_4′,3′_=3.9, *J*_2′,3′_=9.2 Hz, 1 H; H‐3′), 4.16 (dd, *J*_4′,5′a_=1.4, *J*_5′b,5′a_=12.7 Hz, 1 H; H‐5′a), 4.05 (ddd, *J*_P,2′_=3.3 Hz, 1 H; H‐2′), 3.85 (dt, *J*_5′b,4′_ ≈*J*_5′a,4′_=1.7 Hz, 1 H; H‐4′), 3.60 (dd, 1 H; H‐5′b), 3.07 (m, 6 H; 3×CH_2_N), 2.08–2.01 (m, 4 H; H‐4, H‐5), 1.71 (d, *J*_2,9_=1.2 Hz, 3 H; H‐9), 1.67 (s, 3 H; H‐8), 1.59 (s, 3 H; H‐10), 1.29 (t, *J=*7.3 Hz, 9 H; 3×C*H_3_*CH_2_N), 1.12–0.89 ppm (m, 28 H; TIPDS); ^13^C NMR (151 MHz, CDCl_3_): *δ*=140.36 (HMBC signal, C‐3), 131.85 (C‐7), 123.79 (C‐6), 121.44 (d, *J*_P,2_=5.1 Hz C‐2), 96.51 (d, *J*_P,1′_=5.0 Hz, C‐1′), 72.80 (C‐3′), 71.65 (d, *J*_P,2′_=8.5 Hz, C‐2′), 62.99 (d, *J*_P,1_=4.9 Hz, C‐1), 62.63 (C‐4′), 61.81 (C‐5′), 45.51 (3×*C*H_2_N), 32.11 (C‐4), 26.70 (C‐5), 25.64 (C‐8), 23.35 (C‐9), 17.65 (C‐10), 17.60, 17.53, 17.24, 17.19, 17.18, 17.10, 17.05 (8×CH_3_, TIPDS), 12.74, 12.734, 12.27, 12.20 (4×CH, TIPDS), 8.47 ppm (3×*C*H_3_CH_2_N); ^31^P NMR (243 MHz, CDCl_3_): *δ*=−1.92 ppm; HRMS (ESI‐TOF): *m*/*z* calcd for C_27_H_52_N_3_O_8_PSi_2_+COOH^−^: 678.3013 [*M*+COOH]^−^; found: 678.3064.

**(2*E*)‐3,7‐Dimethylocta‐2,6‐dien‐1‐yl 1‐*O*‐(4‐amino‐4‐deoxy‐β‐l‐arabinopyranosyl)phosphate, sodium salt** (**16**): Triethylamine trihydrofluoride (36 μL, 0.221 mmol) was added at 0 °C to a solution of **13** (58.7 mg, containing 25 % of **12** and **14**) in dry THF (200 μL) in a screw‐top Teflon flask and the mixture was stirred at RT under Ar for 22 h. CaCO_3_ (44 mg) and dry THF (200 μL) were then added and processed as described in General Procedure B. P(Me)_3_ (30.4 μL, 0.295 mmol) was added at RT under Ar to a solution of the crude intermediate product in THF/0.1 m NaOH (1:2, 16 mL) and the mixture was stirred for 4 h. Extraction and purification as described afforded **16** (6.7 mg, 30 %, based on **13**) as a colourless amorphous solid. *R*_f_=0.21 (CHCl_3_/MeOH/25 % aq. NH_3_ 2:2:0.1); [*α*]20D =+64.2 (*c*=0.4, D_2_O); ^1^H NMR (600 MHz, D_2_O, pD 7.5): *δ*=5.47 (dd, *J*_2′,1′_=3.5, *J*_P,1′_=7.1 Hz, 1 H; H‐1′), 5.40 (dtt, *J*_9,2_=1.2, *J*_1,2_=6.9 Hz, 1 H; H‐2), 5.18 (m, *J*_6,10_ ≈*J*_6,8_=1.3, *J*_6,5_=7.0 Hz, 1 H; H‐6), 4.47–4.41 (m, 2 H; H‐1a, H‐1b), 4.19 (dd, *J*_4′,5′a_=2.2, *J*_5′b,5′a_=13.4 Hz, 1 H; H‐5′a), 4.13 (dd, *J*_4′,3′_=4.6, *J*_2′,3′_=9.9 Hz, 1 H; H‐3′), 3.78 (dd, *J*_4′,5′b_=2.4 Hz, 1 H; H‐5′b), 3.75 (ddd, *J*_P,2′_=2.8 Hz, 1 H; H‐2′), 3.71 (dt, 1 H; H‐4′), 2.14 (m, 2 H; H‐5), 2.07 (m, 2 H; H‐4), 1.69 (d, 3 H; H‐9), 1.67 (d, 3 H; H‐8), 1.61 ppm (s, 3 H; H‐10); ^13^C NMR (151 MHz, D_2_O): *δ*=143.82 (C‐3), 134.58 (C‐7), 124.86 (C‐6), 120.23 (d, *J*_P,2_=7.8 Hz, C‐2), 95.89 (d, *J*_P,1′_=6.8 Hz, C‐1′), 68.71 (d, *J*_P,2′_=8.8 Hz, C‐2′), 66.71 (C‐3′), 63.77 (d, *J*_P,1_=5.4 Hz, C‐1), 60.61 (C‐5′), 52.24 (C‐4′), 39.53 (C‐4), 26.37 (C‐5), 25.61 (C‐8), 17.76 (C‐10), 16.37 ppm (C‐9); ^31^P NMR (243 MHz, D_2_O): *δ*=−0.98 ppm (d, *J*_1′,P_=5.9 Hz); HRMS (ESI‐TOF): *m*/*z* calcd for C_15_H_28_NO_7_P: 364.1531 [*M*−H]^−^; found: 364.1535.

(**2*Z*)‐3,7‐Dimethylocta‐2,6‐dien‐1‐yl 1‐*O*‐(4‐amino‐4‐deoxy‐β‐l‐arabinopyranosyl)phosphate, sodium salt** (**17**): Triethylamine trihydrofluoride (40.9 μL, 0.396 mmol) was added at 0 °C to a solution of **15** (79 mg, containing 4 % of **12** and **14**) in dry THF (250 μL) in a screw‐top Teflon flask and the mixture was stirred at RT under Ar for 19 h. CaCO_3_ (60 mg) and dry THF (200 μL) were then added and processed as described in General Procedure **B**. P(Me)_3_ (30.4 μL, 0.295 mmol) was added at RT under Ar to a solution of the crude intermediate product in THF/0.1 m NaOH (1:2, 22 mL) and the mixture was stirred for 4 h. Extraction and purification as described afforded **17** (0.94 mg, 0.002 mmol) in 4 % yield (over two steps) as a white solid. *R*_f_=0.21 (CHCl_3_/MeOH/25 % aq. NH_3_ 2:2:0.1); [*α*]20D =+76.0 (*c*=0.05 in D_2_O); ^1^H NMR (600 MHz, D_2_O, pD 7.1): *δ*=5.44 (dd, *J*_2′,1′_=3.5, *J*_P,1′_=7.0 Hz, 1 H; H‐1′), 5.42 (tq, *J*_2,9_=1.0, *J*_2,1_=7.1 Hz, 1 H; H‐2), 5.17 (m, *J*_8,6_ ≈*J*_10,6_=1.4, *J*_6.5_=6.4 Hz, 1 H; H‐6), 4.41 (dd, *J*_P,1_=6.9 Hz, 2 H; H‐1a. H‐1b), 4.11 (dd, *J*_4′,5′a_=2.3, *J*_5′b,5′a_=12.8 Hz, 1 H; H‐5′a), 4.02 (dd, *J*_4′,3′_=4.4, *J*_2′,3′_=9.8 Hz, 1 H; H‐3′), 3.78 (ddd, *J*_P,2′_=2.4 Hz, 1 H; H‐2′), 3.67 (dd, *J*_4′,5′b_=2.5 Hz, 1 H; H‐5′b), 3.37–3.35 (m, 1 H; H‐4′), 2.16–2.11 (m, 4 H; H‐4, H‐5), 1.76 (d, 3 H; H‐9), 1.68 (s, 3 H; H‐8), 1.61 ppm (s, 3 H; H‐10); ^13^C NMR (151 MHz, D_2_O): *δ*=144.01 (C‐3), 134.81 (C‐7), 124.61 (C‐6), 121.12 (d, *J*_P,2_=7.7 Hz, C‐2), 95.96 (d, *J*_P,1′_=6.6 Hz, C‐1′), 68.75 (d, *J*_P,2′_=7.7 Hz, C‐2′), 67.11 (C‐3′), 63.53 (d, *J*_P,1_=5.5 Hz, C‐1), 61.12 (C‐5′), 52.06 (C‐4′), 32.09 (C‐4), 26.75 (C‐5), 25.66 (C‐8), 23.43 (C‐9), 17.76 ppm (C‐10); ^31^P NMR (243 MHz, D_2_O): *δ*=0.45 ppm (d, *J*_1,P_=5.85 Hz); HRMS (ESI‐TOF): *m*/*z* calcd for C_15_H_28_NO_7_P: 366.1676 [*M*+H]^+^; found: 366.1676.

**Eicosyl 1‐*O*‐[4‐azido‐4‐deoxy‐2,3‐*O*‐(tetraisopropyldisiloxane‐1,3‐diyl)‐α‐l‐arabinopyranosyl]phosphate triethylammonium salt (19)**: Eicosan‐1‐ol (**18**, 67.2 mg, 0.225 mmol) was dried over molecular sieves and added to a solution of pre‐dried **5** (109.3 mg, 0.188 mmol) in dry dichloromethane (3.7 mL) and dry 2,6‐lutidine (108.9 μL, 0.938 mmol), followed by addition of PyTP (118.3 mg, 0.281 mmol) at RT under Ar. The solution was stirred for 5 h, after which *N,O*‐bis(trimethylsilyl)acetamide (68.6 μL, 0.281 mmol) and triethylamine (52.1 μL, 0.374 mmol) were added. Stirring was continued for 2 h at RT, (1*S*)‐(+)‐(Camphorsulfonyl)oxaziridine (64.3 mg, 0.281 mmol) was added, and the mixture was stirred for 1 h at RT. It was then diluted with chloroform (100 mL) and quenched by addition of a mixture of 7 % aq. Na_2_S_2_O_3_/2 m TEAB buffer (1:1, 100 mL). The aqueous phase was extracted with chloroform (2×50 mL) and the combined organic phases were filtered over cotton, concentrated and co‐evaporated with toluene (10 mL) under reduced pressure. The product was purified by flash column chromatography on silica gel (CHCl_3_+1 % Et_3_N→CHCl_3_/MeOH 9:1+1 % Et_3_N). The residue was dissolved in chloroform (14 mL) and washed with TEAB buffer (0.25 m, 8 mL). The organic phase was dried over cotton and concentrated to afford **19** (17.1 mg, 11 %) as a yellow oil. *R*_f_=0.67 (CHCl_3_/MeOH/25 % aq. NH_3_ 16:4:0.5); [α]20D =−12.2 (*c*=0.7 in CHCl_3_); ^1^H NMR (600 MHz, CDCl_3_): *δ*=4.92 (dd, *J*_2,1_=7.3, *J*_P,1_=7.3 Hz, 1 H; H‐1), 3.94 (dd, *J*_4,3_=4.1, *J*_2,3_=8.8 Hz, 1 H; H‐3), 3.92–3.83 (m, 2 H; OCH_2_), 3.81 (dd, *J*_4,5a_=1.4, *J*_5b,5a_=13.0 Hz, 1 H; H‐5a), 3.80 (dd, 1 H; H‐2), 3.75 (dt, *J*_5a,4_≈*J*_5b,4_=1.8 Hz, 1 H; H‐4), 3.60 (dd, 1 H; H‐5b), 3.03 (q, *J=*7.3 Hz, 6 H; 3×CH_2_N), 1.58 (quin, *J=*7.3 Hz, 2 H; OCH_2_C*H_2_*), 1.31 (t, 9 H; 3×C*H_3_*CH_2_N), 1.29–1.25 (m, 34 H; 17×CH_2_), 1.13–0.99 (m, 28 H; TIPDS), 0.88 ppm (t, *J=*6.9 Hz, 3 H; CH_3_); ^13^C NMR (151 MHz, CDCl_3_): *δ*=98.90 (d, *J*_P,1_=5.7 Hz, C‐1), 77.32 (C‐3), 74.89 (d, *J*_P,2_=9.1 Hz, C‐2), 65.68 (d, *J* ${}_{P,\mathrm{OCH}{}_{2}}$ =5.8 Hz, O*C*H_2_), 64.39 (C‐5), 62.05 (C‐4), 45.29 (3×*C*H_2_N), 31.92 (CH_2_), 30.79 (d, *J* ${}_{P,\mathrm{OCH}{}_{2}\mathrm{CH}{}_{2}}$ =8.5 Hz, OCH_2_ *C*H_2_), 29.70, 29.65, 29.52, 29.35, 25.80, 22.68, (16×CH_2_), 17.59, 17.38, 17.28, 17.21, 17.04, 17.03 (8×CH_3_, TIPDS), 14.09 (CH_3_), 12.80, 12.72, 12.15, 11.94 (4×CH, TIPDS), 8.46 ppm (3×*C*H_3_CH_2_N); ^31^P NMR (243 MHz, CDCl_3_): *δ*=−1.74 ppm (d, *J*_1,P_=6.2 Hz); HRMS (ESI‐TOF): *m*/*z* calcd for C_37_H_76_N_3_O_8_PSi_2_: 776.4836 [*M*−H]^−^; found: 776.4854.

**Eicosyl 1‐*O*‐(4‐amino‐4‐deoxy‐α‐l‐arabinopyranosyl)phosphate sodium salt** (**20**): Triethylamine trihydrofluoride (9.5 μL, 0.058 mmol) was added at 0 °C to a solution of **19** (17.1 mg, 0.019 mmol) in dry THF (50 μL) in a screw‐top Teflon flask and the mixture was stirred at RT under Ar for 15.5 h. CaCO_3_ (12 mg) and dry THF (50 μL) were then added. The suspension was stirred at RT for 1 h and then centrifuged at 1972 *g* for 15 min. The liquid phase was recovered and lyophilised overnight, to give the crude intermediate product as a colourless oil that was directly used for the next reaction. Dithiothreitol (DTT, 11.6 mg, 0.075 mmol) was added at RT under Ar to a solution of the crude intermediate product in water/diisopropylamine (3:1, 0.8 mL) and the mixture was stirred for 3 h. The pink reaction mixture was then diluted with water (2.5 mL) and washed with diethyl ether (5×2.5 mL). The combined organic phases were re‐extracted with water (2×25 mL) and stripped with Ar until the solution became clear. The aqueous phase was frozen and lyophilised. The product was purified by gel column chromatography [ZIC‐HILIC‐HPLC column (5 μm) eluted with acetonitrile/water 95:5→40:60 with a flow rate of 3 mL min^−1^]. Lyophilisation of the product fractions afforded **20** (1.41 mg, 15 %) as a white solid. *R*_f_=0.13 (CHCl_3_/MeOH 3:1); [*α*]20D =+14.2 (*c*=0.14 in MeOH); ^1^H NMR (600 MHz, MeOD, pD 5.6): *δ*=4.80 (dd, *J*_2,1_=*J*_P,1_=7.3 Hz, 1 H; H‐1), 3.93 (dd, *J*_4,5a_=3.2, *J*_5b,5a_=12.8 Hz, 1 H; H‐5a), 3.91–3.87 (m, 2 H; OCH_2_), 3.79 (dd, *J*_4,3_=3.9, *J*_2,3_=8.7 Hz, 1 H; H‐3), 3.76 (dt, *J*_5a,4_ ≈*J*_5b,4_=2.7 Hz, 1 H; H‐4), 3.63 (dd, 1 H; H‐5b), 3.57 (dd, 1 H; H‐2), 1.61 (quin, *J=*7.1 Hz, 2 H; OCH_2_C*H_2_*), 1.43–1.29 (m, 34 H; 17×CH_2_), 0.90 ppm (t, *J=*7.1 Hz, 3 H; CH_3_); ^13^C NMR (151 MHz, MeOD): *δ*=100.86 (d, *J*_P,1_=6.6 Hz, C‐1), 74.95 (C‐3), 73.96 (d, *J*_P,2_=7.7 Hz, C‐2), 67.70 (d, *J* ${}_{P,\mathrm{OCH}{}_{2}}$ =5.9 Hz, OCH_2_), 65.39 (C‐5), 63.10 (C‐4), 33.92 (CH_2_), 32.61 (d, *J*_P_=7.6 Hz, OCH_2_ *C*H_2_), 31.62, 31.36, 31.32, 27.74, 24.58 (16×CH_2_), 15.27 ppm (CH_3_); ^31^P NMR (243 MHz, MeOD): *δ*=−1.13 ppm; HRMS (ESI‐TOF): *m*/*z* calcd for C_25_H_52_NO_7_P: 508.3409 [*M*−H]^−^; found: 508.3425.

**(2*E*)‐3,7‐Dimethylocta‐2,6‐dien‐1‐yl 1‐*O*‐[4‐azido‐4‐deoxy‐2,3‐*O*‐(tetraisopropyldisiloxane‐1,3‐diyl)‐α‐l‐arabinopyranosyl]phosphate, triethylammonium salt (23)**: *H*‐Phosphonate **5** (77.1 mg, 0.132 mmol) was coevaporated with dry toluene (4×10 mL) and dried under high vacuum overnight. (2*E*)‐3,7‐Dimethylocta‐2,6‐dien‐1‐ol (116.1 μL, 0.661 mmol) was pre‐dried over molecular sieves (4 Å), added to a solution of **5** in dry 2,6‐lutidine (1.3 mL), and stirred for 10 min under Ar at RT. Pivaloyl chloride (40.7 μL, 0.331 mmol) was then added dropwise and the solution was stirred for 22 h at RT. A solution (2 %) of iodine (50.4 mg, 0.198 mmol) in 2,6‐lutidine/water 50:1 (1.3 mL) was added dropwise at 0 °C and stirring was continued for 1 h. Processing as described in General Procedure A afforded a fraction of **23** containing 6 % of pivaloyl by‐product **21** (15.5 mg) and a fraction of pure **23** (16.1 mg, 32 % combined yield) as a yellow oil. *R*_f_=0.63 (CHCl_3_/MeOH/25 % aq. NH_3_ 16:4:0.5); [*α*]20D =−18.3 (*c*=0.54 CHCl_3_); ^1^H NMR (600 MHz, CDCl_3_): *δ*=5.35 (tq, *J*_9,2_=1.2, *J*_1,2_=9.9 Hz, 1 H; H‐2), 5.09 (m, *J*_6,8_=*J*_6,10_=1.4, *J*_6,5_=7.2 Hz, 1 H; H‐6), 4.91 (dd, *J*_2′,1′_=*J*_P,1′_=7.4 Hz, 1 H; H‐1′), 4.52–4.44 (m, 2 H; H‐1a, H‐1b), 3.94 (dd, *J*_4′,3′_=4.2, *J*_2′,3′_=8.8 Hz, 1 H; H‐3′), 3.85 (dd, *J*_4′,5′a_=1.2, *J*_5′b,5′a_=13.2 Hz, 1 H; H‐5′a), 3.82 (dd, 1 H; H‐2′), 3.76 (dt, *J*_5′a,4′_ ≈*J*_5′b,4′_=1.8 Hz, 1 H; H‐4′), 3.61 (dd, 1 H; H‐5′b), 3.05 (qd, *J=*3.1, *J=*7.3 Hz, 6 H; 3×CH_2_N ), 2.07 (dt, *J*_5,4_=7.2 Hz, 2 H; H‐5), 2.00–1.98 (m, 2 H; H‐4), 1.68 (s, 3 H; H‐8), 1.63 (s, 3 H; H‐9), 1.59 (s, 3 H; H‐10), 1.31 (t, 9 H; 3×C*H_3_*CH_2_N), 1.12–0.90 ppm (m, 28 H; TIPDS); ^13^C NMR (151 MHz, CDCl_3_): *δ*=139.63 (C‐3), 131.56 (C‐7), 124.03 (C‐6), 120.81 (C‐27), 98.99 (d, *J*_P,1′_=5.7 Hz, C‐1′), 77.10 (C‐3′), 74.62 (d, *J*_P,2′_=9.2 Hz, C‐2′), 64.62 (C‐5′), 63.12 (d, *J*_P,1_=5.2 Hz, C‐1), 61.87 (C‐4′), 45.50 (3×*C*H_2_N), 39.53 (C‐4), 26.43 (C‐5), 25.65 (C‐8), 17.63 (C‐10), 17.59, 17.35, 17.23, 17.19, 17.17, 17.02, 17.00 (8×CH_3_, TIPDS), 16.40 (C‐9), 12.82, 12.72, 12.15, 12.02 (4×CH, TIPDS), 8.48 ppm (3×*C*H_3_CH_2_N); ^31^P NMR (243 MHz, CDCl_3_): *δ*=−2.16 ppm; HRMS (ESI‐TOF): *m*/*z* calcd for C_27_H_52_N_3_O_8_PSi_2_: 632.2958 [*M*−H]^−^; found: 632.2965.

NMR data for **21**: ^1^H NMR (600 MHz, CDCl_3_): *δ*=4.97 (dd, *J*_2,1_=*J*_P,1_=7.2 Hz, 1 H; H‐1), 3.91 (dd, *J*_4,3_=4.1, *J*_2,3_=8.7 Hz, 1 H; H‐3), 3.84–3.79 (m, 2 H; H‐5a, H‐2), 3.73 (dt, *J*_5b,4_=1.6 Hz, 1 H; H‐4), 3.57 (dd, *J*_5b,5a_=12.7 Hz, 1 H; H‐5b), 3.07–3.03 (6 H; 3×CH_2_N), 1.30 (t, 9 H; 3×C*H_3_*CH_2_N), 1.26 (s, 9 H; 3×CH_3_), 1.12–0.92 ppm (m, 28 H; TIPDS); ^13^C NMR (151 MHz, CDCl_3_): *δ*=221.31 (C=O), 96.00 (C‐1), 77.29 (C‐3), 74.55 (d, *J*_P,2_=9.0 Hz, C‐2), 64.46 (C‐5), 61.91 (C‐4), 45.43 [3×CH_2_N, *C*(CH_3_)_3_], 25.66 (3×CH_3_), 17.59, 17.42, 17.37, 17.30, 17.26, 17.23, 17.20, 17.04, 17.02 (8×CH_3_, TIPDS), 12.81, 12.72, 12.16, 12.06, 11.99 (4×CH, TIPDS), 8.45 ppm (3×*C*H_3_CH_2_N);^31^P NMR (243 MHz, CDCl_3_): *δ*=−5.09 ppm.

A second experiment with **5** (50.7 mg, 0.087 mmol), (2*E*)‐3,7‐dimethylocta‐2,6‐dien‐1‐ol (61.1 μL, 0.348 mg) and pivaloyl chloride (48.2 μL) for 22 h at RT followed by oxidation with iodine (33.1 mg) in 2,6‐lutidine/water (50:1, 0.89 mL) for 1 h at 0 °C afforded a mixture of **21**, **22** and **23** (0.19:0.37:1, 17.9 mg). NMR data for **22**: ^1^H NMR (600 MHz, CDCl_3_): *δ*=5.02 (dd, *J*_2,1_=*J*_P,1_=7.4 Hz, 1 H; H‐1), 3.94 (dd, *J*_4,3_=3.8, *J*_2,3_=8.8 Hz, 1 H; H‐3), 3.84–3.79 (m, 2 H; H‐5a, H‐2), 3.75 (dt, *J*_5b,4_=1.7 Hz, 1 H; H‐4), 3.58 (dd, *J*_5b,5a_=12.8 Hz, 1 H; H‐5b), 3.07–3.03 (m, 6 H; 3×CH_2_N), 1.30 (t, 9 H; 3×C*H_3_*CH_2_N), 1.18 (s, 9 H; 3×C*H_3_*), 1.12–0.92 ppm (m, 28 H; TIPDS); ^13^C NMR (151 MHz, CDCl_3_): *δ*=174.95 (HMBC signal, C=O), 96.00 (d, *J*_P,1_=5.4 Hz, C‐1), 77.29 (C‐3), 74.58 (d, *J*_P,2_=9.0 Hz, C‐2), 64.46 (C‐5), 61.91 (C‐4), 45.43 (3×*C*H_2_N), 39.53 [*C*(CH_3_)], 26.92 [C(*C*H_3_)_3_], 17.59, 17.42, 17.37, 17.30, 17.26, 17.23, 17.20, 17.04, 17.02 (8×CH_3_, TIPDS), 12.81, 12.72, 12.16, 12.06, 11.99 (4×CH, TIPDS), 8.45 ppm (3×*C*H_3_CH_2_N); ^31^P NMR (243 MHz, CDCl_3_): *δ*=−9.01 ppm.

**(2*Z*)‐3,7‐Dimethylocta‐2,6‐dien‐1‐yl 1‐*O*‐[4‐azido‐4‐deoxy‐2,3‐*O*‐(tetraisopropyldisiloxane‐1,3‐diyl)‐α‐l‐arabinopyranosyl]phosphate, triethylammonium salt (24)**: *H*‐Phosphonate **5** (113.3 mg, 0.194 mmol) was treated with (2*Z*)‐3,7‐dimethylocta‐2,6‐dien‐1‐ol (171.2 μL, 0.972 mmol) followed by pivaloyl chloride (59.8 μL, 0.486 mmol) and a solution (2 %) of iodine (74 mg, 0.292 mmol) in 2,6‐lutidine/water (50:1, 1.9 mL) as described in General Procedure A. Workup and purification furnished a fraction of **24** containing 9 % of **21** and **22**, followed by a fraction of pure **24** (23.2 mg, 39 % combined yield) as a yellow oil. *R*_f_=0.64 (CHCl_3_/MeOH/25 % aq. NH_3_ 16:4:0.5); [*α*]20D =−24.4 (*c*=1.1 in CHCl_3_); ^1^H NMR (600 MHz, CDCl_3_): *δ*=5.35 (t, *J*_1,2_=6.7 Hz, 1 H; H‐2), 5.06 (t, *J*_5,6_=5.9 Hz, 1 H; H‐6), 4.92 (dd, *J*_2′,1′_=*J*_P,1′_=7.4 Hz, 1 H; H‐1′), 4.46–4.36 (m, 2 H; H‐1a, H‐1b), 3.92 (dd, *J*_4′,3′_=4.2, *J*_2′,3′_=8.7 Hz, 1 H; H‐3′), 3.80 (dd, *J*_4′,5′a_=1.7, *J*_5′b,5′a_=12.9 Hz, 1 H; H‐5′a), 3.81–3.77 (m, 1 H; H‐2′), 3.73 (dt, *J*_5′b,4′_ ≈*J*_5′a,4′_=1.8 Hz, 1 H; H‐4′), 3.57 (dd, 1 H; H‐5′b), 3.04–2,96 (m, 6 H; 3×CH_2_N), 2.05–1.99 (m, 4 H; H‐4, H‐5), 1.68 (d, *J*_2,9_=1.2 Hz, 3 H; H‐9), 1.65 (s, 3 H; H‐8), 1.57 (s, 3 H; H‐10), 1.27 (t, *J=*7.3 Hz, 9 H; 3×C*H_3_*CH_2_N), 1.11–0.98 ppm (m, 28 H; TIPDS); ^13^C NMR (151 MHz, CDCl_3_): *δ*=138.40 (C‐3), 131.49 (C‐7), 124.09 (C‐6), 122.88 (d, *J*_P,2_=8.8 Hz C‐2), 98.86 (d, *J*_P,1′_=6.3 Hz, C‐1′), 77.30 (C‐3′), 74.84 (d, *J*_P,2′_=8.9 Hz, C‐2′), 64.36 (C‐5′), 62.06 (d, *J*_P,6_=5.2 Hz, C‐1), 62.01 (C‐4′), 45.26 (*C*H_2_N), 32.11 (C‐4), 26.75 (C‐5), 25.60 (C‐8), 23.27 (C‐9), 17.63 (C‐10), 17.33, 17.24, 17.17, 17.00, 16.99 (8×CH_3_, TIPDS), 12.77, 12.70, 12.135, 11.95 (4×CH, TIPDS), 8.42 ppm (3×*C*H_3_CH_2_N); ^31^P NMR (243 MHz, CDCl_3_): *δ*=−1.65 ppm; HRMS (ESI‐TOF): *m*/*z* calcd for C_27_H_52_N_3_O_8_PSi_2_+HCOO^−^: 678.3013 [*M*+COOH]^−^; found: 678.3013.

**(2*E*)‐3,7‐Dimethylocta‐2,6‐dien‐1‐yl 1‐*O*‐(4‐amino‐4‐deoxy‐α‐l‐arabinopyranosyl)phosphate, sodium salt** (**25**): Triethylamine trihydrofluoride (33.1 μL, 0.203 mmol) was added at 0 °C to a solution of **23** (43.1 mg, containing 14 % of a mixture of **21** and **22**) in dry THF (170 μL) in a screw‐top Teflon flask and the mixture was stirred at RT under Ar for 20 h. CaCO_3_ (41 mg) and dry THF (170 μL) were then added and the mixture was processed as described in General Procedure B. P(Me)_3_ (27.9 μL, 0.270 mmol) was added at RT under Ar to a solution of the crude intermediate product in THF/0.1 m NaOH (1:2, 15 mL) and the mixture was stirred for 4 h. Extraction and purification as described afforded **25** (4.2 mg, 22 % based on **23**) as a colourless solid. *R*_f_=0.16 (CHCl_3_/MeOH/25 % aq. NH_3_ 2:2:0.1); [*α*]20D =+5.7 (*c*=0.3 in D_2_O); ^1^H NMR (600 MHz, D_2_O, pD 6.8): *δ*=5.38 (tq, *J*_2,9_=1.3, *J*_1,2_=7.1 Hz, 1 H; H‐2), 5.16 (m, *J*_10,6_ ≈*J*_8,6_=1.3, *J*_6,5_=7.0 Hz, 1 H; H‐6), 4.85 (dd, *J*_2′,1′_=*J*_P,1′_=7.3 Hz, 1 H; H‐1′), 4.41 (dd, *J*_P,1_=6.8 Hz, 2 H; H‐1a, H‐1b), 4.03 (dd, *J*_4′,5′a_=2.2, *J*_5′b,5′a_=13.4 Hz, 1 H; H‐5′a), 3.97 (dd, *J*_4′,3′_=4.4, *J*_2′,3′_=8.7 Hz, 1 H; H‐3′), 3.80 (dd, *J*_4′,5′b_=2.3, 1 H; H‐5′b), 3.60 (dt, 1 H; H‐4′), 3.57 (dd, 1 H; H‐2′), 2.14 (dt, *J=*7.4 Hz, 2 H; H‐5), 2.07–2.04 (m, 2 H; H‐4), 1.66 (s, 3 H; H‐9), 1.65 (s, 3 H; H‐8), 1.59 ppm (s, 3 H; H‐10); ^13^C NMR (151 MHz, D_2_O): *δ*=143.96 (C‐3), 134.56 (C‐7), 124.84 (C‐6), 120.07 (d, *J*_P,2_=7.8 Hz, C‐2), 98.75 (d, *J*_P,1′_=5.5 Hz, C‐1′), 71.33 (d, *J*_P,2′_=8.8 Hz, C‐2′), 69.46 (C‐3′), 63.79 (d, *J*_P,1_=5.5 Hz, C‐1), 62.66 (C‐5′), 51.35 (C‐4′), 39.50 (C‐4), 26.34 (C‐5), 25.58 (C‐8), 17.73 (C‐10), 16.32 ppm (C‐9); ^31^P NMR (243 MHz, D_2_O): *δ*=−1.22 (d, *J*_1,P_=7.4 Hz); HRMS (ESI‐TOF): *m*/*z* calcd for C_15_H_28_NO_7_P: 366.1676 [*M*+H]^+^; found: 366.1682.

**(2*Z*)‐3,7‐Dimethylocta‐2,6‐dien‐1‐yl 1‐*O*‐(4‐amino‐4‐deoxy‐α‐l‐arabinopyranosyl)phosphate, sodium salt** (**26**): Triethylamine trihydrofluoride (38.1 μL, 0.233 mmol) was added at 0 °C to a solution of **24** (58.5 mg, containing 5 % of a mixture of **21** and **22**) in dry THF (190 μL) in a screw‐top Teflon flask and the mixture was stirred at RT under Ar for 19 h. CaCO_3_ (47 mg) and dry THF (190 μL) were added and the reaction mixture was processed as described in General Procedure B. P(Me)_3_ (32.1 μL, 0.310 mmol) was added at RT under Ar to a solution of the crude intermediate product in THF/0.1 m NaOH (1:2, 17 mL) and the mixture was stirred for 4 h. Extraction and purification as described afforded **26** (6.8 mg, 23 %) as a colourless solid. *R*_f_=0.18 (CHCl_3_/MeOH/25 % aq. NH_3_ 2:2:0.1); [*α*]20D =+6.6 (*c*=0.3 in D_2_O); ^1^H NMR (600 MHz, D_2_O, pD 6.5): *δ*=5.39 (tq, *J*_2,9_=1.2, *J*_1,2_=7.3 Hz, 1 H; H‐2), 5.15 (m, *J*_10,6_ ≈*J*_8,6_=1.4, *J*_6,5_=6.4 Hz, 1 H; H‐6), 4.85 (dd, *J*_2′,1′_=*J*_P,1′_=7.2 Hz, 1 H; H‐1′), 4.39 (app t, *J*_P,1_=6.8 Hz, 2 H; H‐1a, H‐1b), 4.05 (dd, *J*_4′,5′a_=3.2, *J*_5′b,5′a_=13.4 Hz, 1 H; H‐5′a), 3.99 (dd, *J*_4′,3′_=4.4, *J*_2′,3′_=8.6 Hz, 1 H; H‐3′), 3.81 (dd, *J*_4′,5′b_=2.3 Hz, 1 H; H‐5′b), 3.63 (dt, 1 H; H‐4′), 3.57 (dd, 1 H; H‐2′), 2.14–2.09 (m, 4 H; H‐4, H‐5), 1.73 (s, 3 H; H‐9), 1.65 (s, 3 H; H‐8), 1.58 ppm (s, 3 H; H‐10); ^13^C NMR (151 MHz, D_2_O): *δ*=144.18 (C‐3), 134.85 (C‐7), 124.65 (C‐6), 121.05 (d, *J*_P,2_=7.9 Hz, C‐2), 98.77 (d, *J*_P,1′_=6.0 Hz, C‐1′), 71.39 (d, *J*_P,2′_=8.6 Hz, C‐2′), 69.41 (C‐3′), 63.62 (d, *J*_P,1_=5.3 Hz, C‐1), 62.57 (C‐5′), 51.41 (C‐4′), 32.10 (C‐4), 26.78 (C‐5), 25.69 (C‐8), 23.47 (C‐9), 17.80 ppm (C‐10); ^31^P NMR (243 MHz, D_2_O): *δ*=−1.30 ppm (d, *J*_1,P_=5.85 Hz); HRMS (ESI‐TOF): *m*/*z* calcd for C_15_H_28_NO_7_P+HCOO^−^: 410.1585 [*M*+COOH]^−^; found: 410.1584.

**Expression and membrane preparation of ArnT**: ArnT‐FLAG‐His_10_ recombinant protein was expressed as previously reported.14a, 35 pFT1 plasmid encoding the *B. cenocepacia* arnT‐FLAG‐His_10_ was introduced into *E. coli* DH5α by CaCl_2_ transformation. For protein expression, cells were grown in lysogeny broth (LB, 1 L) medium supplemented with ampicillin (100 μg mL^−1^) at 37 °C with shaking until *A*_600_ reached 0.5–0.6 and were induced with l‐arabinose (0.2 %, *w*/*v*) for 4 h. Cells were harvested by centrifugation (10 min at 11 270 *g* and 4 °C) and stored at −20 °C. All subsequent steps were carried out at 4 °C. Bacterial pellets were resuspended in Tris**⋅**HCl (pH 8.0, 50 mm) with protease inhibitors and lysed at 25 000 psi with a cell disruptor (Constant Systems, Kennesaw, GA). Cell debris was pelleted by centrifugation at 9590 *g* for 15 min at 4 °C, and aliquots of the supernatant were centrifuged at 42 220 *g* for 1 h to collect total membranes. One pellet containing crude membranes was resuspended in Tris**⋅**HCl (pH 8.0, 50 mm). Proteins were separated by SDS‐PAGE (12 %) and transferred to a nitrocellulose membrane. Immunoblots were probed with anti‐FLAG mouse monoclonal antibodies (Sigma) for 1 h. IRDye 800CW Goat anti‐Mouse IgG (LI‐COR) was used as secondary antibody. Reacting bands were detected with an Odyssey infrared imaging system (Li‐cor Bioscience, Lincoln, NE). Protein concentration was determined by the Bradford assay (Bio‐Rad).

**Assay conditions to detect ArnT activity**: ArnT from *B. cenocepacia* was assayed in a reaction mixture (100 μL) consisting of MES buffer (pH 6.5, 50 mm), Triton X‐100 (0.2 %) and Kdo_2_‐lipid A (KLA, 30 μm) as acceptor. Crude membranes from ArnT expression (1 mg mL^−1^) were used as the source of l‐Ara4N transferase; synthesized α‐ and β‐Ara4N‐lipid phosphodiester derivatives (150 μm) were used as donor. Reaction mixtures were incubated at 30 °C for 17 h. The samples were concentrated with a speed vacuum concentrator for 40 min, dissolved in CHCl_3_/MeOH (4:1, 2×30 μL), and spotted (5 μL portions) onto prewashed silica gel 60 TLC plates (prewashing of the plates in CHCl_3_/pyridine/88 % formic acid/water (50:50:16:5) for 1 h with shaking at 180 rpm before drying overnight). Reaction products were separated in CHCl_3_/pyridine/88 % formic acid/water (50:50:16:5), and the plates were dried for 2 h at room temperature. The bands corresponding to the KLA or modified KLA products could be seen transiently as white zones while the TLC plates were drying at RT and subsequently by staining of a lane cut from the plate with anisaldehyde/H_2_SO_4_. TLC areas containing the putative KLA and l‐Ara4N‐modified KLA derivatives were removed with a clean knife and the lipids were extracted from the chips with an acidic single‐phase Bligh–Dyer mixture (304 μL) containing CHCl_3_/MeOH/0.1 m HCl (1:2:0.8). After vortexing of the mixture and sonication for 40 min, solids were removed by centrifugation at 11 000 *g* for 10 min and the supernatant was transformed into a two‐phase Bligh–Dyer system by adding chloroform (80 μL) and water (80 μL). After vortexing and centrifugation of the samples at 2000 *g* for 10 min, the lower phase containing the lipids was withdrawn and dried with a speed vacuum concentrator for 15 min at RT. For each synthetic Ara4N‐lipid phosphodiester donor three separate assays were carried out, followed by dissolution in CHCl_3_/MeOH (4:1, 3×50 μL). The combined solutions were concentrated in one vial and stored at 4 °C prior to mass spectrometric analysis.

**Mass spectrometry (MS) of Kdo_2_‐lipid A (KLA) and l‐Ar4N‐modified KLA derivative**: Samples containing the lipids were reconstituted in 14 μL of solvent A (85 %, MeOH/160 mm ammonia formate buffer, pH 9, 3:1) and solvent B (15 %, CHCl_3_/MeOH 4:1). The solution (5 μL) was directly injected into a LC‐ESI‐MS system (HPLC, Dionex Ultimate 3000 LC). A gradient procedure from 25 to 60 % solvent B was carried out with use of a reversed‐phase C_5_ HPLC column (Supelco C5, 0.32 mm ID, 50 mm) at a flow rate of 6 μL min^−1^. Detection was performed with a Q‐TOF instrument (Bruker maXis 4G) equipped with the standard ESI source in positive ion, DDA mode. MS scans were recorded (range 150–2200 Da) and the three highest peaks were selected for fragmentation (precursor ion selection was limited to mass ranges of interest (1119.1–1120.1, 1184.7–1185.7, 1192.2–1193.2, 1195.7–1196.7 and 1250.8–1251.8). Instrument calibration was performed with ESI calibration mixture (Agilent). For the quantification of the different KLA derivatives the peak areas of EICs (Extracted Ion Chromatograms) of the first four isotopic peaks were summed and compared by using Data Analysis 4.0 (Bruker).

## Conflict of interest

*The authors declare no conflict of interest*.

## Supporting information

As a service to our authors and readers, this journal provides supporting information supplied by the authors. Such materials are peer reviewed and may be re‐organized for online delivery, but are not copy‐edited or typeset. Technical support issues arising from supporting information (other than missing files) should be addressed to the authors.

Supplementary
