# Supplementary material for: Synthetic Phosphodiester‐Linked 4‐Amino‐4‐deoxy‐l‐arabinose Derivatives Demonstrate that ArnT is an Inverting Aminoarabinosyl Transferase
Source: Chembiochem. 2019 Oct 22;20(23):2936–48. doi: 10.1002/cbic.201900349 (PMC6902282; doi:10.1002/cbic.201900349)
Supplement: Supplementary file 1 — Supplementary [file CBIC-20-2936-s001.pdf]

CHEMBIOCHEM

Supporting Information

Synthetic Phosphodiester-Linked 4-Amino-4-deoxy-L-arabinose Derivatives Demonstrate that ArnT is an Inverting Aminoarabinosyl Transferase

Charlotte Olagnon,[a] Julia Monjaras Feria,[b] Clemens Grünwald-Gruber,[a] Markus Blaukopf,[a] Miguel A. Valvano,[b] and Paul Kosma*[a]

cbic_201900349_sm_miscellaneous_information.pdf

Supporting Information

Contents | Page

Contents	Page
NMR spectra of compounds **7, 9-17, 19, 20, 23-26**	S2-S28
Table S1	S29
Peptide analysis	S30
References SI	S30

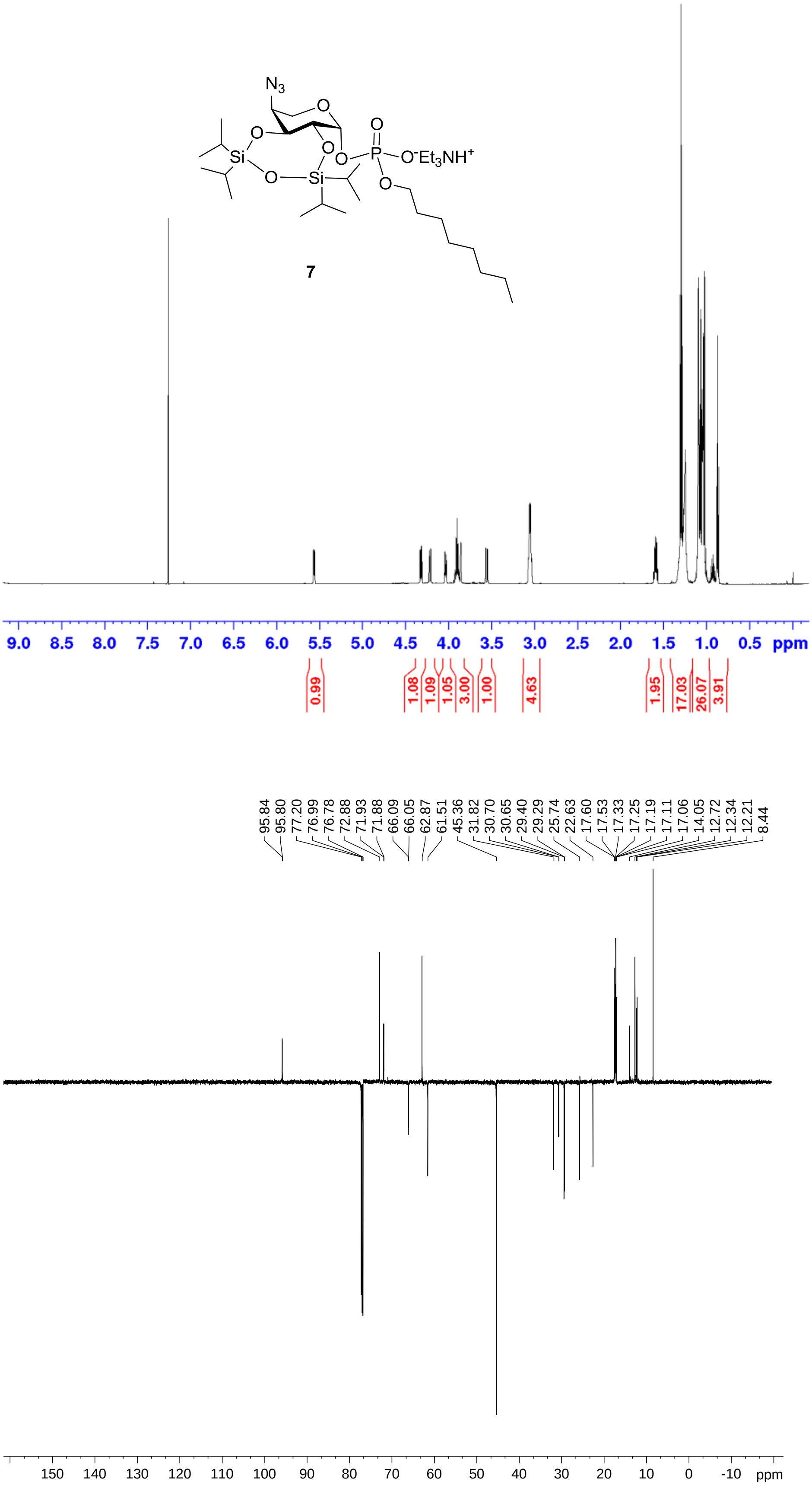

Fig. S1: ^{1}H and ^{13}C NMR spectrum of **7**.

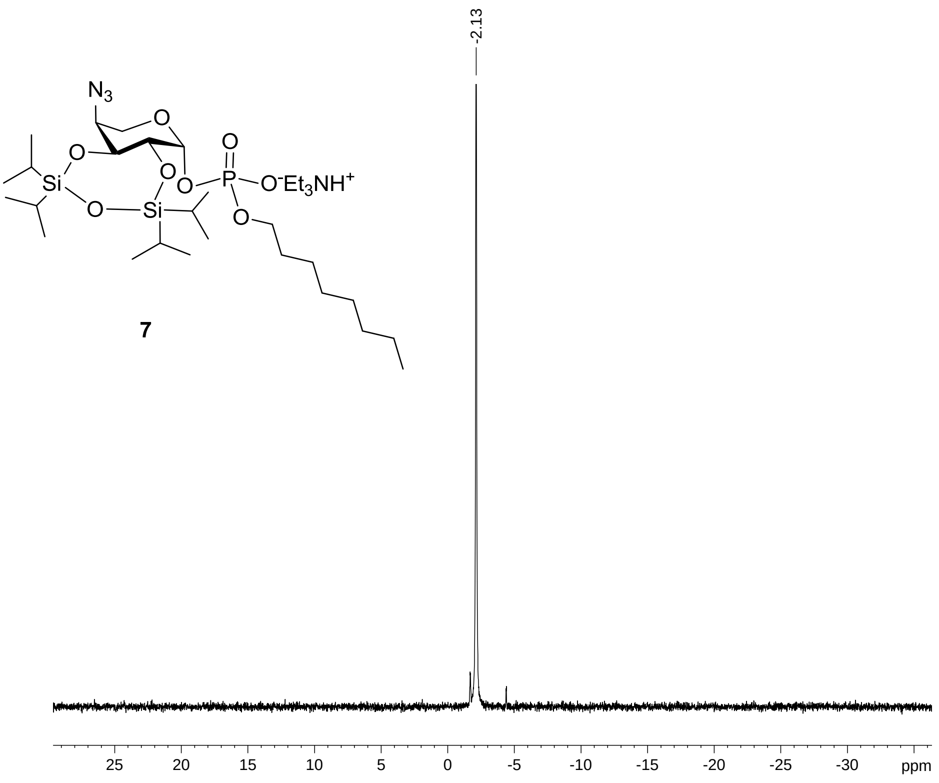

Fig. S2: ^{31}P NMR spectrum of **7**.

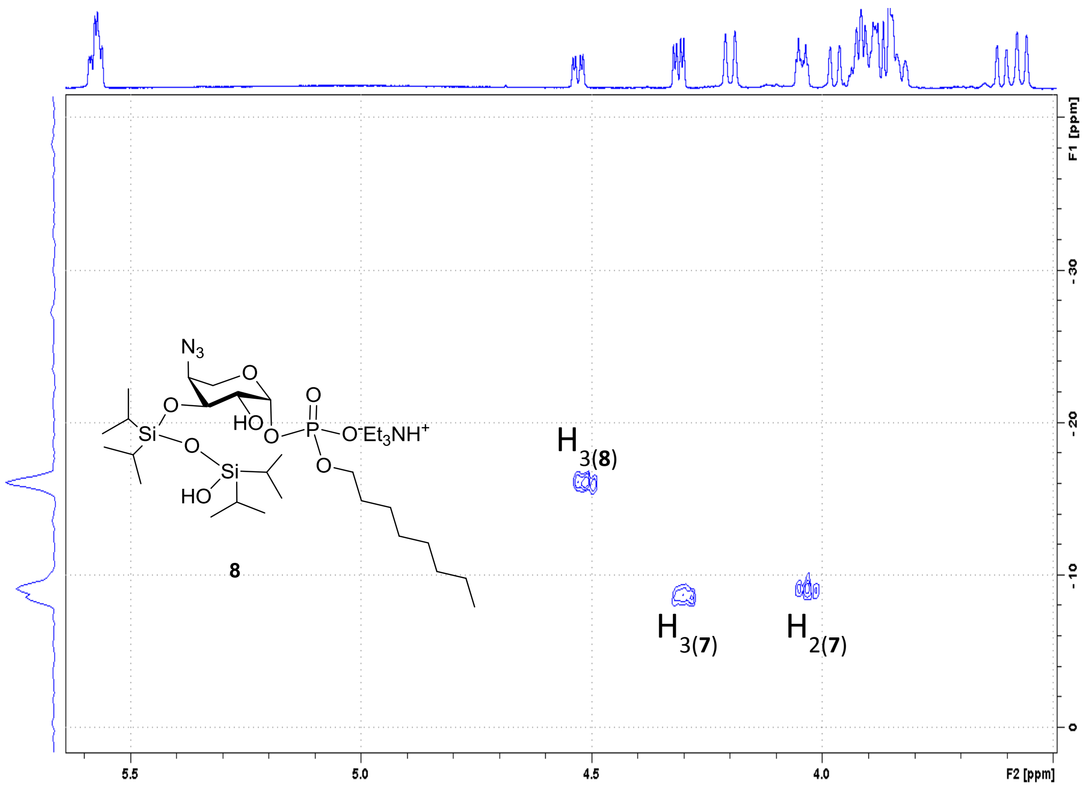

Fig. S3: ^{29}Si HSQC NMR spectrum of **8** (in a mixture with **7**).

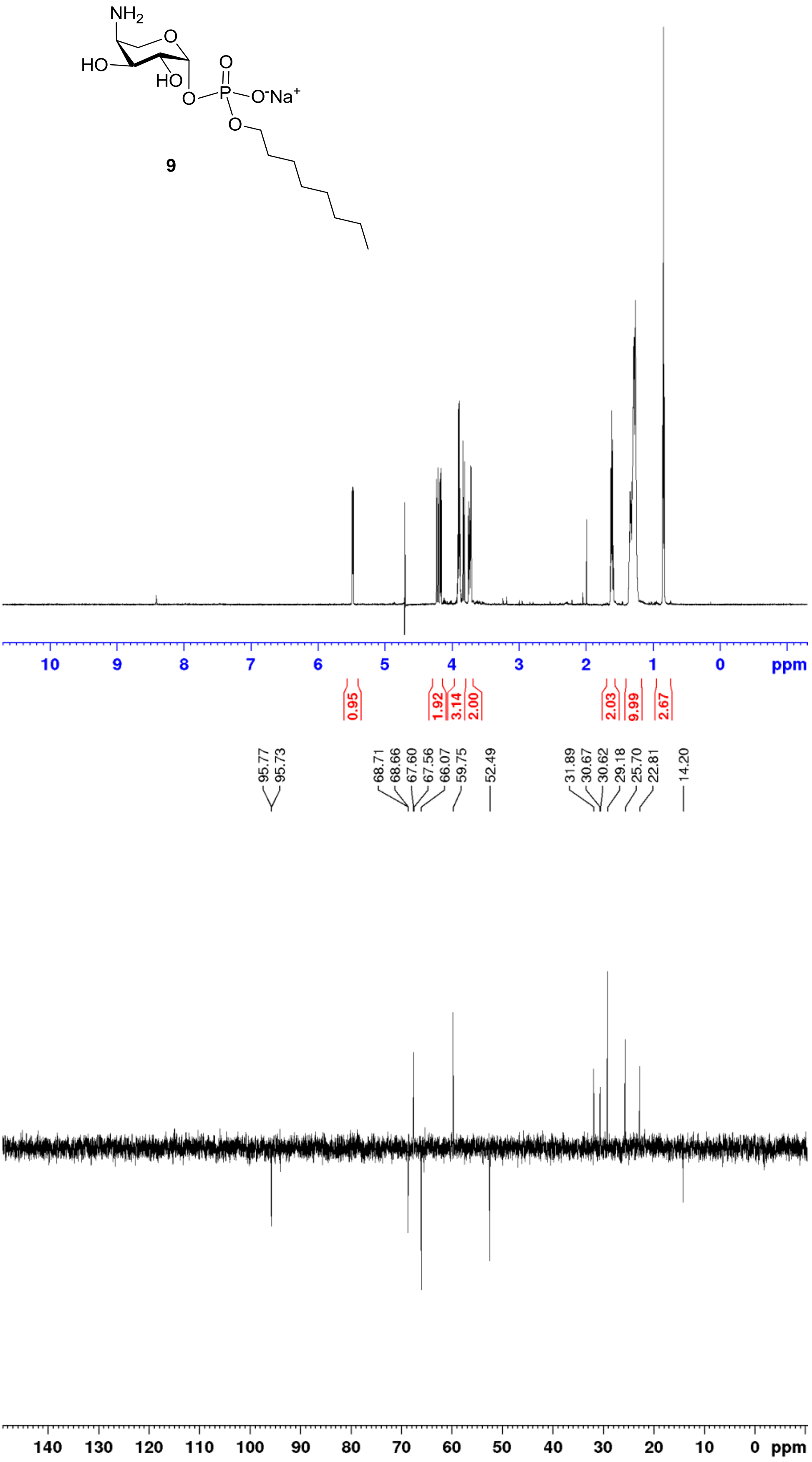

Fig. S4: ^{1}H and ^{13}C NMR spectrum of **9**.

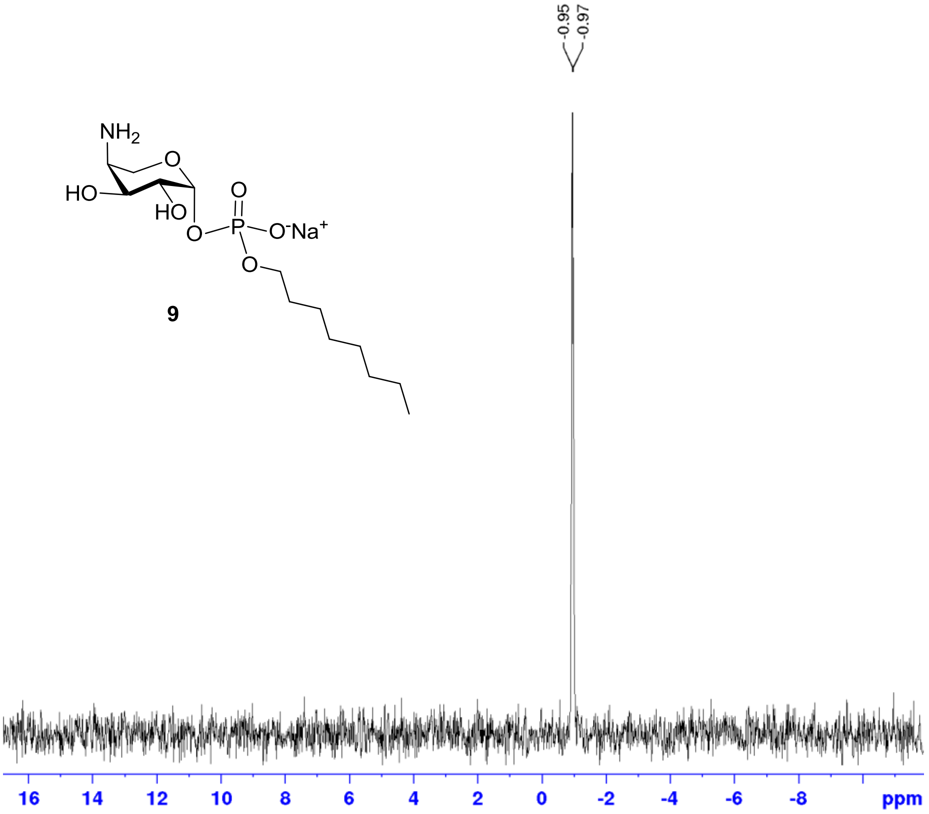

Fig. S5: ^{31}P NMR spectrum of **9**.

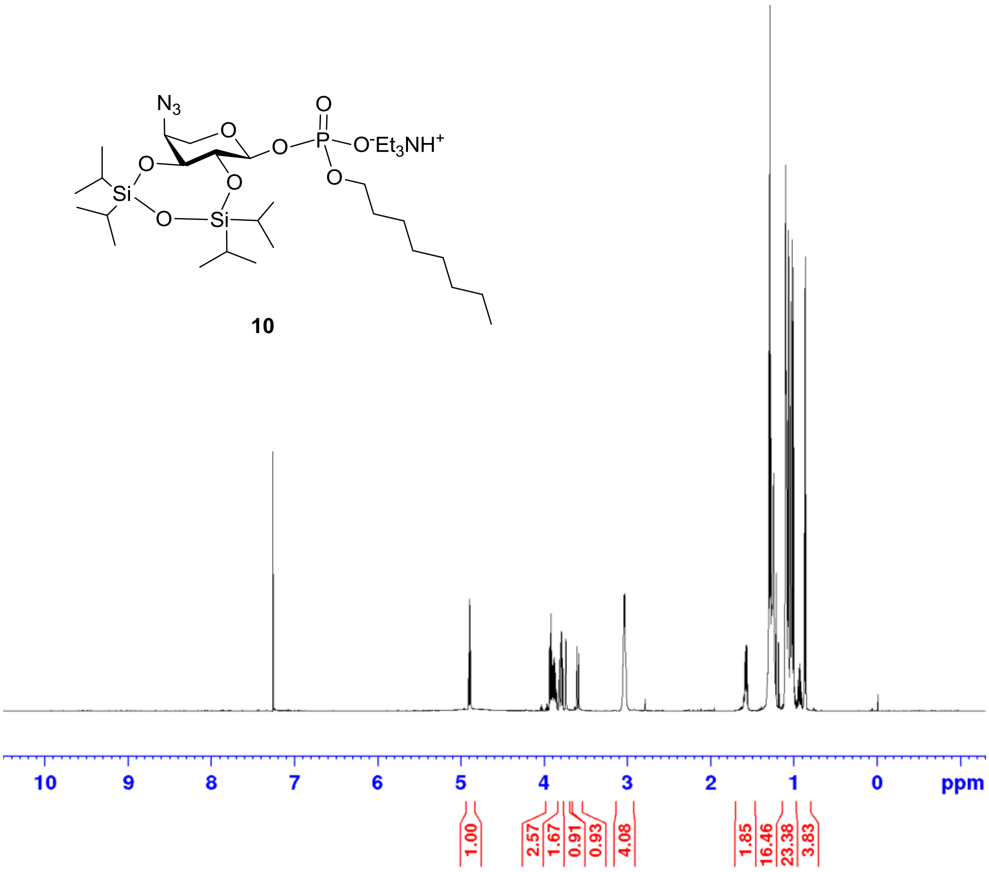

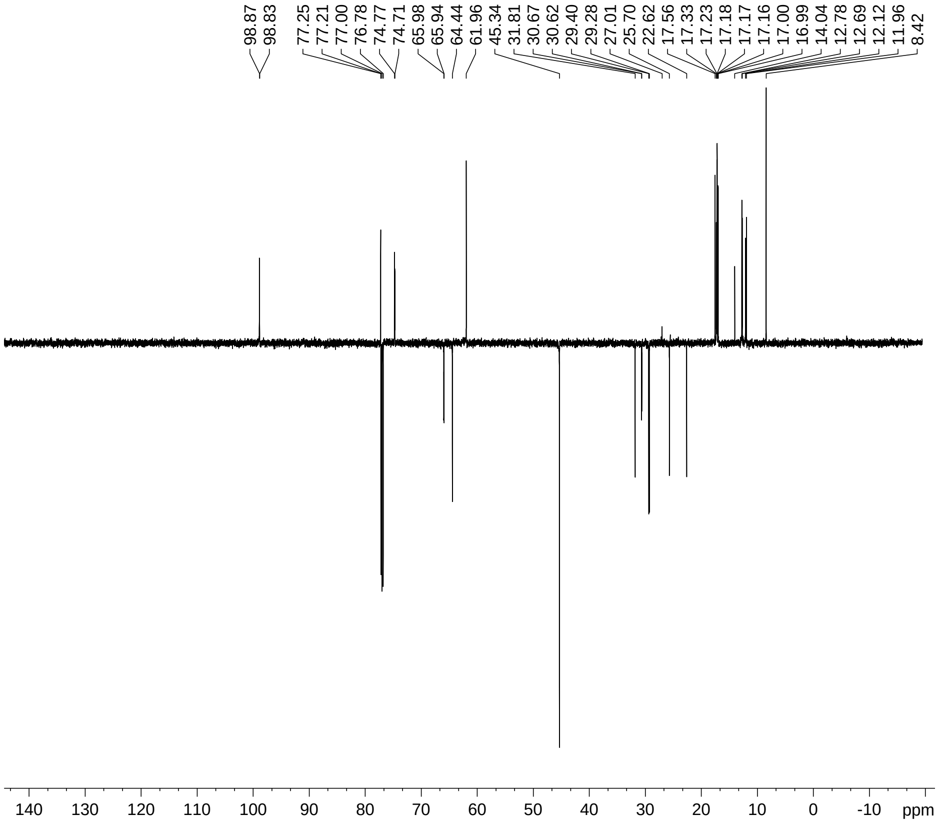

Fig. S6: ^{1}H and ^{13}C NMR spectrum of **10**.

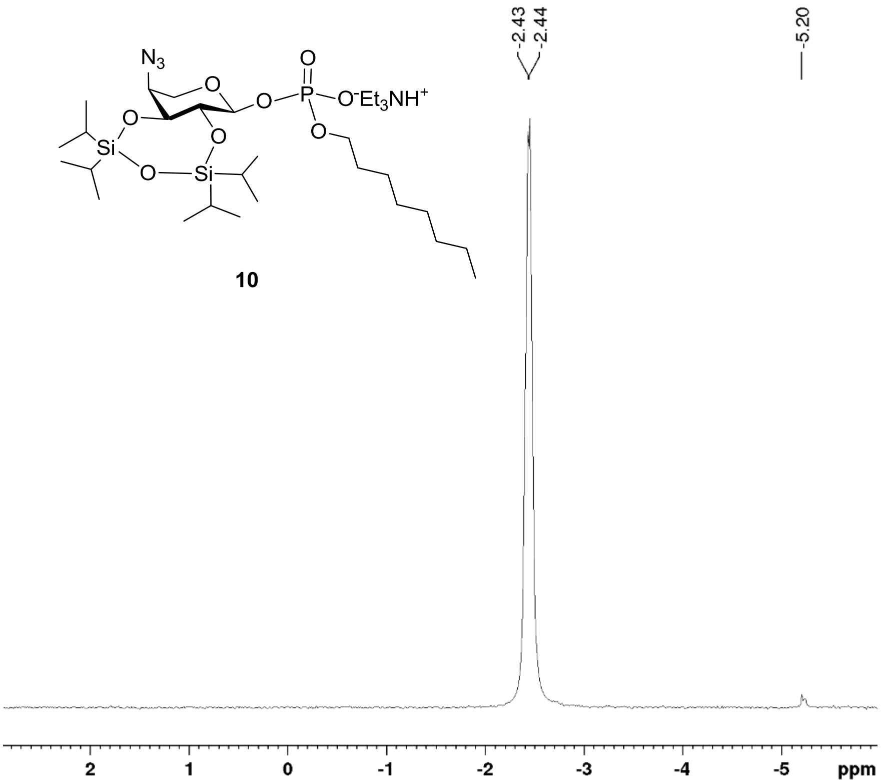

Fig. S7: ^{31}P NMR spectrum of **10**.

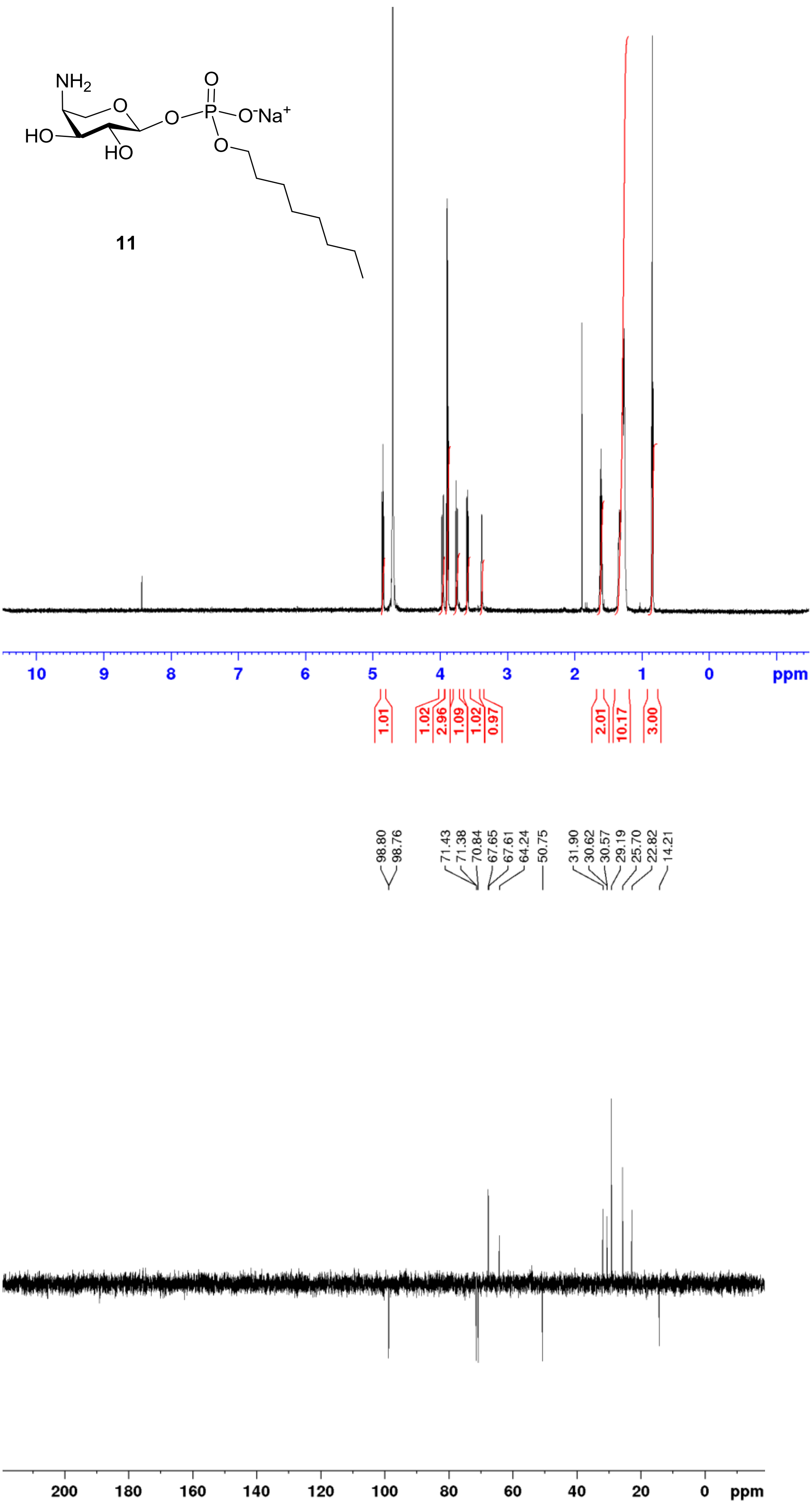

Fig. S8: ^{1}H and ^{13}C NMR spectrum of **11**.

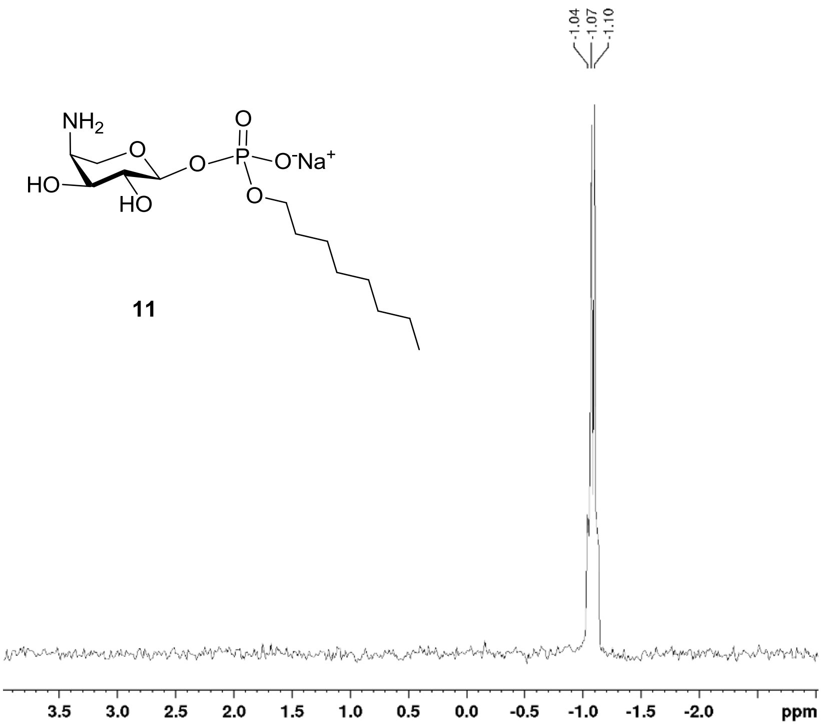

Fig. S9: ^{31}P NMR spectrum of **11**.

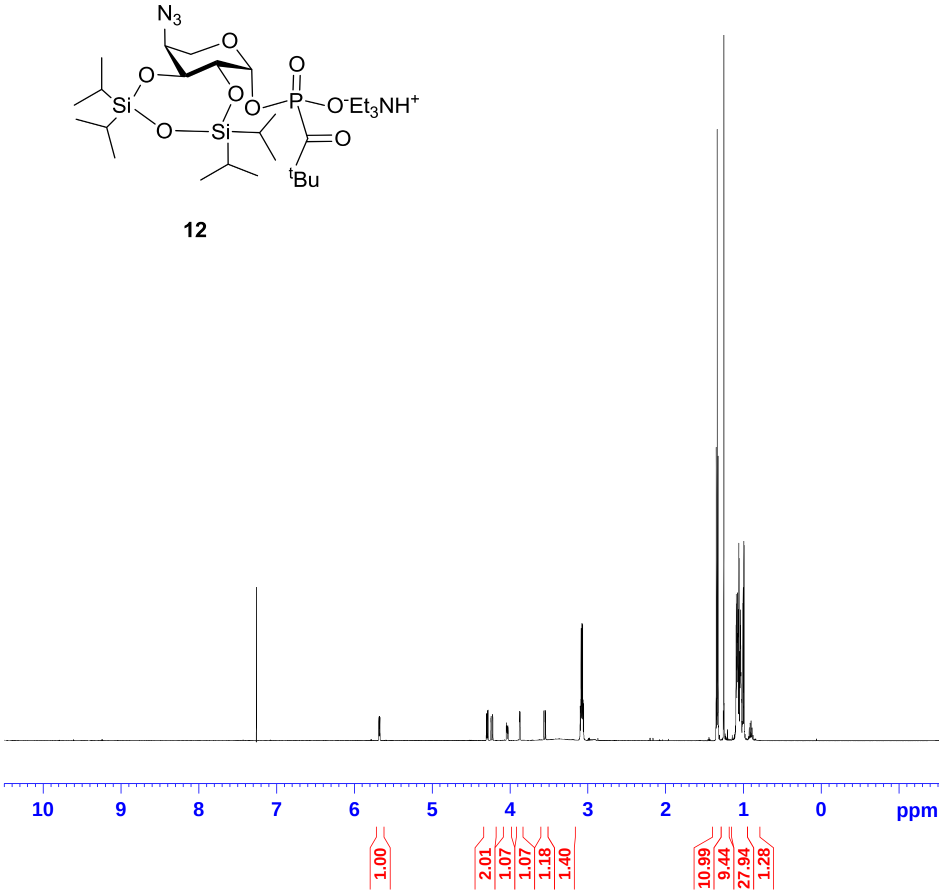

Fig. S10: ^{1}H NMR spectrum of **12**.

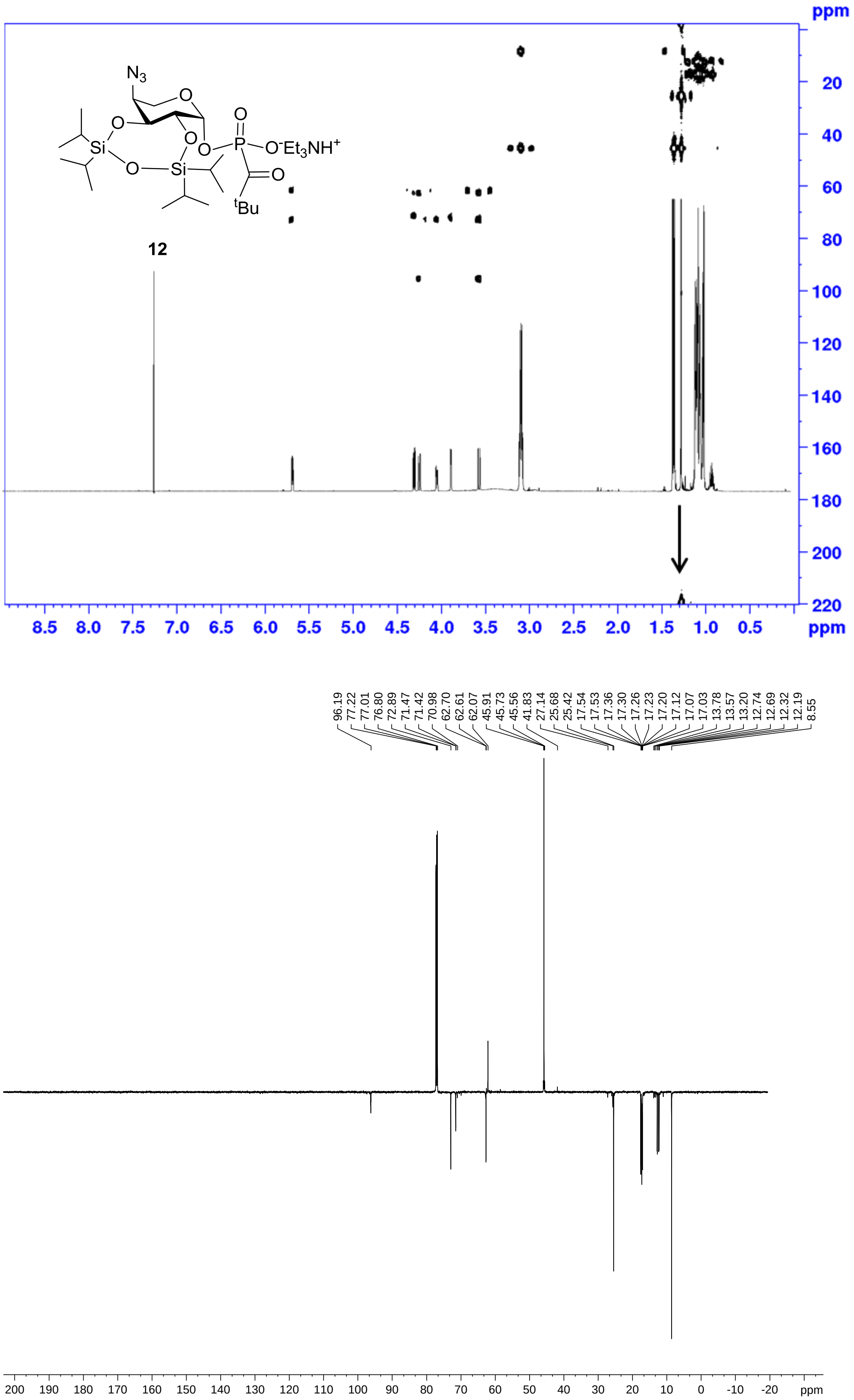

Fig. S11: HMBC and ^{13}C NMR spectrum of **12**.

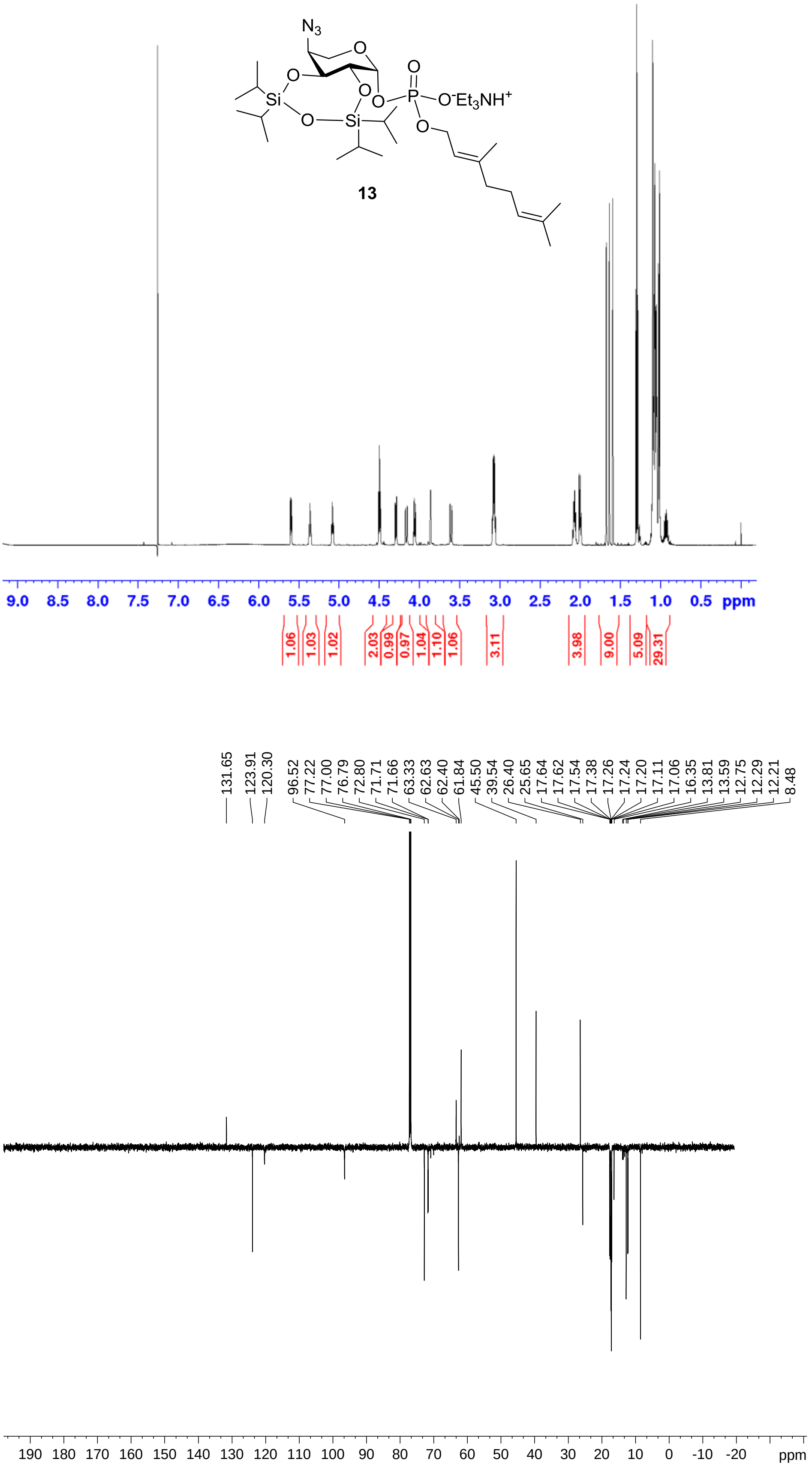

Fig. S12: ^{1}H and ^{13}C NMR spectrum of **13**.

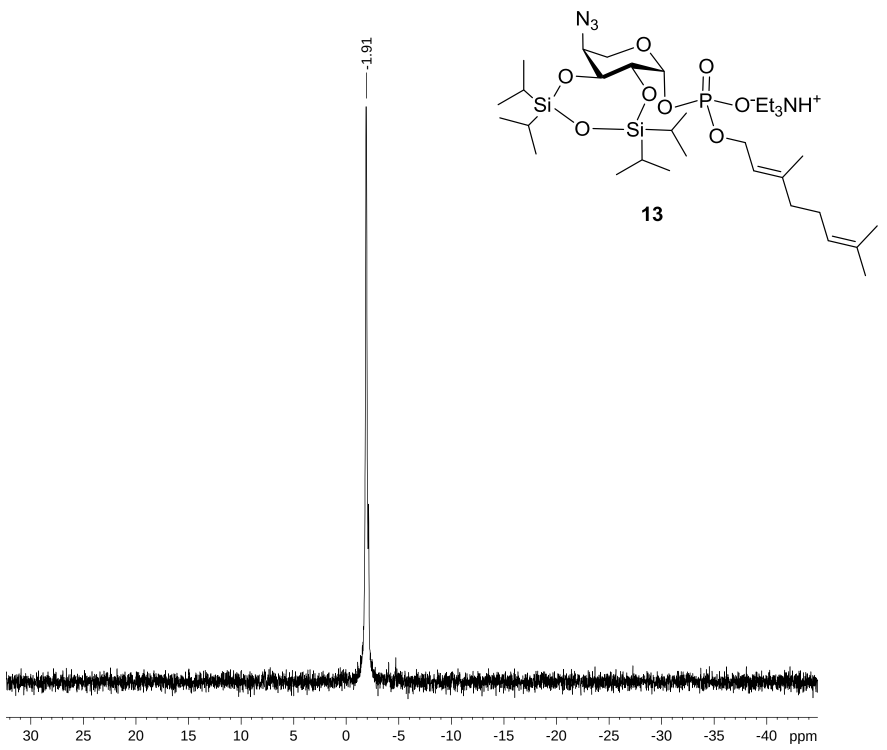

Fig. S13: ^{31}P NMR spectrum of **13**.

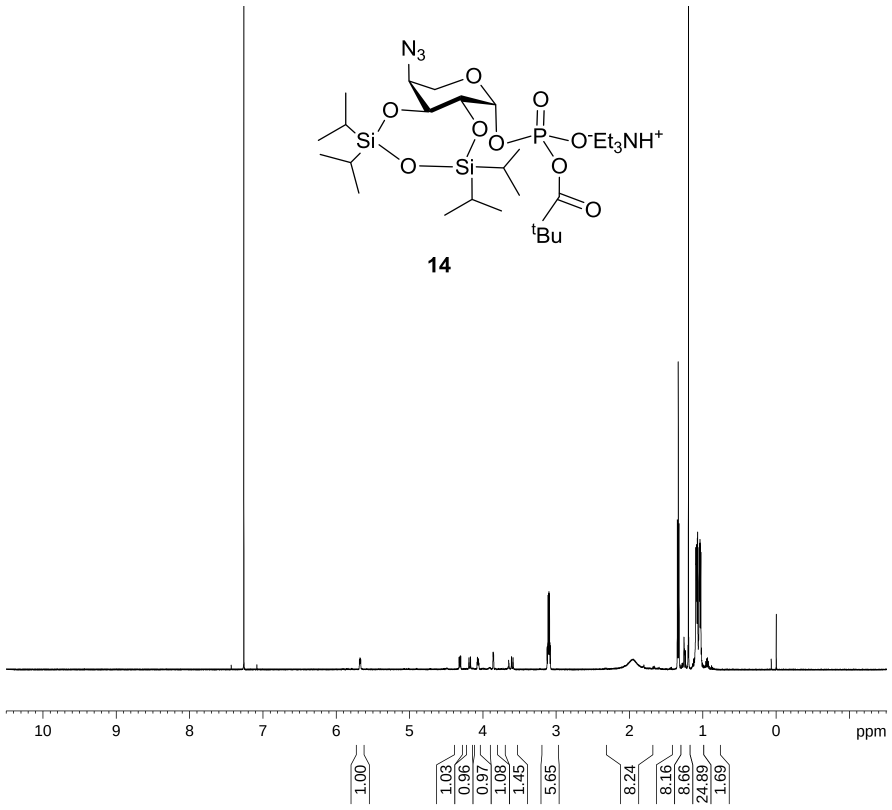

Fig. S14: ^{1}H NMR spectrum of **14**.

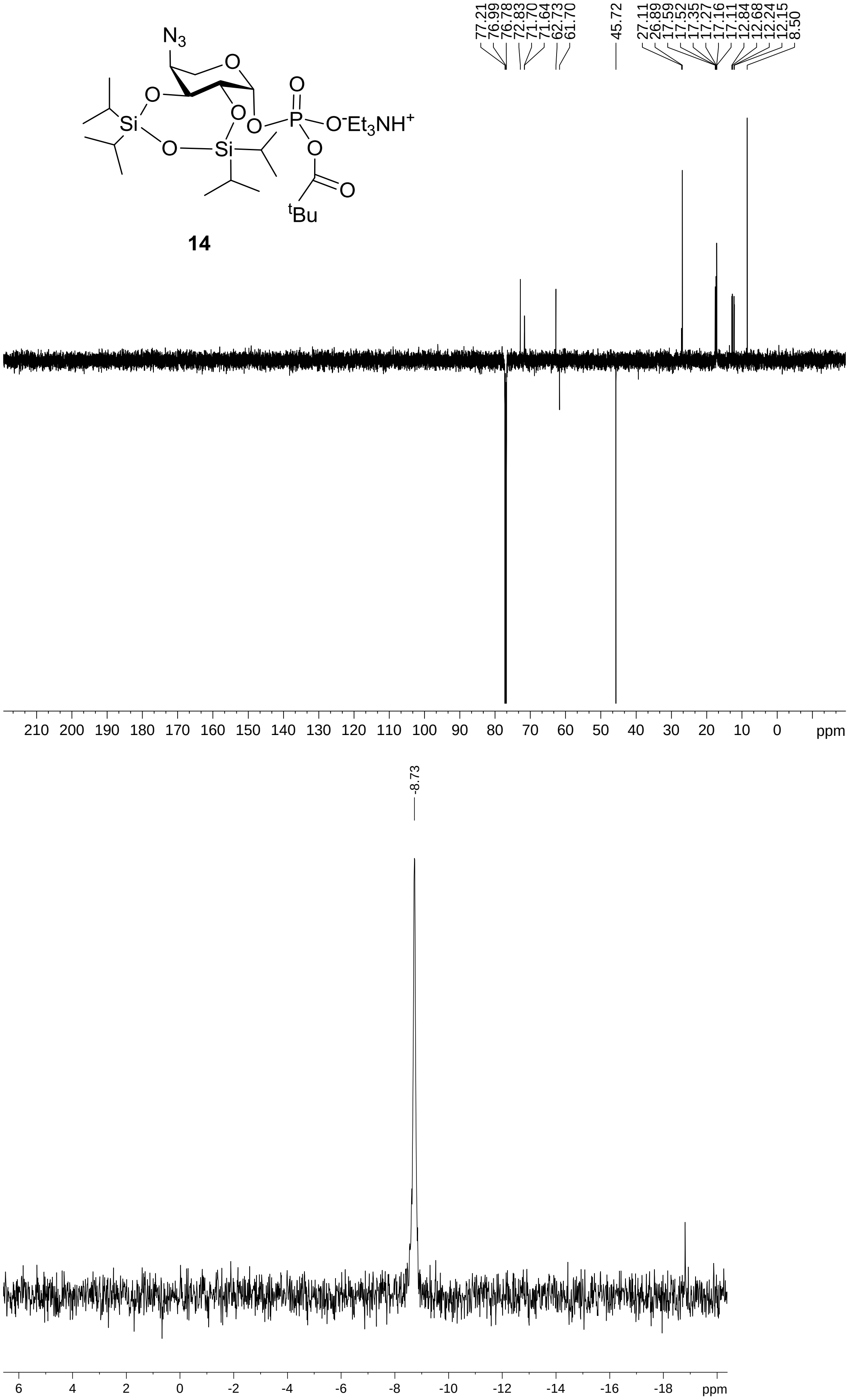

Fig. S15: ^{13}C and ^{31}P NMR spectrum of **14**.

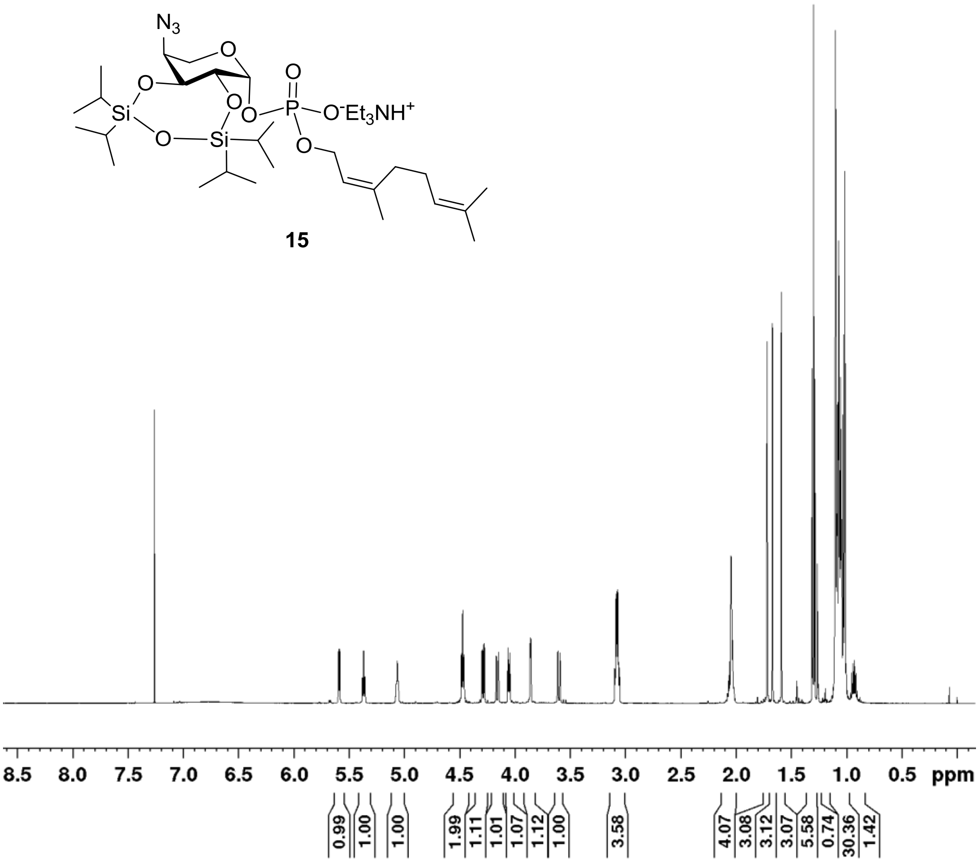

15

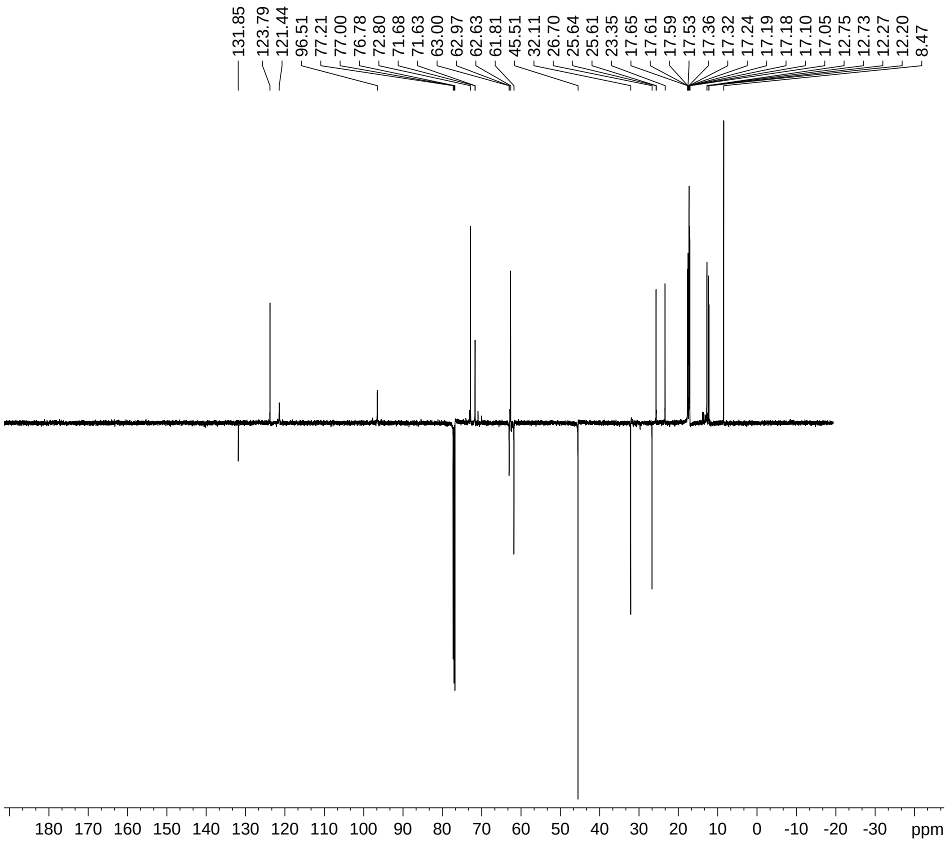

Fig. S16: ^{1}H NMR and ^{13}C NMR spectrum of **15**.

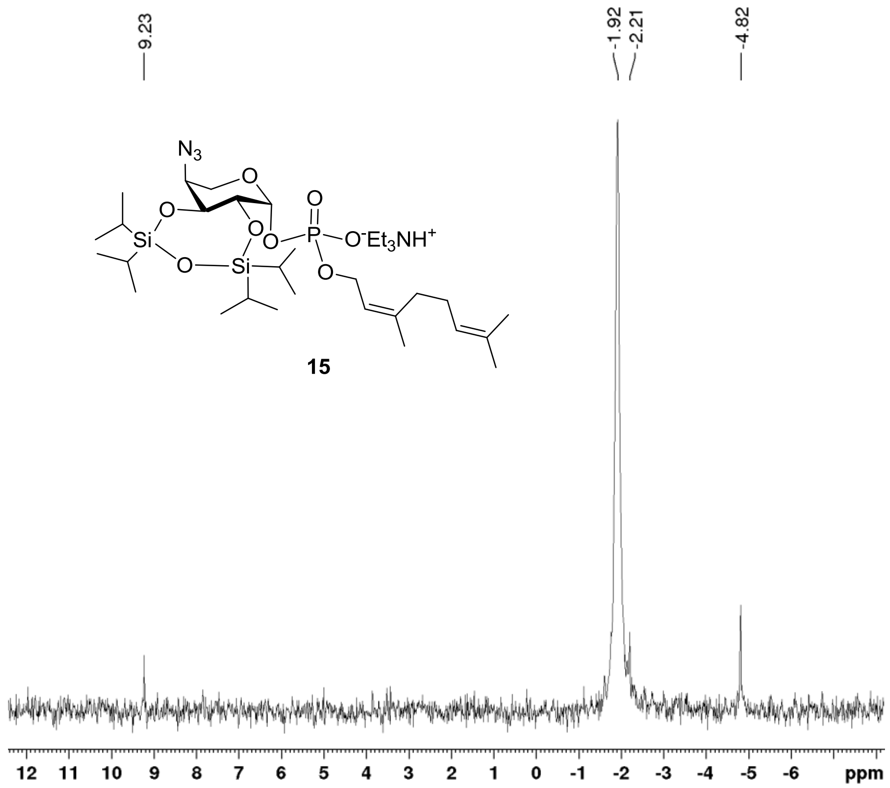

Fig. S17: ^{31}P NMR spectrum of **15**.

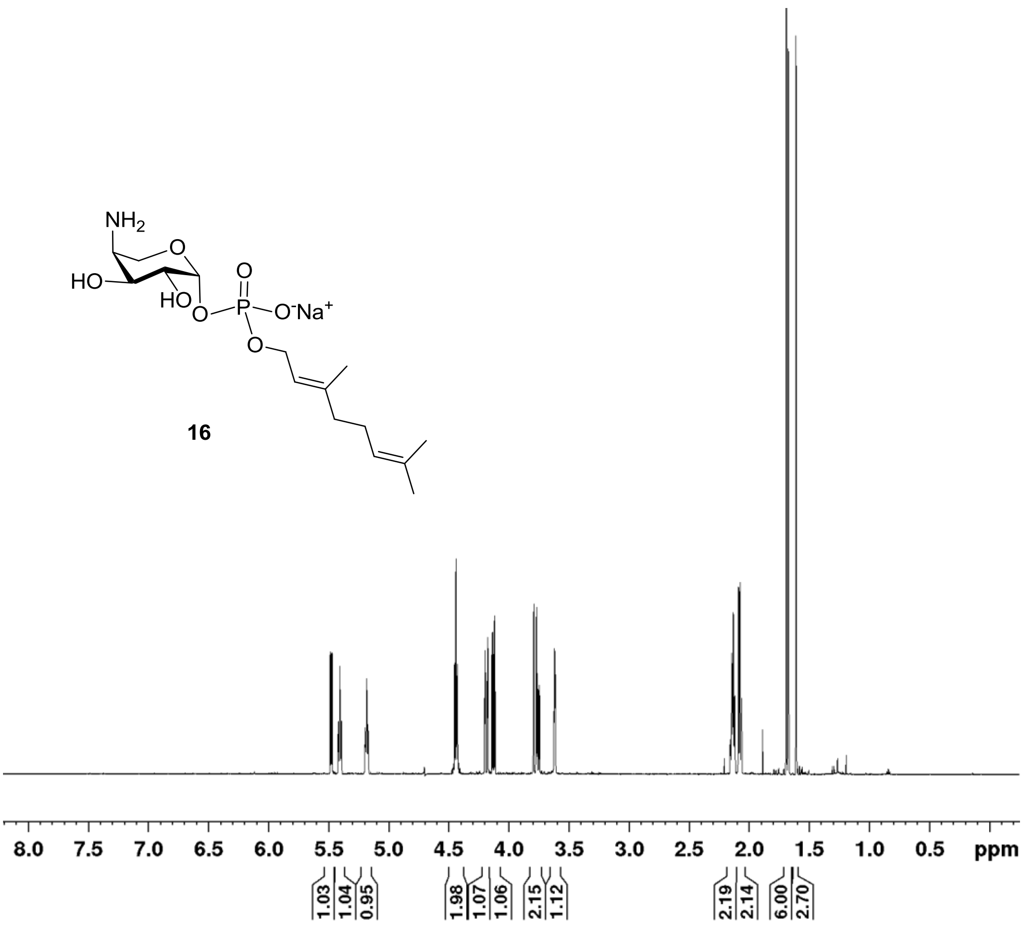

Fig. S18: ^{1}H NMR spectrum of **16**.

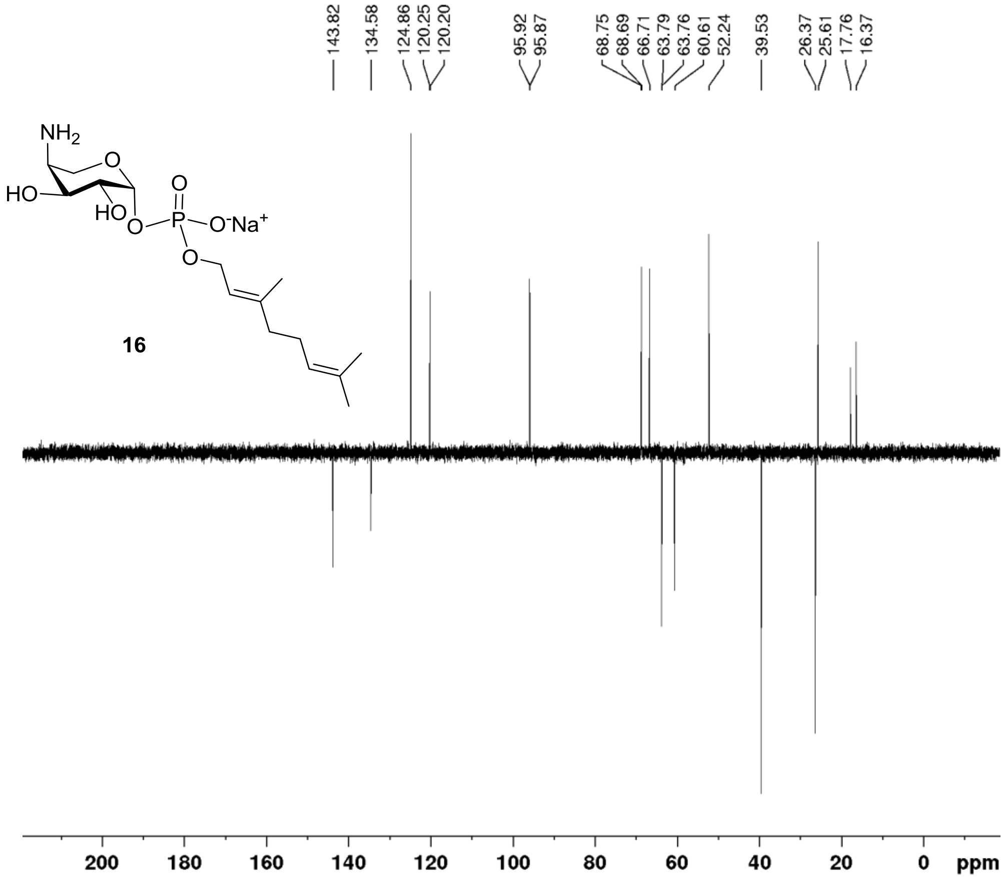

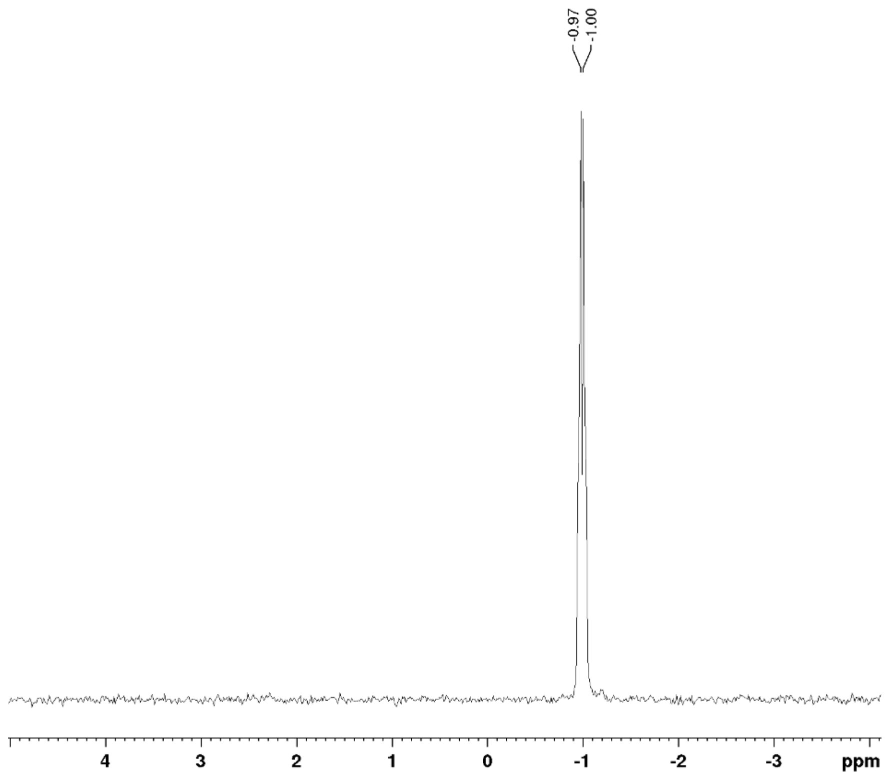

Fig. S19: ^{13}C and ^{31}P NMR spectrum of **16**.

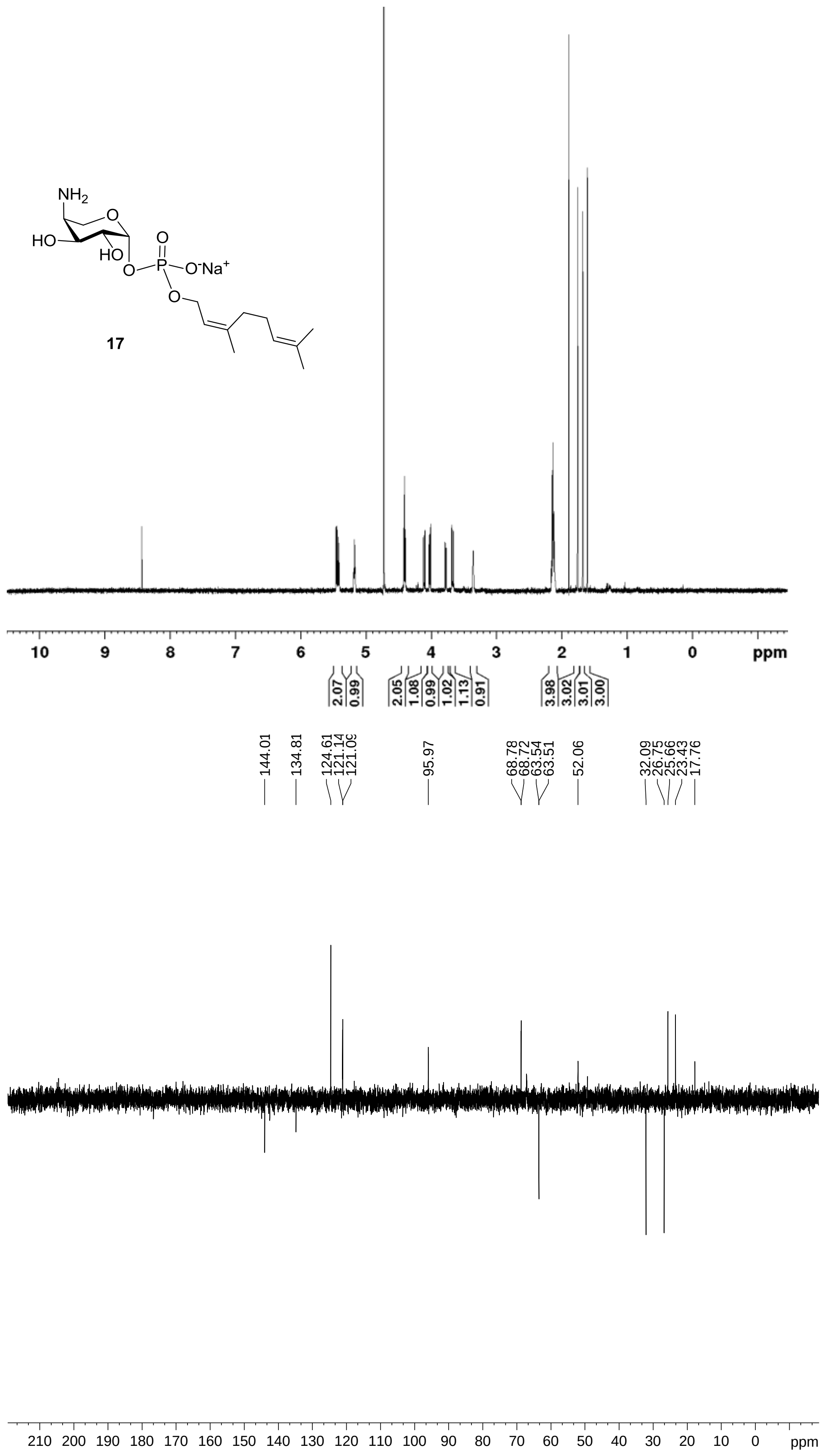

Fig. S20: ^{1}H and ^{13}C NMR spectrum of **17**.

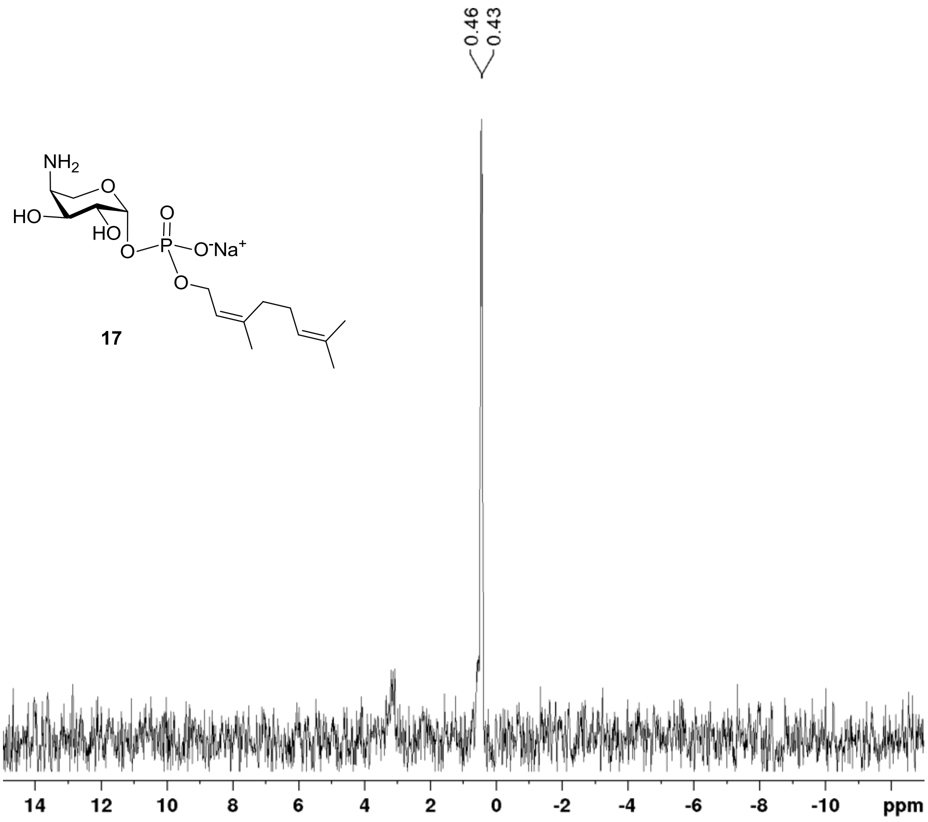

Fig. S21 ^{31}P NMR spectrum of **17**.

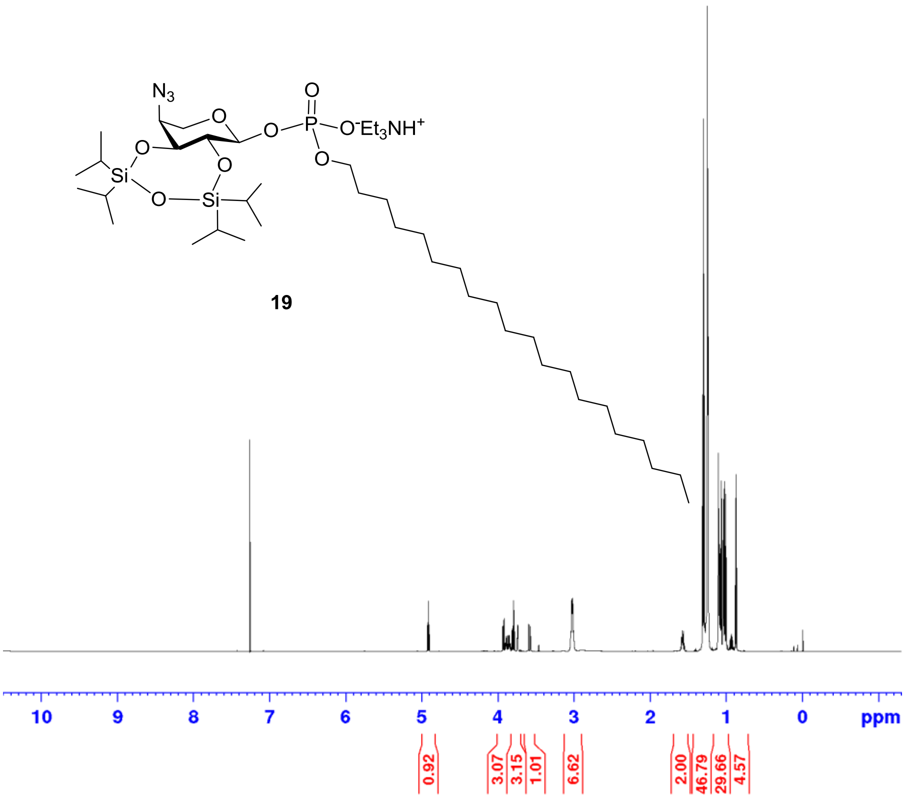

Fig. S22 ^{1}H NMR spectrum of **19**.

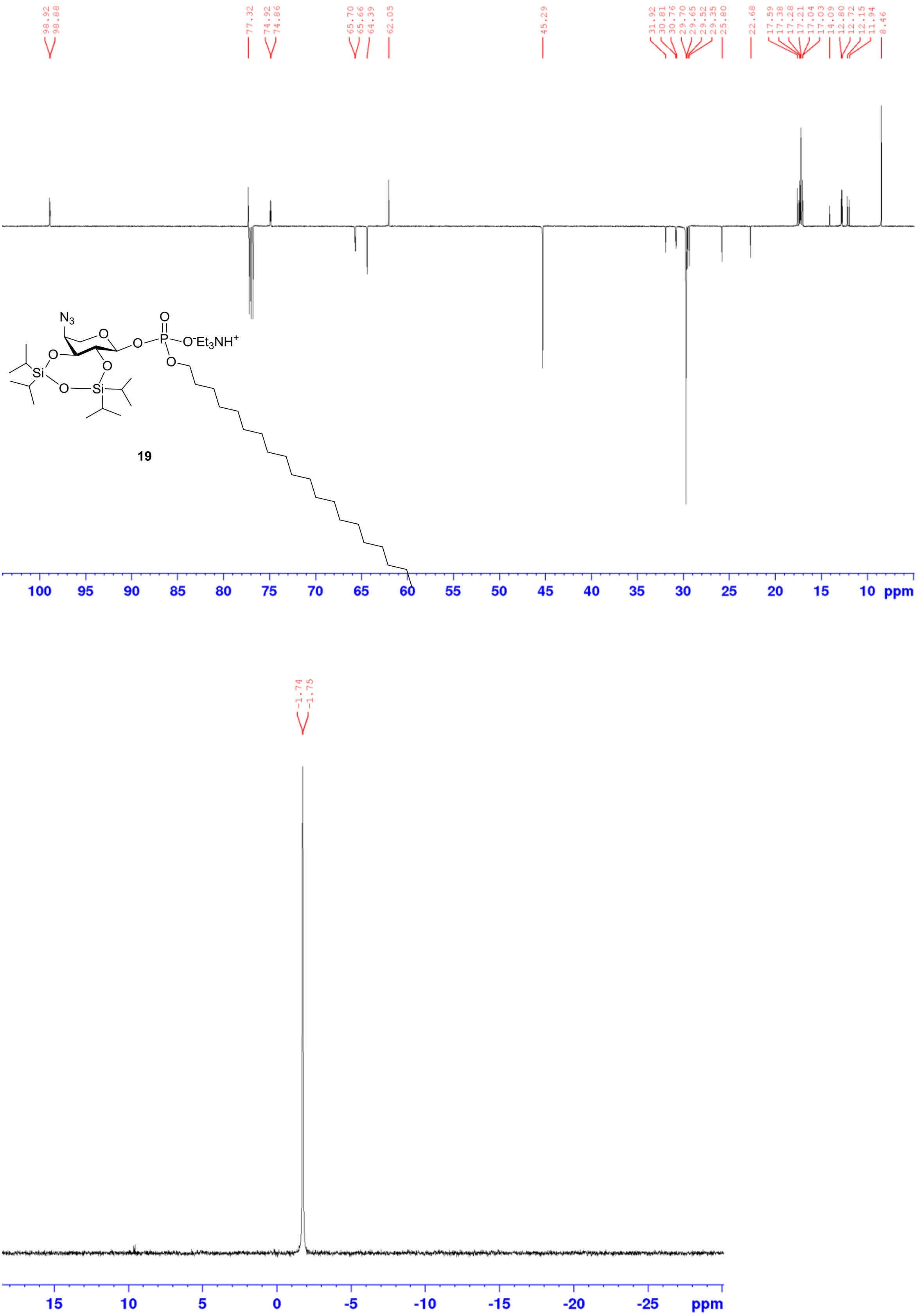

Fig. S23: ^{13}C and ^{31}P NMR spectrum of **19**.

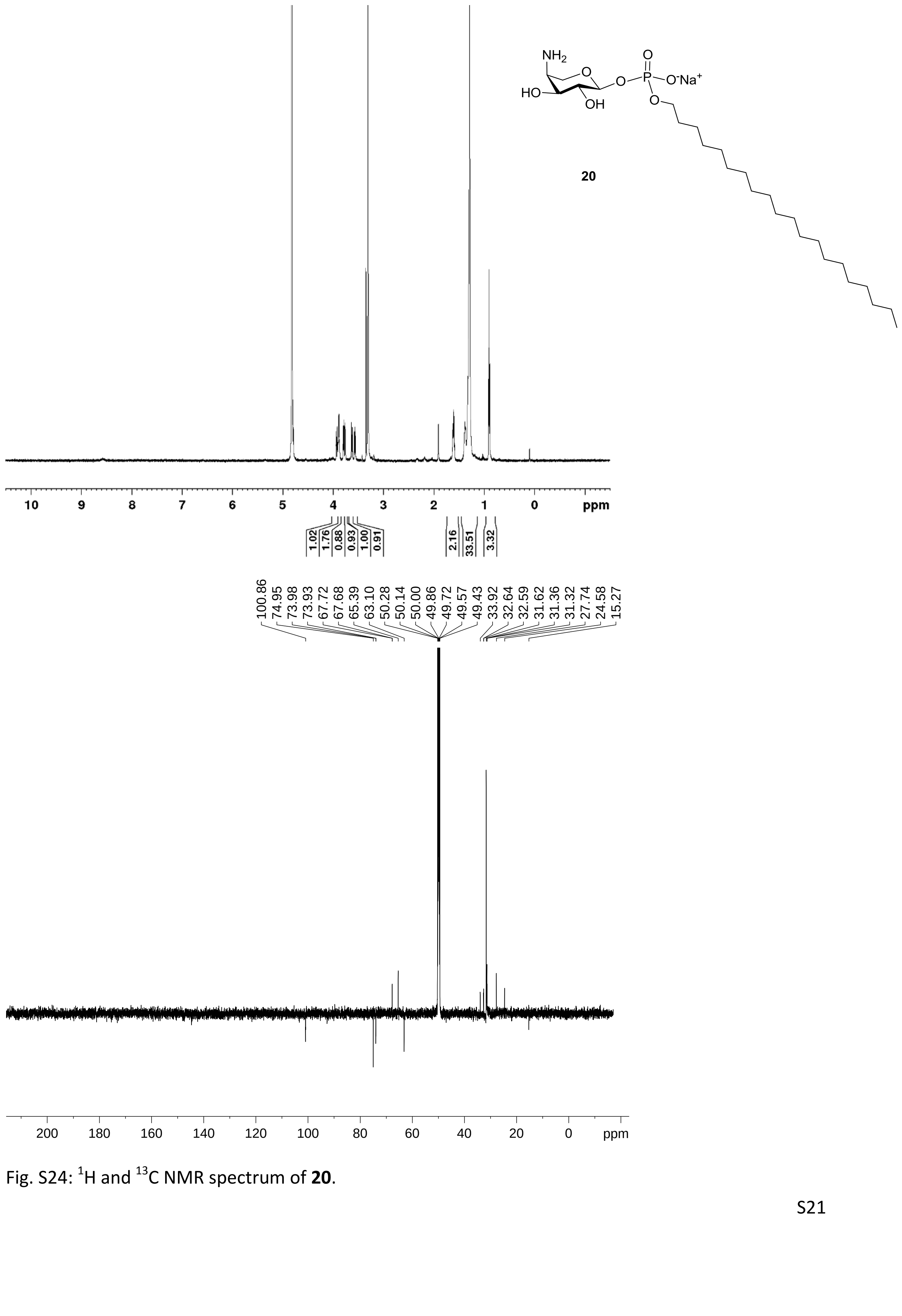

Fig. S24: ^{1}H and ^{13}C NMR spectrum of **20**.

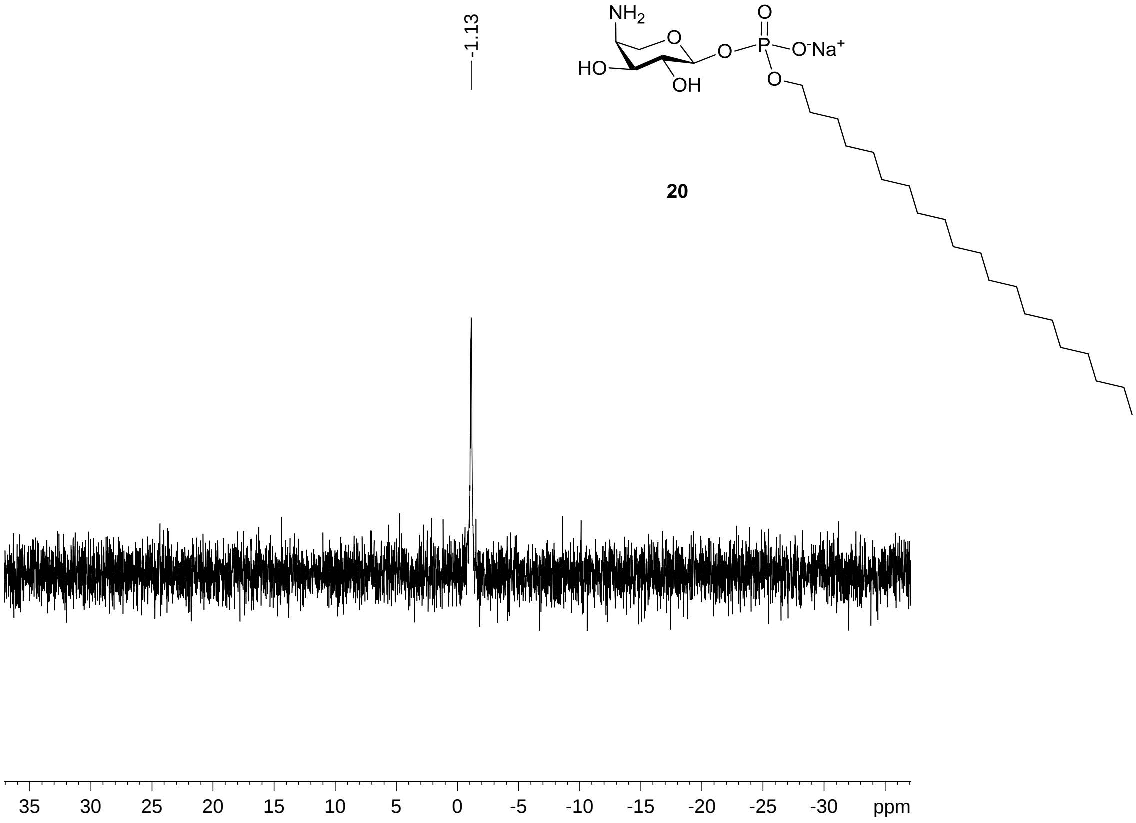

Fig S25: ^{31}P NMR spectrum of **20**.

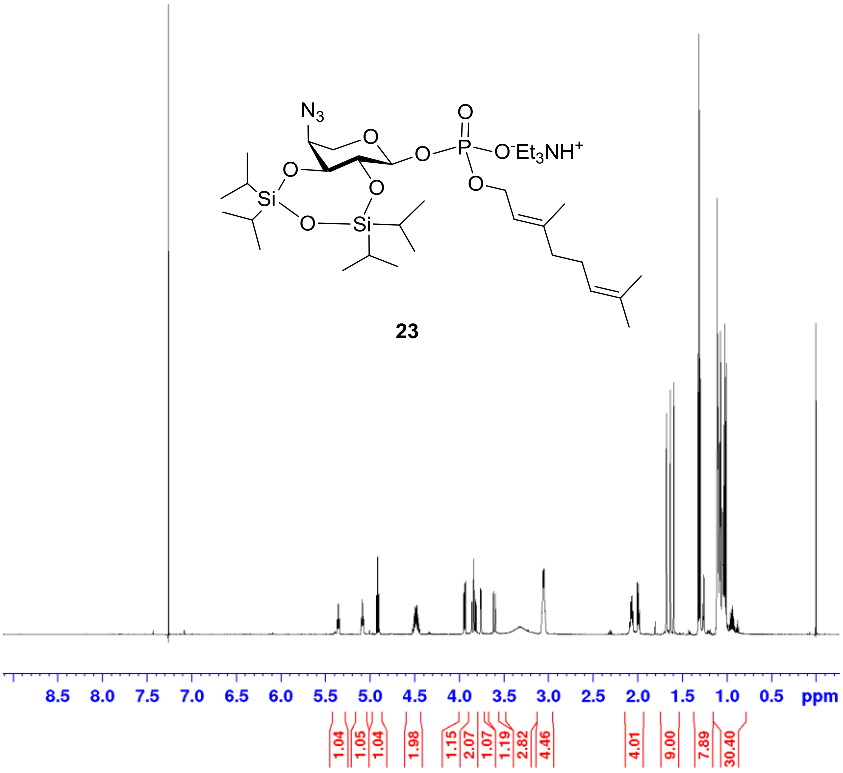

Fig S26: ^{1}H NMR spectrum of **23**.

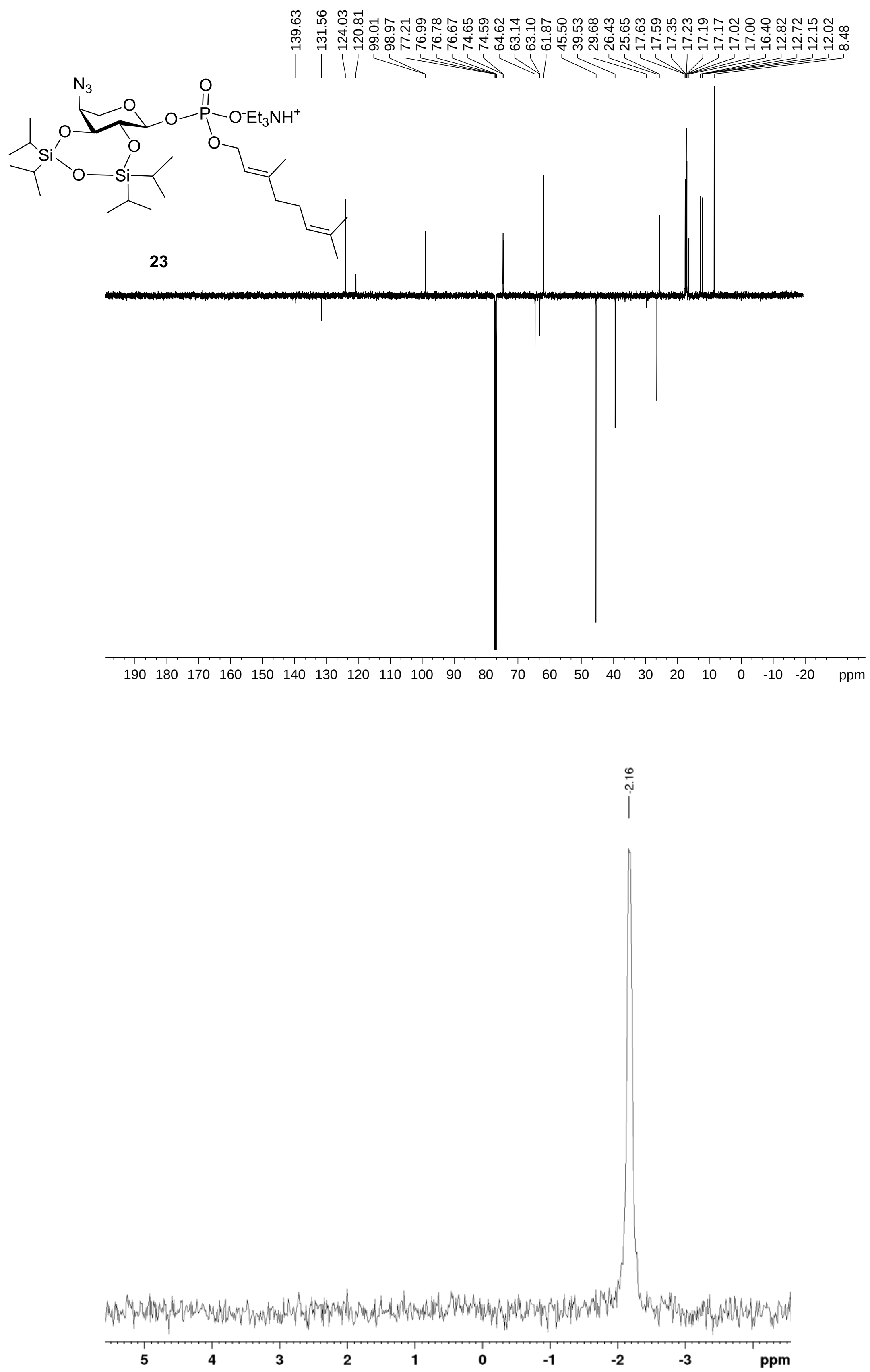

Fig. S27: ^{13}C and ^{31}P NMR spectrum of **23**.

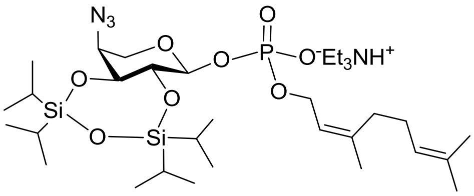

24

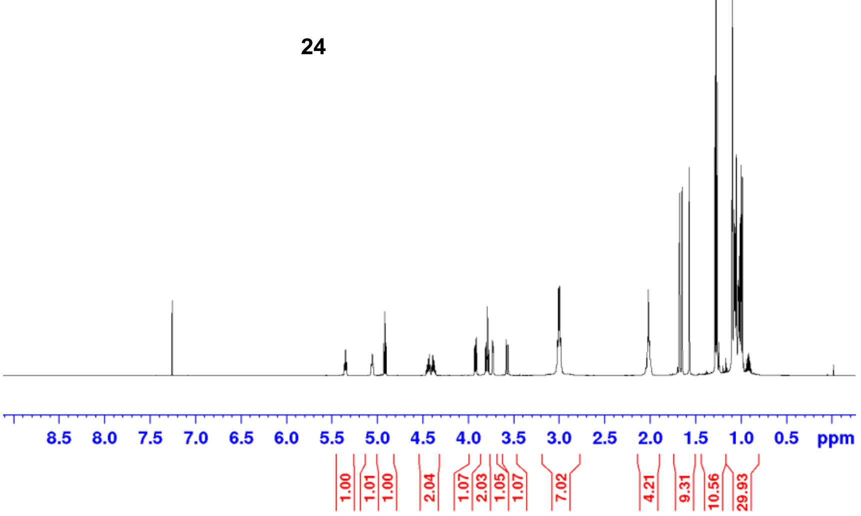

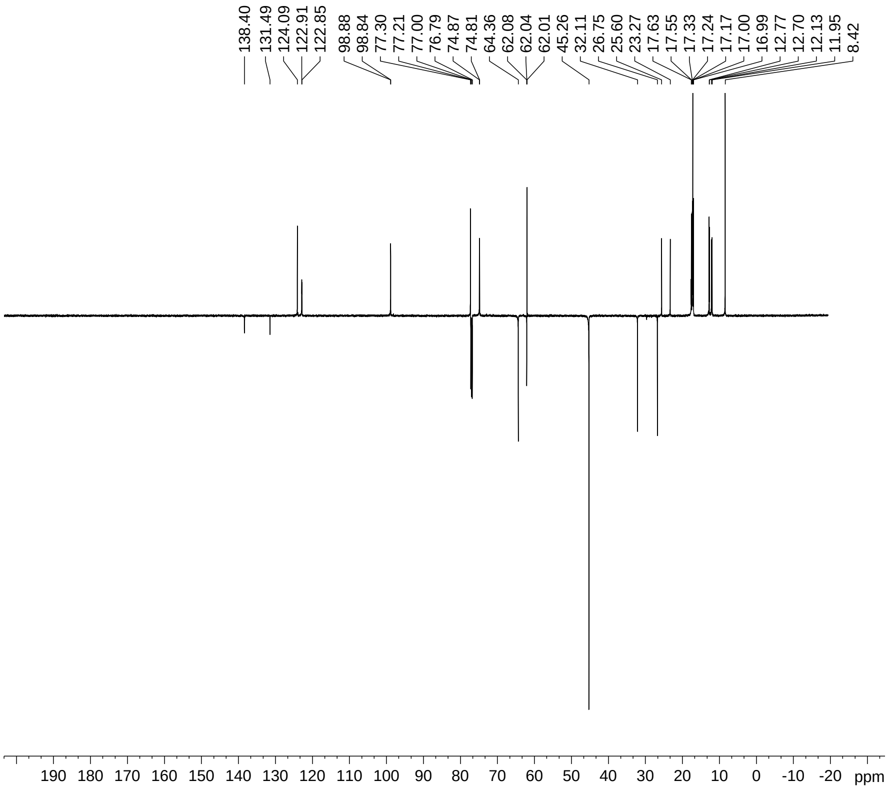

Fig. S28: ^{1}H and ^{13}C NMR spectrum of **24**.

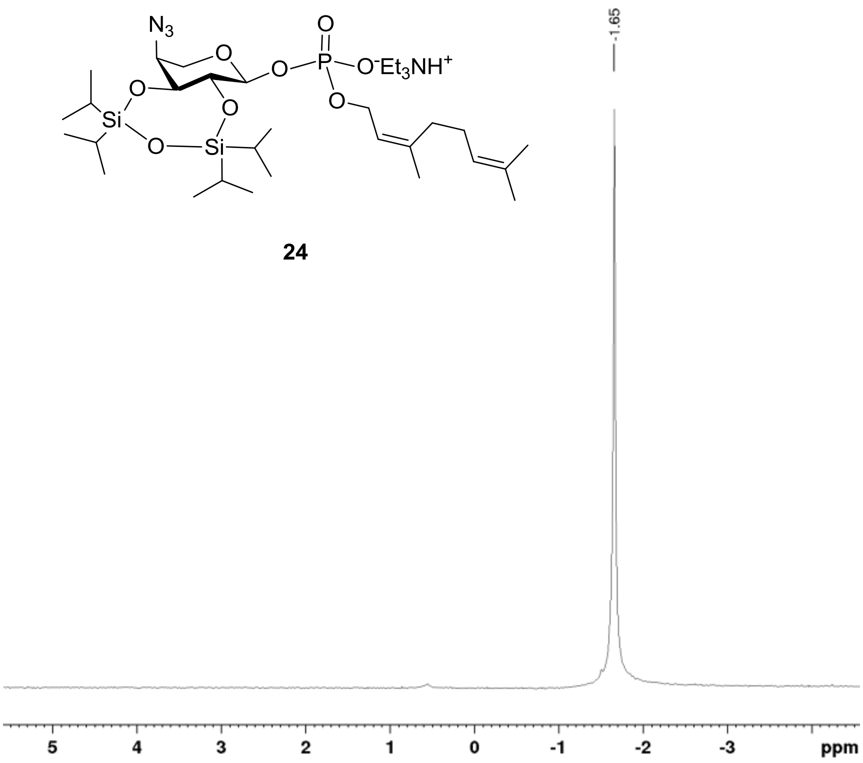

Fig. S29: ^{31}P NMR spectrum of **24**.

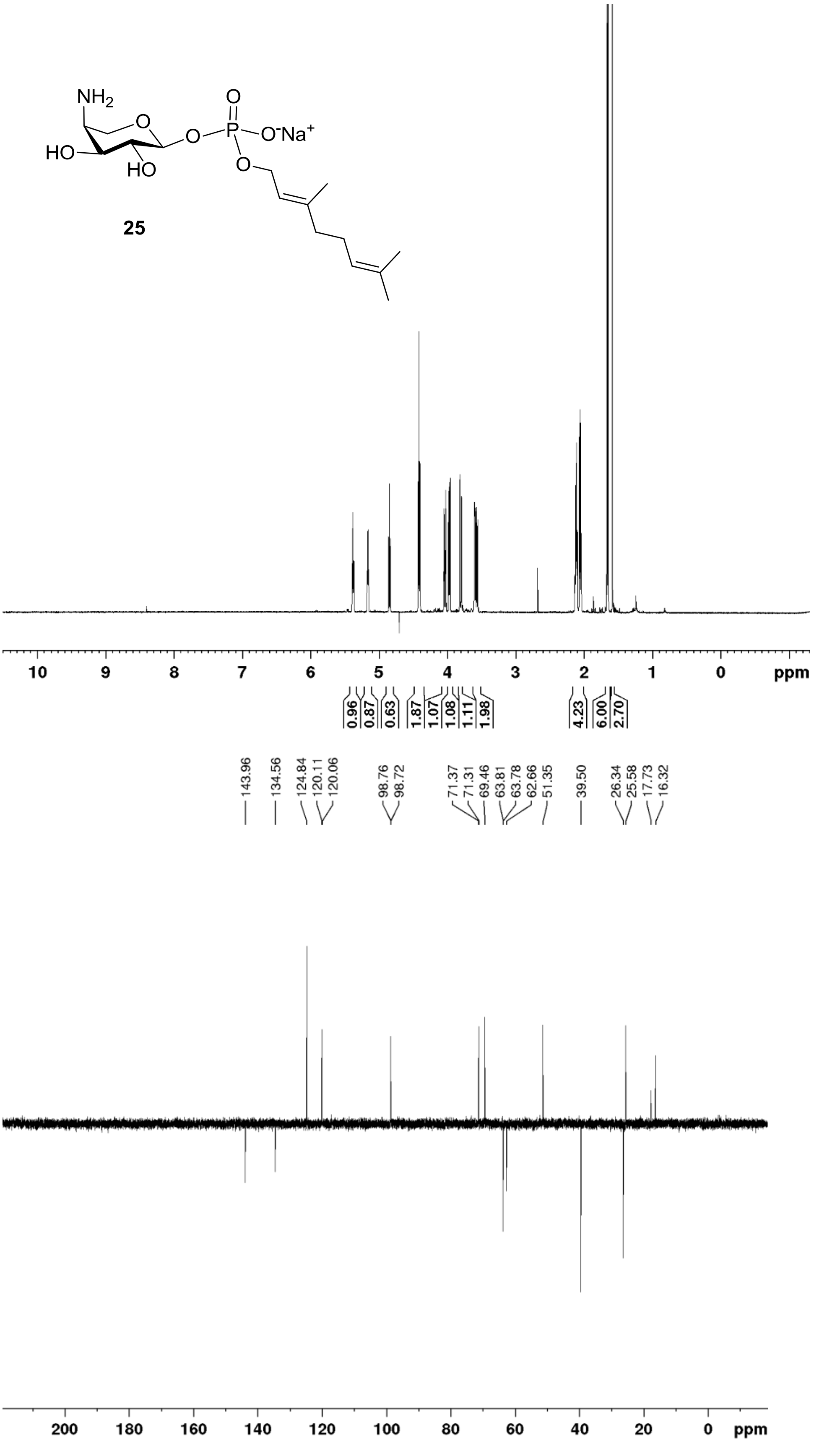

Fig. S30: ^{1}H and ^{13}C NMR spectrum of **25**.

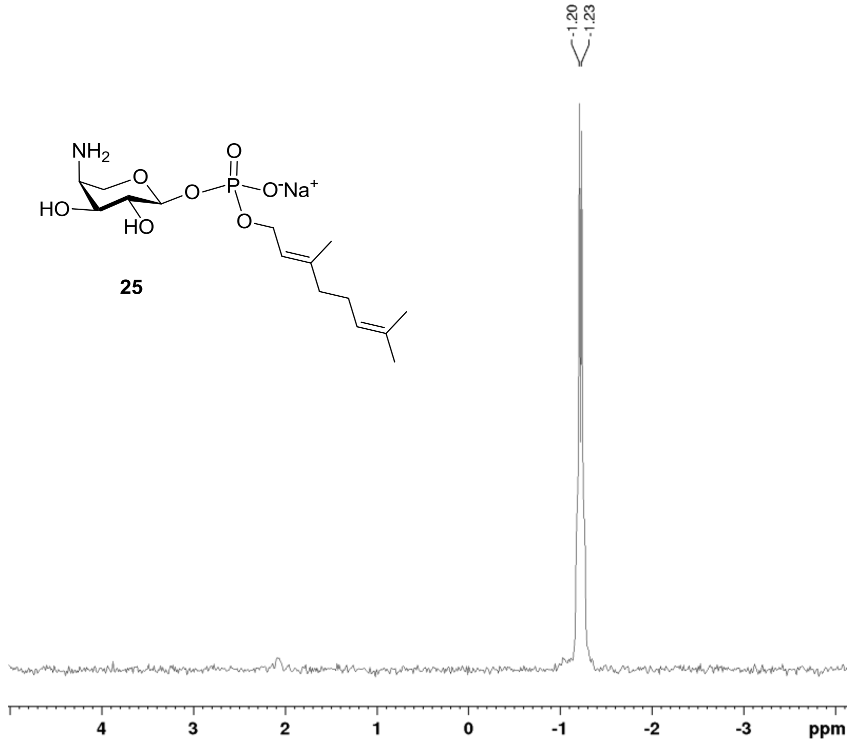

Fig. S31: ^{31}P NMR spectrum of **25**.

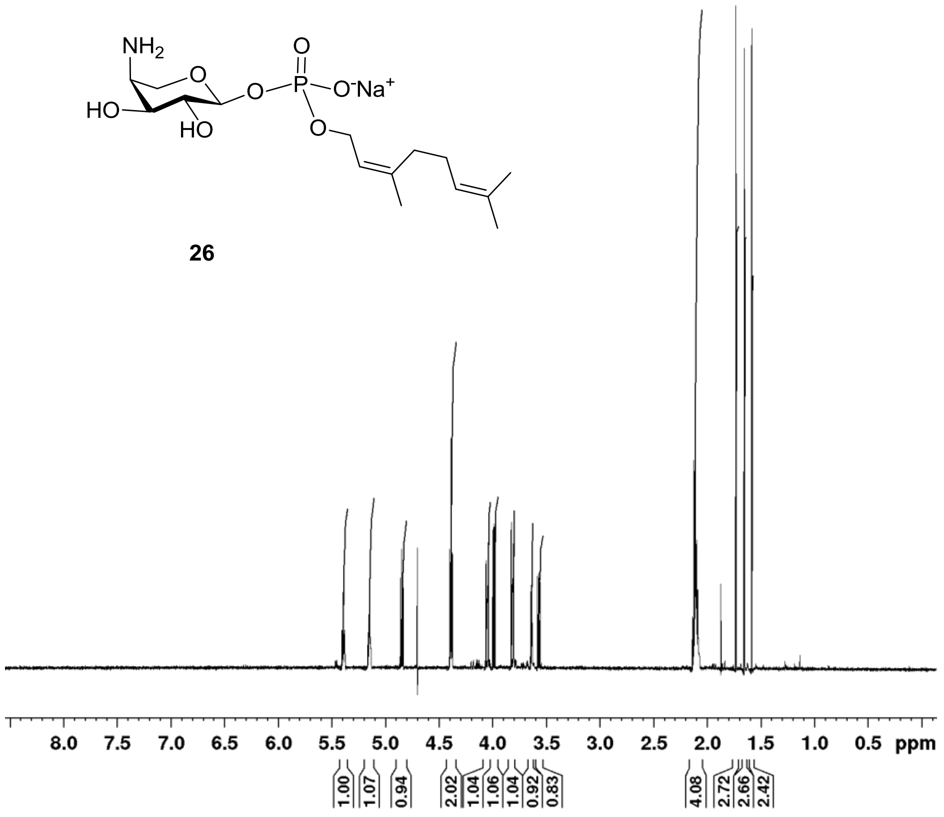

Fig. S32: ^{1}H NMR spectrum of **26**.

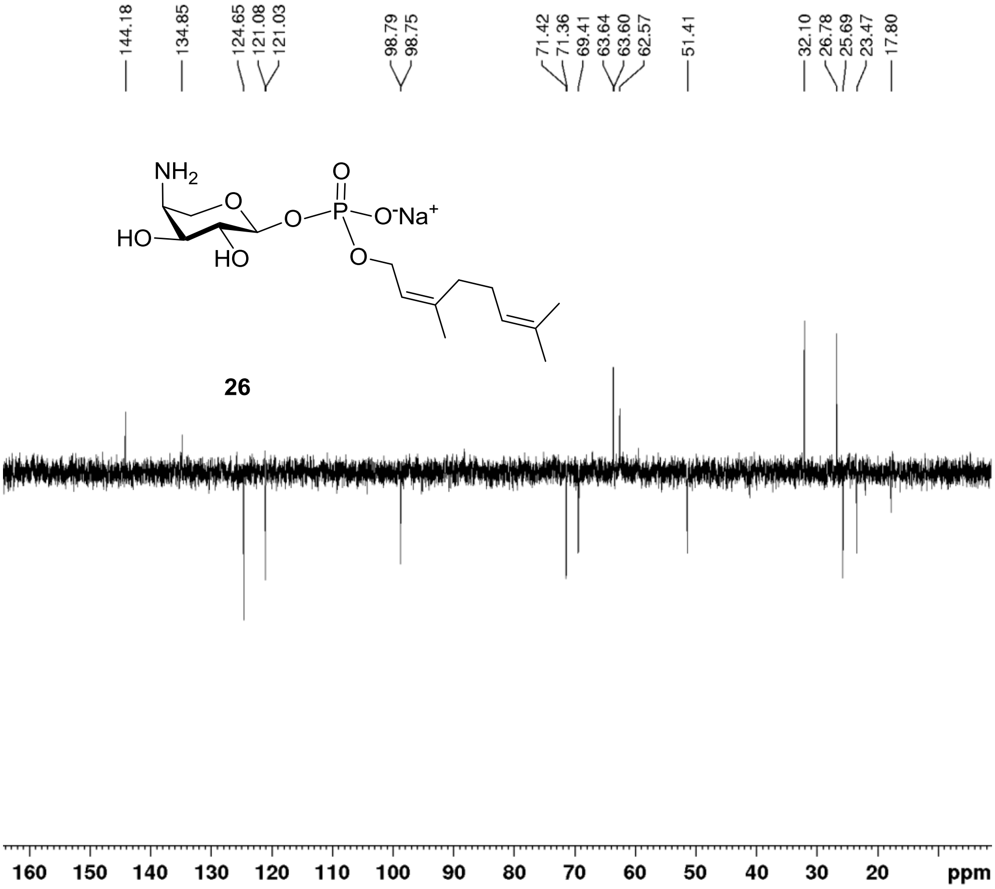

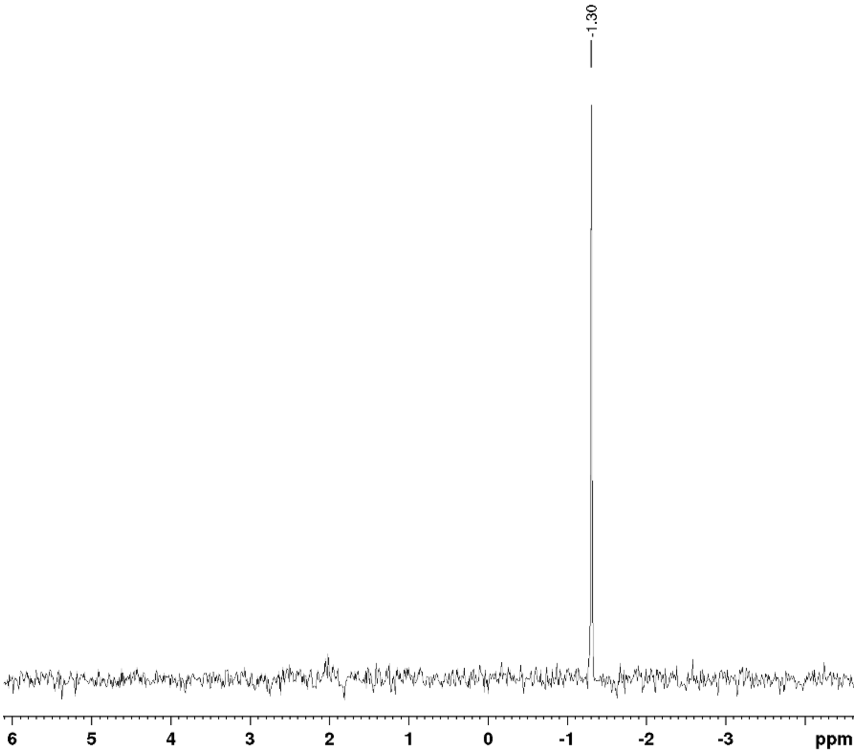

Fig. S33: ^{13}C and ^{31}P NMR spectrum of **26**.

Table S1. Strains and plasmids used for ArnT expression

Strain or Plasmids	Description	Reference
Strain		
E. coli		
DH5α	F^- ϕ80*lac*ZM15 *endA recA hsdR*($r^-_K m^-_K$) *supE thi gyrA relA Δ(lacZYA-argF)U169*	Laboratory stock
Plasmids		
pBAD24	Expression vector inducible with arabinose, for C-terminal FLAG-10x His fusions, Ap^R	Guzman et al. (1995)
pFT1	pBAD expressing ArnT-FLAG-10x His	Tavares-Carreon et al. (2015)

Peptide analysis

The sample was digested in solution. The proteins were S-alkylated with iodoacetamide, MeOH/chloroform precipitated and digested with Trypsin (Promega). The digested samples were loaded on a BioBasic C18 column (BioBasic-18, 150 x 0.32 mm, 5 µm, Thermo Scientific) using 80 mM ammonium formiate buffer as the aqueous solvent. A gradient from 5% B (B: 80% ACCN) to 42% B in 35 min was applied, followed by a 10min gradient from 42% B to 90% B that facilitates elution of large peptides, at a flow rate of 6 µL/min. Detection was performed with QTOF MS (Bruker maXis 4G) equipped with the standard ESI source in positive ion, DDA mode (= switching to MSMS mode for eluting peaks). MS-scans were recorded (range: 150-2200 Da) and the 6 highest peaks were selected for fragmentation. Instrument calibration was performed using ESI calibration mixture (Agilent).
The analysis files were converted (using Data Analysis, Bruker) to mgf files, which are suitable for performing a MS/MS ion search with ProteinScape (Bruker, MASCOT embedded). The files were searched against an *E. coli* database with the sequence of the protein of interest added.

The target protein was identified with a MASCOT score of 743.2 as the third highest scoring protein. Peptide near the N- and C-terminus were identified leading to the conclusion that the protein was expressed completely. 156 proteins were identified in total in the crude membrane extract.

POI sequence:

MHHHHHHSSGLVPRGSGMKETAAAKFERQHMDSPDLGTDDDDKAMADIGSEFMNDTPSRLPLNRITLVLLLVAL
AIVWFAPLGLRHLIPSDEGRYAEMAREMFVTGDWITPRYNGYKYFEKPPLQTWLNALTFAWFGIGEWQARLYTAV
ASFAGVLLVGYTGARLFNPLSGFLAAVVLASSPYWNLMGHFNALDMGLAFWMALSLCSLLLAQRPGLRPAAVRG
WMWACWAAMAFAVLSKGLVGLILPGAVLVLYTLVARDWALWKRLYLVSGLVIFFAIVTPWFVLVQQRNPEFFNF
FFIVQQFRRYLTPEQNRPGPLYYFVPVLLVGFLPWLSVAWQSIRHAVRMPRQPNGFAPMLVLLIWSAFIFLFFSASH
SKLISYVLPVAPALALIIGAYLPLMTADRFRRHLLGYLVFFVAAAFGIVFLAYQGDARTPNALYRAFQMWLYAGLAVA
AALTLVAAWLNRRAGVAAALATFGAAWLVFGTIGGTGHDEFGRYSSGALLAPAVRAELAKLPPDTPFYSIEMLDHT
FPFYMGHTTIMVQRQDELAFGISVEPNKWIPTIGEWITRWKQETHALAIMPPGQYDALVKEGVPMRVIARDNRR
VIVEKPQSESTARHDYKDDDDDKLEHHHHHHHHHH

References

1. L. M. Guzman, D. Belin, M. J. Carson, J. Beckwith, *J. Bacteriol.* **1995**, *177*, 4121–4130.
2. F. Tavares-Carreón, K. B. Patel, M. Valvano, *Scientific Rep.* **2015**, *5*, 10773.
